# Robust and efficient parameter estimation in dynamic models of biological systems

**DOI:** 10.1186/s12918-015-0219-2

**Published:** 2015-10-29

**Authors:** Attila Gábor, Julio R. Banga

**Affiliations:** BioProcess Engineering Group, IIM-CSIC, Eduardo Cabello 6, Vigo, 36208 Spain

**Keywords:** Parameter estimation, Dynamic models, Regularization, Global optimization, Overfitting

## Abstract

**Background:**

Dynamic modelling provides a systematic framework to understand function in biological systems. Parameter estimation in nonlinear dynamic models remains a very challenging inverse problem due to its nonconvexity and ill-conditioning. Associated issues like overfitting and local solutions are usually not properly addressed in the systems biology literature despite their importance.

Here we present a method for robust and efficient parameter estimation which uses two main strategies to surmount the aforementioned difficulties: (i) efficient global optimization to deal with nonconvexity, and (ii) proper regularization methods to handle ill-conditioning. In the case of regularization, we present a detailed critical comparison of methods and guidelines for properly tuning them. Further, we show how regularized estimations ensure the best trade-offs between bias and variance, reducing overfitting, and allowing the incorporation of prior knowledge in a systematic way.

**Results:**

We illustrate the performance of the presented method with seven case studies of different nature and increasing complexity, considering several scenarios of data availability, measurement noise and prior knowledge. We show how our method ensures improved estimations with faster and more stable convergence. We also show how the calibrated models are more generalizable. Finally, we give a set of simple guidelines to apply this strategy to a wide variety of calibration problems.

**Conclusions:**

Here we provide a parameter estimation strategy which combines efficient global optimization with a regularization scheme. This method is able to calibrate dynamic models in an efficient and robust way, effectively fighting overfitting and allowing the incorporation of prior information.

**Electronic supplementary material:**

The online version of this article (doi:10.1186/s12918-015-0219-2) contains supplementary material, which is available to authorized users.

## Background

Mathematical modelling is the central element in quantitative approaches to molecular and cell biology. The possible uses of quantitative modelling of cellular processes go far beyond explanatory and predictive studies [1, 2]. They provide a way to understand complex bio-systems [3, 4] and have given rise to systems biology as a new way of thinking in biological research [5]. Models in systems biology vary in their degree of network complexity and accuracy of representation [6]. Dynamic (i.e. kinetic) models offer the greatest degree of flexibility and accuracy to explain how physiological properties arise from the underlying complex biochemical phenomena. In fact, it has been argued that the central dogma of systems biology is that it is system dynamics that gives rise to the functioning and function of cells [7].

The use of kinetic models to understand the function of biological systems has already been successfully illustrated in many biological systems, including signalling, metabolic and genetic regulatory networks [8–16]. Further, dynamic model-based approaches have also been used to identify possible ways of intervention or (re-)design, such as in optimal experimental design [17–21], metabolic engineering [22] and synthetic biology [23, 24]. Other recent efforts have been focused on scaling-up, i.e. on the development and exploitation of large-scale (genome-scale) kinetic models [25], and ultimately, whole-cell models [26, 27].

Although nonlinear dynamical models have become the most common approach in systems biology, they have received relatively little attention in the statistical literature, especially when compared with other model types [28]. As a consequence, the area can be regarded as one of the most fertile fields for modern statistics [29]: it offers many opportunities, but also many important challenges [30].

One of the main challenges is the calibration of these dynamic models, also known as the parameter estimation problem. Parameter estimation aims to find the unknown parameters of the model which give the best fit to a set of experimental data. Parameter estimation belongs to the class of so called inverse problems [31, 32], where it is important to include both a priori (i.e. structural) and a posteriori (i.e. practical) parameter identifiability studies. In this way, parameters which cannot be measured directly will be determined in order to ensure the best fit of the model with the experimental results. This will be done by globally minimizing an objective function which measures the quality of the fit. This problem has received considerable attention, as reviewed in [19, 33–37]. It is also frequently described as the inverse problem, i.e., the inverse of model simulation from known parameters, considered the direct problem.

This inverse problem usually considers a cost function to be optimized (such as maximum likelihood), which in the case of nonlinear dynamic models must be solved numerically. Numerical data fitting in dynamical systems is a non-trivial endeavour, full of pitfalls (see, e.g. Chapter 4 in [38]). The inverse problem is certainly not exclusive of systems biology: it has been extensively studied in other areas, as reviewed in [39], each one contributing with somewhat different perspectives regarding the difficulties encountered and how to surmount them.

Here we would like to address two key pathological characteristics of the inverse problem which make it very hard: ill-conditioning and nonconvexity [19, 40, 41]. These concepts are intimately related with other similar notions developed independently in different communities [39]. For example, ill-conditioning can be related to the lack of identifiability arising from the model structure, and/or from information-poor data. Nonconvexity and multi-modality usually cause convergence to local solutions (local minima), which are estimation artefacts. Both are significant sources of concern that need to be properly addressed.

Due to the nonconvexity of the parameter estimation problem, there is a need for suitable global optimization methods [19, 36, 42–45]. Relying on standard local optimization methods can lead to local solutions, producing wrong conclusions: for example, one can incorrectly conclude that a novel kinetic mechanism is wrong because we are not able to obtain a good fit to the data, but the real reason might be that the method used simply converged to a local minima [46]. Indeed, a number of studies have described the landscape of the cost functions being minimized as rugged, with multiple minima [41, 44, 47]. It has been argued [48, 49] that local methods or multi-start local methods can be effective if properly used, but in our experience (and as we will show below) this only holds for relatively well-behaved problems, i.e. those with good initial guesses and tight bounds on the parameters. Therefore, in general, global optimization methods should be used in order to minimize the possibility of convergence to local solutions [19, 36, 42, 47].

The ill-conditioning of these problems typically arise from (i) models with large number of parameters (over-parametrization), (ii) experimental data scarcity and (iii) significant measurement errors [19, 40]. As a consequence, we often obtain overfitting of such kinetic models, i.e. calibrated models with reasonable fits to the available data but poor capability for generalization (low predictive value). In this situation, we are over-training the model such as we fit the noise instead of the signal. Therefore, overfitting damages the predictive value of the calibrated model since it will not be able to generalize well in situations different from those considered in the calibration data set. Overfitting might be behind most failures in model-based prediction and forecasting methods in many fields of science and engineering, and it has probably not received as much attention as it deserves (“the most important scientific problem you have never heard of”, in the words of Silver [50]).

Most mechanistic dynamic models in systems biology are, in principle, prone to overfitting: either they are severely over-parametrized, or calibrated with information-poor data, or both. However it is quite rare to find studies where a calibrated model is tested with a new data set for cross-validation (an example of exception would be the study of Zi and Klipp [51]). Further, as we will show below, over-parametrization and lack of information are not the only factors to induce overfitting: model flexibility plays an equally important role.

The paper is structured as follows. First, we consider the statement of the inverse problem associated to kinetic models of biological systems, and we focus on its ill-conditioning and nonconvexity, reviewing the state of the art. We then present a strategy to analyse and surmount these difficulties. In the case of nonconvexity, we present a suitable global optimization method. In the case of ill-conditioning and overfitting, we consider the use of regularization techniques. Our strategy is then illustrated with a set of seven case studies of increasing complexity, followed by a detailed discussion of the results. Finally, we present practical guidelines for applying this strategy considering several scenarios of increasing difficulty.

## Methods

### Parameter estimation in dynamic models

#### Mathematical model

Here we will consider dynamic models of biological systems described by general nonlinear differential equations. A common case is that of kinetic models. For the case of biochemical reaction networks, and under the assumption of well-mixed compartments and large enough concentration of molecules (so stochastic effects are negligible), kinetic models describe the concentration dynamics using nonlinear deterministic ordinary differential equations. One of the most general form of these equations is given by the deterministic state-space model:

(1) $$\begin{array}{*{20}l}  \frac{dx(t,\theta)}{dt} &= f(t,x(t,\theta),u(t),\theta), \end{array} $$

(2) $$\begin{array}{*{20}l}  y(x,\theta) &= g(x(t,\theta),\theta), \end{array} $$

(3) $$\begin{array}{*{20}l}  x(t_{0}) & = x_{0}(\theta), \quad t \in [t_{0},\,t_{f}] \enspace, \end{array} $$

where $x \in \mathcal {R}^{N_{x}}$ is the state vector (often concentrations), the $f(\cdot):\mathcal R^{1\times N_{x} \times N_{u}\times N_{\theta }} \mapsto \mathcal {R}^{N_{x}}$ vector function is constructed from the reaction rate functions and stimuli *u*(*t*). The *N*_*θ*_ dimensional parameter vector *θ* contains the positive parameters of the reaction rate functions–for example the reaction rate coefficients, Hill exponents, dissociation constants, etc.–, but can also include the initial conditions. The observation function $g(\cdot):R^{N_{x} \times N_{\theta }} \mapsto \mathcal {R}^{N_{y}}$ maps the state variables to the vector of observable quantities $y \in \mathcal R^{N_{y}}$, these are the signals that can be measured in the experiments. The observation functions may also directly depend on estimated parameters for example on scaling parameters. When multiple experiments in different experimental conditions are considered, typically the same model structure is assumed, but the initial conditions and stimuli are adapted to the new conditions.

#### Calibration data, error models and cost functions

We assume, that the data is collected in multiple experiments at discrete time points *t*_*i*_∈[*t*_0_, *t*_*f*_], thus the model outputs must be discretized accordingly. Let us denote the model prediction at time *t*_*i*_, of the *j*-th observed quantity in the *k*-th experiment by *y*_*ijk*_. Due to measurement errors the true signal value is unknown and a noise model is used to express the connection between the true value *y*_*ijk*_ and measured data $\tilde y_{\textit {ijk}}$.

In general, the type and magnitude of the measurement error depend on both the experimental techniques and the post-processing of the data. For example, blotting techniques are generally used to obtain quantitative data for gene expression levels or protein abundance. These data is assumed to contaminated by either additive, normally distributed random error (noise) or by multiplicative, log-normally distributed noise. Rocke and Durbin [52] concluded that the gene expression data measured by DNA micro-arrays or oligonucleotic arrays contains both additive and multiplicative error components. Similar conclusions were reported by Kreutz and co-authors [53] for protein measurements using immunoblotting techniques. In this context, there are both experimentation techniques (for example gel randomisation [54]) and mathematical procedures (general logarithmic transformation [55, 56]) to ensure proper data pre-processing for model calibration.

##### Maximum likelihood and cost function

Assuming that the transformed measurements (which is still denoted by $\tilde y$ for consistency) are contaminated by additive normally distributed uncorrelated random measurement errors –i.e. $\tilde y_{\textit {ijk}} = y_{\textit {ijk}}(x(t_{i}),\theta)+\epsilon _{\textit {ijk}}$ where $ \epsilon _{\textit {ijk}} \sim \mathcal {N}\left (0,\sigma _{\textit {ijk}}^{2}\right)$ is the random error with standard deviation *σ*_*ijk*_ and $\tilde y_{\textit {ijk}}$ is the measured value–, the estimation of the model parameters is formulated as the maximization of the likelihood of the data [57, 58]

(4) $$ \begin{aligned}  \mathcal{L}(\,\tilde y ~|~\theta) &= \prod_{k=1}^{N_{\mathrm{e}}} \prod_{j=1}^{N_{\text{\textit{y,k}}}} \prod_{i=1}^{N_{\text{\textit{t,k,j}}}}\frac{1}{\sqrt{2\pi\sigma_{\text{ijk}}^{2}} }\\ &\quad\times\exp\left(-\frac{1}{2}\frac{(\,y_{\text{ijk}}(x(t_{i},\theta),\theta)-\tilde y_{\text{ijk}})^{2}}{\sigma_{\text{ijk}}^{2}}\right) \enspace, \end{aligned}  $$

where *N*_e_ is the number of experiments, *N*_*y,k*_ is the number of observed compounds in the *k*-th experiment, and *N*_*t,k,j*_ is the number of measurement time points of the *j*-th observed quantity in the *k*-th experiment. The total number of data points is $N_{\mathrm {D}}=\sum _{k=1}^{N_{\mathrm {e}}} \sum _{j=1}^{N_{\text {\textit {y,k}}}} \sum _{i=1}^{N_{\text {\textit {t,k,j}}}}1$. The maximization of the likelihood function (4) is equivalent to the minimization of the weighted least squares cost function [58]

(5) $${}  Q_{\text{LS}}(\theta) = \sum_{k=1}^{N_{\mathrm{e}}} \sum_{j=1}^{N_{\text{\textit{y,k}}}} \sum_{i=1}^{N_{\text{\textit{t,k,j}}}}\left(\frac{y_{\text{ijk}}(x(t_{i},\theta),\theta)-\tilde y_{\text{ijk}}}{\sigma_{\text{ijk}}}\right)^{2} = R(\theta)^{T}R(\theta)\enspace,  $$

where the residual vector $R(\cdot):{{R}^{{N}_{\theta }}}\to {{R}^{{N}_{\mathrm {D}}}}$ is constructed from the squared terms by arranging them to a vector. With this, the model calibration problem can be stated as the well-known nonlinear least-squares (NLS) optimization problem:

(6) $$ \begin{aligned}  \underset{\theta}{\text{minimize }} && ~ Q_{\text{LS}}(\theta) &= R(\theta)^{T}R(\theta)\\ \text{subject to} && ~\theta_{\text{min}} \leq & \theta \leq \theta_{\text{max}}\enspace,\\ && \frac{dx(t,\theta)}{dt} &= f(u(t),x(t,\theta),\theta)\enspace,\\ && y(x,\theta) &= g(x(t,\theta),\theta)\enspace,\\ && x(t_{0}) &= x_{0}(\theta), \quad t \in [t_{0},\,t_{f}] \enspace. \end{aligned}  $$

A $\hat \theta $ vector that solves this optimization problem is called the *optimal parameter vector*, or the *maximum likelihood estimate* of the model parameters. However, note that the uniqueness of the solution is not guaranteed, which results in the ill-posedness of the calibration problem, as discussed later.

##### Post-analysis

Post-analysis of calibrated models is an important step of the model calibration procedure. Classical methods to diagnose the identifiability and validity of models, and the significance and determinability of their parameters are described in e.g. [34]. Most of these methods, such as the *χ*^2^ goodness of fit test, or the distribution and correlation analysis of the residuals by, for example, the Shapiro-Wilk test of normality, assume that the errors follow a normal distribution, so they should be used carefully (i.e. in many real problems such assumption might not hold). Similarly, the computation of the covariance and correlation of the parameters ([59, 60]) and the computation of confidence regions of the model predictions [48] are usually performed based on the Fisher information matrix (FIM). But the FIM has important limitations, especially for nonlinear models: it will only give a lower bound for the variance, and symmetric confidence intervals. Nonparametric methods such as the bootstrap [61] are much better alternatives. Here, rather than focusing our post-analysis using these metrics, we will focus on examining the generalizability of the fitted model. In particular, below we will make extensive use of cross-validation methods, which are rather well-known in system identification to avoid overfitting [62, 63], but which have been very rarely used in the systems biology field.

### Global optimization method

It is well-known that the cost function (5) can be highly nonlinear and nonconvex in the model parameters (see e.g. [38, 41, 44, 47]. Many efficient *local optimization algorithms* have been developed to find the solution of nonlinear least squares problems, including Gauss-Newton, Levenberg-Marquardt and trust-region methods [38]. These local methods, (and others like truncated and quasi-Newton) are especially efficient when provided with high quality first (gradient, Jacobian) and second order (Hessian) information via parametric sensitivities [64, 65]. However, in this type of problems they will likely converge to local solutions close to the initial guess of the parameters.

Multi-start local methods (i.e. performing multiple runs initiating local optimizers from a set of initial guesses distributed in the search domain) have been suggested as more robust alternatives. Typically the set of initial guesses is generated inside the parameter bounds either randomly or by a more sophisticated sampling scheme, such as Latin hypercube sampling [66]. Multi-start methods have shown good performance in certain cases, especially when high-quality first order information are used and the parameter search space is restricted to a relatively small domain [48, 49]. However, other studies [44, 67, 68] have shown that multi-start methods become inefficient as the size of the search space increases, and/or when the problem is highly multimodal, since many of the local searches will explore the same local basins of attraction repeatedly.

Therefore, a number of researches have supported the use of global optimization as a better alternative. However, the current state of the art in global optimization for this class of problems is still somewhat unsatisfactory. Deterministic global optimization methods [43, 46, 69–71] can guarantee global optimality but their computationally cost increases exponentially with the number of estimated parameters. Alternatively, stochastic and metaheuristic methods [19, 36, 44, 47, 72, 73] can be used as more practical alternatives, usually obtaining adequate solutions in reasonable computation times, although at the price of no guarantees. In the context of metaheuristics, hybrids (i.e. combinations) with efficient local search methods have been particularly successful [67, 68, 72, 74–77].

Here we have extended the enhanced scatter search (eSS) metaheuristic presented by Egea et al. [75]. Our extension of this method, which we will call eSS2, makes use of elements of the scatter search and path re-linking metaheuristics, incorporating several innovative mechanisms for initial sampling (including Latin hypercube), an update method which improves the balance between intensification (local search) and diversification (global search), and new strategies to avoid suboptimal solutions. In eSS2 we have also incorporated several methodological and numerical improvements with the double aim of (i) increasing its overall robustness and efficiency, (ii) avoiding the need of tuning of search parameters by the user (a drawback of many metaheuristics). These improvements can be summarized as follows:

- *Efficient local search* exploiting the structure of the nonlinear least squares problem: after extensive comparisons of local solvers, we selected the adaptive algorithm NL2SOL [78]. This is a variant of the Gauss-Newton method that utilizes the Jacobian of the residual vector (see Additional file 1) to approximate and iteratively upgrade the parameter vector. In order to increase its efficiency, we also provide it with high quality gradient information (see below), resulting in speed-ups of up to 20 times.
- *Efficient integration* of the initial value problem and its extension with parametric forward sensitivity equations using the CVODES solver [79], providing it with the Jacobian of the dynamics.
- *Fast computation*: although the global solver eSS is implemented in Matlab, the integration of the initial value problem is done in C in order to speed-up the computations up to 2 orders of magnitude.
- *Robust default tuning*: metaheuristics require the user to set a number of search parameter values which usually require a number of time-consuming initial trial runs. In the method proposed here, we have made sure that the default search parameters work well without the need of any tuning, which is an additional important advantage. These settings are given in Table S.6.1 in Additional file 1.

### Regularization

Regularization methods have a rather long history in inverse problems [80] as a way to surmount ill-posedness and ill-conditioning. The regularization process introduces additional information in the estimation, usually by penalizing model complexity and/or wild behaviour. Regularization is related to the parsimony principle (Ockham’s razor), i.e. models should be as simple as possible, but not simpler [81, 82]. It also has links with Bayesian estimation in the sense that it can be regarded as a way of introducing prior knowledge about the parameters [83]. Regularization aims to make the problem less complex (more regular), i.e. to ensure the uniqueness of the solution [84], to reduce the ill-conditioning and to avoid model overfitting. However, one crucial step is the proper balancing of prior knowledge and information in the data, also known as the tuning of the regularization [85].

Regularization has been mainly used in fields dealing with estimation in distributed parameter systems, such as tomography (with applications in e.g. geophysics and medicine) and other image reconstruction techniques. Recently, it has enjoyed wide success in machine learning [86], gaining attention from the systems identification area [87]. However, the use of regularization in systems biology has been marginal [88], especially regarding mechanistic (kinetic) nonlinear models. Bansal et al. [89] compared Tikhonov and truncated singular value decomposition regularization for the linear regression model of green fluorescent protein reporter system to recover transcription signals from noisy intensity measurements. Kravaris et al. [40] compared the theoretical aspects of parameter subset estimation, Tikhonov and principal component analysis based regularization, also in a linear model framework. Wang and Wang [90] presented a two stage Bregman regularization method for parameter estimations in metabolic networks. A clear conclusion from these studies is that, for nonlinear inverse problems, there is no general recipe for the selection of regularization method and its tuning. Further, it is known that even for linear systems, choosing a method from the plethora of existing techniques is non-trivial [85].

Here we want to investigate the role that regularization can play regarding the calibration of nonlinear kinetic models. First of all, we need to address to question of which type of regularization should we use, and how to tune its parameters. Second, since kinetic models often have a fixed and rather stiff nature (as opposed to the flexibility of e.g. neural networks, as used in machine learning), it is a priori unclear if regularization can really help to avoid overfitting and enhance the predictive value of the calibrated model. Third, since most dynamic models in systems biology are severely over-parametrized, we want to explore its capabilities for systematic balancing the effective number of fitted parameters based on the available calibration data. Fourth, we want to evaluate the impact of regularization on the convergence properties of the global optimization solvers.

In order to answer these questions, here we present a critical comparison of a wide range of regularization methods applicable to nonlinear kinetic models. We then detail a procedure with guidelines for regularization method selection and tuning. Finally, we use numerical experiments with challenging problems of increasing complexity to illustrate the usage and benefits of regularization, addressing the questions above.

#### Statement of the regularized estimation

We consider penalty type regularization techniques [80], which include a penalty *Γ*(*θ*) in the original objective function (5), which results in the following regularized optimization problem:

(7) $$ \begin{aligned}  \hat \theta_{\alpha}\leftarrow\underset{\theta}{\text{minimize }} && ~ Q_{\mathrm{R}}(\theta) &= Q_{\text{LS}}(\theta)+\alpha \Gamma(\theta)\\ \text{subject to} && ~\theta_{\text{min}} \leq & \theta \leq \theta_{\text{max}}\enspace,\\ && \frac{dx(t,\theta)}{dt} &= f(u(t),x(t,\theta),\theta)\enspace,\\ && y(x,\theta) &= g(x(t,\theta),\theta)\enspace,\\ && x(t_{0}) &= x_{0}(\theta), \quad t \in [t_{0},\,t_{f}] \enspace. \end{aligned}  $$

Here $\alpha \in \mathcal {R}_{+}$ is the non-negative regularization parameter and $\Gamma (\cdot):\mathcal {R^{N_{\theta }}\to \mathcal {R}}$ is the regularization penalty function. When the solution of the original problem (*α*=0) is ill-posed, one has to incorporate some a priori assumption, which makes the estimation well posed. It is assumed that the penalty function *Γ*(*θ*) is well conditioned and has a unique minimum in the parameters. Thus, as the regularization parameter *α*→*∞* the optimization (7) is well-posed but highly biased by the a priori assumption, and when *α*=0 one obtains the original, ill-posed estimation problem. Therefore the role of the regularization parameter *α* is to properly balance the information of the data and the prior knowledge. However, this is a non-trivial task even for linear problems, as we will discuss below. Besides, there are many approaches to formulate the penalty function, among which the Tikhonov regularization [80], Least Absolute Shrinkage and Selection Operator (LASSO) regularization [91], the elastic net [92] and the entropy based methods [90, 93] are the most frequently used.

Determining the proper regularization parameter requires multiple solutions of the regularized optimization problem (7), therefore the computational efficiency is also crucial. Here we chose the Tikhonov regularization framework in order to match the form of the penalty to the least squares formalism of the objective function. In this case the least squares cost function can be simply augmented by the quadratic penalty function

(8) $$  \Gamma(\theta) = \left(\theta-\theta^{\text{ref}}\right)^{T}W^{T}W\left(\theta-\theta^{\text{ref}}\right),  $$

where $W\in \mathcal {R}^{N_{\theta } \times N_{\theta }}$ is a diagonal scaling matrix and $\theta ^{\text {ref}}\in \mathcal {R}^{N_{\theta }}$ is a reference parameter vector. In the special case, when *W* is the identity matrix, we call the scheme as the *non-weighted Tikhonov regularization* scheme (or shortly as Tikhonov regularization). If the *θ*^ref^ is the null-vector, the corresponding regularization scheme is often referred as *ridge regularization*.

#### Scenarios based on prior information

Kinetic models can overfit the data leading to poor generalizability. Here we propose using prior knowledge to select the most appropriate regularization method to avoid such overfit. Based on the level of confidence in this prior knowledge, we can consider three possible scenarios:

- *Worst case scenario*, where we have absolutely no prior information about the parameter values, typically resulting in very ample bounds and random initial guesses for the parameters.
- *Medium case scenario*, where there is some information about the parameters and their bounds.
- *Best case scenario*: the situation where a good guess of the parameters is at hand.

Below we will provide, for each scenario, robust recommendations regarding the regularization method to use and its tuning.

### Prediction error

In order to evaluate the performance of the calibrated model, we will use cross-validation [63, 94, 95], where the calibrated model is used to predict a yet unseen set of data, computing the prediction error. A good model should not only fit well the calibration data, but it also should predict well cross-validation data (without re-calibrating the model), i.e. it should be generalizable.

In this section we utilize the bias-variance decomposition of the prediction error and show when and how regularization can lead to smaller prediction error. First, let us introduce the subscript $\mathcal {C}$ for the calibration data and the subscript $\mathcal {V}$ for the validation data. For notational simplicity we consider only one experiment and only one observable for both of the calibration and validation scenario, but it is straightforward to generalize for multiple experiments and observables by the notion of normalized mean squared error (see Additional file 1). The expected prediction error (PE) for the validation data can be written as

(9) $$ \text{PE} = {\mathbb{E}}_{\mathcal{V},\mathcal{C}} { \left[ (\tilde y_{\mathcal{V}} -\hat y_{\mathcal{V}}(\hat\theta_{\mathcal{C}}))^{2}\right]}  $$

where $\tilde y_{\mathcal {V}}$ is the validation data, $\hat \theta _{\mathcal {C}}$ is the estimated parameter vector based on the calibration data and $\hat y_{\mathcal {V}}(\hat \theta _{\mathcal {C}})$ is the model predictions for the validation data. The prediction error depends on the calibration data –different calibration data would result in different estimated parameters $\hat \theta _{\mathcal {C}}$– and also depends on the validation data. Thus the expectation is taken over the distribution of the calibration and the validation data. The measurement error in the calibration data and in the validation data is often independent, leading to the well-known (see for example [62, 87, 96]) bias-variance decomposition of expected prediction error as

(10) $$ \begin{aligned}  \text{PE} &= \underbrace{{\mathbb{E}}_{\mathcal{V}}{\left[\left(y_{\mathcal{V}} - {\mathbb{E}}_{\mathcal{C}}{\left[\hat{y}_{\mathcal{V}} \left(\hat{\theta}_{\mathcal{C}}\right)\right]}\right)^{2}\right]}}_{\text{Bias}^{2}} \\ &\quad+ \underbrace{{\mathbb{E}}_{\mathcal{V}}{\left[\left(\hat{y}_{\mathcal{V}} (\hat{\theta}_{\mathcal{C}}) - {\mathbb{E}}_{\mathcal{C}}{\left[\hat{y}_{\mathcal{V}} \left(\hat{\theta}_{\mathcal{C}}\right)\right] }\right)^{2}\right]}}_{\text{Variance}} +\, {\mathbb{E}}_{\mathcal{V}}\left[{\epsilon^{2}}\right]. \end{aligned}  $$

Here, the first term corresponds to the squared bias of the calibrated model predictions from the true validation data $y_{\mathcal {V}}$, the second term is the variance of the model prediction, and the third term is the contribution of the measurement error ${\mathbb {E}}_{\mathcal {V}}\left [{\epsilon ^{2}}\right ] = \sigma ^{2}$.

**The variance term** The variance of the prediction is due to the uncertainty in the parameter estimates. This uncertainty can be especially large if the calibration data is scarce and the number of data points is close to the number of parameters. The variance term can be expressed for unbiased estimates [97] as

(11) $$  \text{Variance} = \frac{N_{\theta}}{N_{D}}\sigma^{2},  $$

where *N*_*θ*_ is the number of estimated parameters and *N*_*D*_ is the number of calibration data. Each estimated parameter contributes by $\frac {\sigma ^{2}}{N_{D}}$ to the prediction error, thus a model with fewer calibrated parameters would result smaller variance. For biased estimates the prediction variance becomes

(12) $$  \text{Variance} = \frac{N_{\theta}^{\text{eff}}(\alpha)}{N_{D}}\sigma^{2},  $$

where $N_{\theta }^{\text {eff}}$ is the effective number of parameters, which depends on the regularization penalty and regularization parameter *α*. In general, the effective number of parameters can be expressed by the second order derivatives of the objective function [96] with respect to the parameters as

(13) $${}  N_{\theta}^{\text{eff}} = \text{trace} \left(H_{\text{LS}}(H_{\text{LS}} + \alpha H_{\Gamma})^{-1} H_{\text{LS}} (H_{\text{LS}} + \alpha H_{\Gamma})^{-1} \right),  $$

where

(14) $$  H_{\text{LS}} = {\mathbb{E}}\left[{ \frac{\partial R(\theta)}{\partial \theta}^{T} \frac{\partial R(\theta)}{\partial \theta}|_{\theta = \theta_{t}}}\right]  $$

is known as the Gauss-Newton approximate of the Hessian and *H*_*Γ*_ is the Hessian of the regularization penalty function. Note that (14) is also related to the Fisher Information matrix (FIM), which is often used in the practical identifiability and uncertainty analysis of the estimated parameters [57]. For example, the eigenvalue decomposition of the FIM can identify correlated estimated parameters and parameters with high uncertainty [60]. Small or zero eigenvalues (high condition number) indicates ill-posedness, i.e. the parameter estimation problem does not have a unique solution. This eigenvalue decomposition has been widely used in the estimation literature [72, 98–102].

In the special case of ridge regularization [97], i.e. *Γ*(*θ*)=*θ*^*T*^*θ*, the Hessian of the penalty is the identity matrix and Eq. (13) simplifies to

(15) $$  N_{\theta}^{\text{eff}} =\sum_{i=1}^{N_{\theta}}\frac{{\sigma_{i}^{2}}}{(\sigma_{i} + \alpha)^{2}},  $$

where *σ*_*i*_ (*i*=1…*N*_*θ*_) are the eigenvalues of *H*_LS_. Note that for *α*=0 –the non-regularized case– the effective number of parameters equals to the number of model parameters and for *α*>0 –the regularized case– the effective number of parameters is less than the number of model parameters *N*_*θ*_. *Thus, as the regularization parameter increases, the effective number of parameters decreases and therefore the variance term of the prediction error* (10) *decreases*.

**The bias term** We saw above that regularization reduces the effective number of parameters, and therefore the variance of the prediction error. The cost to pay is the bias. Sjöberg and Ljung [97] derived an upper bound on the prediction bias for the non-weighted Tikhonov regularization, i.e. when the penalty *Γ*=(*θ*−*θ*^ref^)^*T*^(*θ*−*θ*^ref^), where *θ*^ref^ is a reference parameter vector. It was shown that in this particular case the bias is

(16) $$  \text{Bias}^{2} < \frac{\alpha}{8}||\theta_{t} - \theta^{\text{ref}}||^{2}  $$

where *θ*_*t*_ is the true model parameters. *Thus, the smaller the regularization parameter and the better our a priori knowledge is (expressed by the reference parameter vector), the smaller the bias that will be introduced in the estimation*.

**The minimal prediction error** There is a trade-off between bias and variance. From Eqs. (10), (11) (12) and (16) one obtains that the reduced variance due to the regularization is larger than the introduced bias if the following inequality holds:

(17) $$  \sigma^{2}\frac{N_{\theta} - N^{\text{eff}}_{\theta}(\alpha)}{N_{D}} > \frac{\alpha}{8} ||\theta_{t} - \theta^{\text{ref}}||^{2}.  $$

Therefore, *regularization generally increases the performance of the calibrated model when*

1. the calibration data is noisy (*σ* is large) and the amount of data is limited (*N*_*D*_ is small),
2. there are a large number of correlated parameters, and therefore the Hessian of the original problem has very small eigenvalues. In this case even a small regularization parameter can largely reduce the effective number of parameters, i.e. *N*_*θ*_≫*Nθ*eff(*α*).
3. One has a good guess of the true parameters (||*θ*_*t*_−*θ*^ref^||^2^ is small), for example from other independent experiments, previous studies or based on the biological or physico-chemical meaning of the parameters.

However, note that regularization may damage the prediction (the reduced variance is smaller than the introduced biased) if the original problem is not ill-posed, i.e. *N*_*θ*_≈*Nθ*eff(*α*), *α* is set to a large value and the provided reference parameters are far from the true parameters.

### Connection with Bayesian parameter estimation

The considered parameter estimation problem (6) follows the so-called frequentist approach. In contrast to the Bayesian approach, where the model parameters are considered random variables, in the frequentist approach the model parameters are assumed to be constants, i.e. we assume the existence of a true parameter vector *θ*_*t*_ which would predict the measurement error-free data. Yet, the *parameter estimates* are uncertain quantities following well defined distributions (depending on the measurement error and cost function), which can be calculated based on the available data.

Despite of the above fundamental difference, the formulation of the Bayesian approach can coincide with the regularized parameter estimation if some further assumptions hold. Both the considered regularization method and the Bayesian estimation approach use a priori knowledge in the parameter estimation. By noticing the similarities and differences of the two approaches we can gain further insight on how to choose the regularization parameter [87].

From the Bayesian perspective, when the estimated parameters have the *prior* distribution $\theta _{\text {prior}} \sim \mathcal {N}(\theta _{m},\eta)$, i.e. the parameters are normally distributed with mean value $\theta _{m}\in \mathbb {R}^{N_{\theta }}$ and covariance matrix $\eta \in \mathbb {R}^{N_{\theta }\times N_{\theta }}$, then the *maximum likelihood posteriori* estimate of the parameters is obtained by solving

(18) $${} \begin{aligned}  \hat \theta_{\text{Bayes}}\leftarrow\underset{\theta}{\text{minimize }} && ~ \!\!\frac{1}{2}R(\theta)^{T}R(\theta)&+\frac{1}{2} (\theta-\theta_{m})^{T}\eta^{-1}(\theta-\theta_{m})\\ \text{subject to} && ~\theta_{\text{min}} \leq & \theta \leq \theta_{\text{max}}\\ && \frac{dx(t,\theta)}{dt} &= f(u(t),x(t,\theta),\theta),\\ && y(x,\theta) &= g(x(t,\theta),\theta),\\ && x(t_{0}) &= x_{0}(\theta), \quad t \in [t_{0},\,t_{f}] \enspace. \end{aligned}  $$

Note the similarities between Eqs. (18) and (7)–(8). The regularized cost function is equivalent to the Bayesian cost function if the regularization parameters are fixed as *αW*^*T*^*W*=*η*^−1^, further, the reference parameter vector in the regularized estimation plays the role of the mean value of the prior distribution of the parameters in the Bayesian formalism (*θ*^ref^=*θ*_*m*_). Thus, the Bayesian maximum likelihood posteriori estimate can be seen as a special case of the regularization.

### Tuning the regularization

The regularization parameter balances the a priori knowledge and the information of the data, therefore plays a vital role in the regularization. When *α*=0, the regularized optimization (7) becomes the original problem (6) and the variance of the estimated parameters dominates the prediction error (10). While as *α*→*∞* the problem is well posed, but biased towards the reference parameter set. The goal of a tuning method is to find an optimal value for *α*, which minimizes the prediction (or parameter estimation) error (10).

The exact computation of the optimal regularization parameter is not possible, since the computation of the prediction bias-variance trade-off would require the knowledge of the true parameters. Many tuning methods (see [80, 85, 103, 104] and the references therein) have been developed based on different assumptions and approximations to compute an approximate regularization parameter value.

In general, in order to find the optimal regularization parameter, *α* is discretized as *α*_1_>*α*_2_>⋯>*α*_*I*_ and then the search for optimal regularization parameter is reduced to choose the best regularization parameter in this set (called the tuning of the regularization parameter). The optimization problem (7) has to be solved for each candidate, which results in the *regularization candidates*: $\hat \theta _{\alpha _{1}}$, $\hat \theta _{\alpha _{2}}$,…$\hat \theta _{\alpha _{I}}$. This is a computationally expensive task, although in an iterative framework the previously obtained solutions can be utilized to reduce the computational cost of the remaining candidates.

Regularization tuning methods can be classified based on the type of information they require (error level in the data, limits for the regularization parameter, further tuning parameters) and in the way the optimal regularization parameter is selected among the candidates. In Table 1 we shortly summarize the regularization tuning methods that we have considered and compared. Further details about each tuning method can be found in Additional file 2. The methods considered can be classified into the following five groups:

- Discrepancy principle (DP) is based on the idea that the regularization parameter should be chosen such that the sum of residuals should be equal to the error level of the data. For that, a good estimate of the measurement error is needed, which is often not known. Other versions of the discrepancy principle, such as the modified discrepancy principle (MDP) and the transformed discrepancy principle (TDP) are known to be less sensitive to the accuracy of a priori error level.

**Table 1:** Overview of the regularization tuning methods considered. We have indicated with a *✓* sign for each method, (i) which data/information is required (residual vector, estimated kinetic model parameters or the Jacobian of the residual vector), and (ii) whether the regularization method utilizes further tuning parameters, an estimate of the measurement noise level or a limit for the maximal/minimal regularization parameter. Finally, the last three columns indicate if a computationally expensive procedure is involved, which can be an issue for large scale problems. SVD denotes singular value decomposition

Regularization method			Computation involves			Further required inputs			Involved computation		
Method	Short ID	Refs	Residuals	Estimated	Jacobian	Tuning	Meas. error	*α*_max_/*α*_min_	Matrix	SVD	Trace
				parameters		parameter	estimate		inverse		
Discrepancy principle	DP	[113]	*✓*	-	-	*✓*	*✓*	-	-	-	-
Modified DP	MDP	[114]	*✓*	-	*✓*	*✓*	*✓*	-	*✓*	-	-
Transformed DP	TDP	[115]	*✓*	-	*✓*	*✓*	*✓*	-	*✓*	-	-
Monotone Error Rule	MER	[116]	*✓*	*✓*	*✓*	*✓*	*✓*	-	*✓*	-	-
Balancing Principle	BP	[117]	-	*✓*	*✓*	*✓*	*✓*	-	-	*✓*	-
Hardened Balancing	HBP	[118]	-	*✓*	*✓*	-	-	-	-	*✓*	-
Quasi optimality	QO	[80]	-	*✓*	-	-	-	*✓*	-	-	-
L–curve method (curvature)	LCC	[105]	*✓*	*✓*	-	-	-	*✓*	-	-	-
L–curve method (Reginska)	LCR	[119]	*✓*	*✓*	-	-	-	*✓*	-	-	-
Extrapolated Error Rule	EER	[120]	*✓*	-	*✓*	-	-	-	-	-	-
Residual Method	RM	[121]	*✓*	-	*✓*	-	-	*✓*	*✓*	-	*✓*
Generalized Cross-validation	GCV	[122]	*✓*	-	*✓*	-	-	-	*✓*	-	*✓*
GCV (Golub)	GCVG	[107]	*✓*	-	*✓*	-	-	-	*✓*	-	*✓*
Robust GCV	RGCV	[108]	*✓*	-	*✓*	*✓*	-	-	*✓*	-	*✓*
Strong RGCV	SRGCV	[109]	*✓*	-	*✓*	*✓*	-	-	*✓*	-	*✓*
- Monotone error rule (MER) and quasi optimality criteria (QO): they use the observation that the differences between successive candidates, i.e. $||\hat \theta _{\alpha _{i}} - \hat \theta _{\alpha _{i+1}}||$, are large due to either large regularization or large propagated error and the difference becomes small for the optimal regularization parameter.
- Balancing (BP) and hardened balancing principle (HBP): they use all the candidates to estimate the regularization error, which is compared then to the so called approximated propagated error bound. The optimal regularization parameter is for which the two types of estimated error is minimal.
- L-curve method: as proposed by Hansen et al. [105] to display information about the candidates $\hat \theta _{\alpha _{i}}$, *i*=1…*I*. By plotting the two parts of the objective function (7): the model fit $Q_{\text {LS}}(\hat \theta _{\alpha _{i}})$ and the regularization penalty $\Gamma (\hat \theta _{\alpha _{i}})$ for {*α*_1_,…*α*_*I*_} one obtains a discrete Pareto optimal front, which usually has an L-shape (see for example in Fig. 5a). The horizontal part is formed by the solutions corresponding to large regularization parameters, where the regularization bias is dominating. As the regularization parameter decreases the least squares error reaches a limit that is determined by the measurement noise and the model flexibility. On the vertical part of the L-curve a small reduction in the least squares model fit error usually cause a large increase in the penalty. Intuitively, the optimal regularization parameter that balances the two types of error is located near the corner of the L-shaped curve. In [106] the corner point is defined as being the point that has the largest curvature on the L-curve (LCC).
- Generalized cross validation (GCV): an approach by Golub [107] that aims to find the regularization parameter that minimizes the leave one out (LOO) prediction error [63]. It does not require any estimate of the measurement error, but it can be sensitive if a small number of measurement data is at hand. For this reason, other variants, such as the robust (RGCV) and the strong robust generalized cross validation methods [108, 109] (SRGCV) have been developed.

As we will see from the results in Section “Tuning the regularization and prior knowledge”, the generalized cross-validation method was found to be the best for the presented regularization procedures.

### Implementation details

**Fig. 1:**
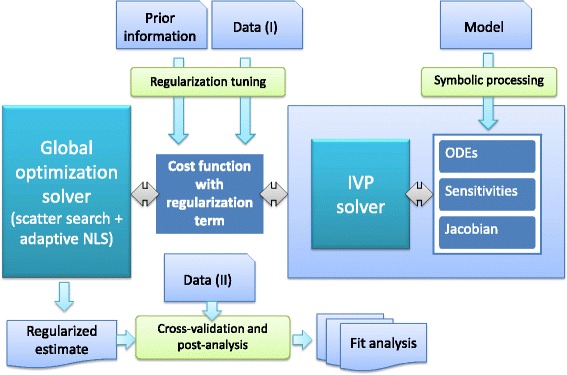
Architecture of the method: in the pre-processing phase, sensitivity equations and Jacobians (both of the residuals and of the differential equations) are derived via symbolic manipulation, generating C code which is then linked to the initial value problem (IVP) solver, CVODES. The regularization scheme is selected according to the quality of the prior knowledge, and tuned following the procedure described in section “Tuning the regularization and prior knowledge”. Finally, global optimization with eSS2 is used to find the regularized estimate of the parameters. The resulting calibrated model can then be further evaluated using cross-validation, followed by additional post-regression and goodness-of-fit analysis

In Fig. 1 we outline the architecture of the resulting method and its implementation. The pre-processing step makes use of symbolic manipulation to derive the sensitivity equations and Jacobians (both of residuals and dynamics), and automatically generates C code to be compiled and linked with the CVODES solver. This procedure ensures the highest numerical efficiency and stability during the solution of the initial value problem. This is especially useful in stiff nonlinear dynamic systems, where wild dynamic behaviour can occur during the exploration of the parameter space.

The regularization scheme is selected according to the quality of the prior knowledge (as described in Section “Regularization schemes based on available information” and illustrated in Fig. S.2.1 in Additional file 1), and the cost function is formulated. The regularization is then tuned following the procedure described in Section “Tuning the regularization and prior knowledge”. Finally, global optimization with eSS2 is used to find the regularized estimate of the parameters. The resulting calibrated model can then be further evaluated using cross-validation, followed by additional post-regression and goodness-of-fit analysis.

## Results and discussion

### Numerical case studies

We have considered a set of seven parameter estimation problems, which are used as numerical case studies. These case studies have been chosen as representatives of the typical problems arising in computational systems biology, i.e. partially observed nonlinear dynamic models and sparse noisy measurements. These examples include signalling and metabolic pathway models of increasing complexity. Table 2 contains a short summary of these case studies, with the original references and an overall view of number of estimated parameters, dynamic state variables, observables, and data. Further details, including model equations and the data sets used for model calibration and cross-validation are reported in Additional files 3 and 4, respectively. It should be noted that in several of these examples the original references only describe the model dynamics, not the full parameter estimation problems.

**Table 2:** Summary of the case studies. Each column describes a calibration problem. Further details can be found in Additional file 3 (numerical data) and Additional file 4 (detailed descriptions including the model differential equations)

Short name	BBG	FHN	MAPK	GOsc	TGFB	TSMP	CHM
Description	Biomass	FitzHugh-	MAPK	Goodwin’s	TGF- *β*	3-Step	Chemotaxis
	Batch	Nagumo	Signalling	Oscillator	Signalling	Metabolic	Signalling
	Growth	Oscillator	Pathway		Pathway	Pathway	Pathway
Reference	[123]	[124, 125]	[126]	[127]	[48]	[44]	[128]
Implementation of dynamics	[123]	BIOMD00000000010 ^∗^	BIOMD00000000346 ^∗^, [129]	[130]	[48]	[44]	BIOMD00000000404 ^∗^
Total parameters	4	3	22	8	21	36	60
Estimated parameters	4	3	6	8	18	36	38
States	2	2	8	3	18	8	26
Observed states	2	1	2	3	16	8	7(+1)
Experiments	1	1	1	1	1	8	2
Data points	22	6	20	20	240	1344	160

^*^The dynamic model can be found in the Biomodels Database (http://www.ebi.ac.uk/biomodels-main)

In the following sections, we use these examples to illustrate the issues and pitfalls arising from the nonconvexity and ill-conditioning of the estimation problems. Next, we use them to illustrate the key ideas behind the methods presented above, including the bias-variance trade-off, the tuning of the regularization, the effect of the quality of the prior knowledge on the regularization, and their impact on cross-validation results. For the sake of brevity, we include summarized or selected results in the main text, but detailed results for all the case studies can be found in Additional file 5.

### Multi-modality of the optimization problem

**Fig. 2:**
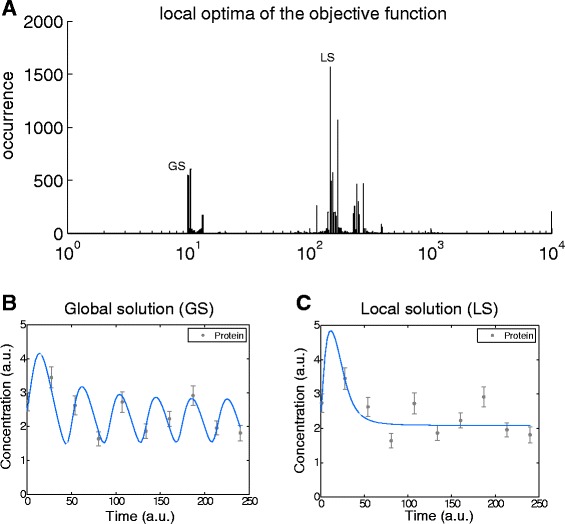
Local optima of the objective function corresponding to the Goodwin’s oscillator case study (GOsc). Figure **a** shows the distribution of the final objective function values of 10,000 runs of local solver NL2SOL from randomly chosen initial points based on Latin hypercube sampling. The distribution of the local optima shows that only 6 % of the runs finished in the close vicinity of the global optima (minimum objective function value: 9.8903). Figure **b** shows the fit corresponding to the global optima (global solution – GS). Figure **c** depicts the fit corresponding to the most frequently achieved local minima (local solution – LS, objective function value: 148.25). Note the qualitatively wrong behaviour of this fit, i.e. the lack of oscillations in the predictions

Since the estimation problem stated above is nonconvex, multi-modality (existence of multiple local solutions) will be a key possible pitfall. As already discussed, local nonlinear least squares (NLS) algorithms will find the local minima of the objective function in the vicinity of the initial point. A characterization of the set of possible local optima can be obtained by the frequency distributions of the solutions found by a multi-start local procedure, i.e. starting local optimizations from different initial points, selected randomly in the parameter space. If the initial points cover the parameter space adequately well, the observed distribution of the local optima can be used to quantify the difficulty of the parameter estimation problem arising from multi-modality. For example, Fig. 2a shows the distribution of these local minima for the Goodwin’s oscillator (GOsc) case study. The distribution was obtained by solving 10,000 optimization problem (of which approximately 97 % converged) with the NL2SOL NLS algorithm started from randomly chosen initial guesses. These initial points were selected based on the logarithmic Latin hypercube sampling (LHS) method (see Additional file 1). The distribution of the obtained local optima is spread along several magnitudes (note the logarithmic scaling on the x-axis), with the best (lowest) objective function value of 9.8903, which is very close to the best known solution for this problem and therefore can be considered as global minimum of the objective function. Although the local optimization was enhanced by high quality Jacobian information based on the sensitivity calculations, only 5 % of the runs achieved the vicinity of the global optima.

The calibration data and the simulation results of the most frequently occurring local optima (marked as LS in the histogram; objective function value: 148.25) is shown in Fig. 2c. This is certainly a potential pitfall of using local optimization, which can lead to wrong conclusions about the model predictive capability. In contrast, the fit of the global solution (marked as GS in the histogram) is depicted in Fig. 2b, showing a good agreement between the model and the data.

**Fig. 3:**
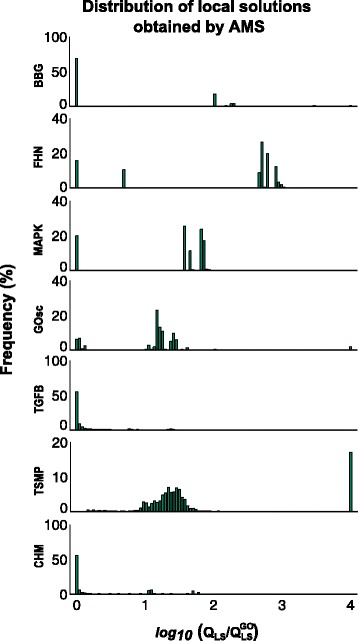
Distributions of local optima for all case studies. Each case study was solved by the AMS method and the observed frequency of the local minima is reported here. Note that the objective function values (*Q*_LS_) are scaled by the global optimum *Q*LSGO for each case study, and the resulting ratio is reported in logarithmic scale. The height of the first bin at 0 represents the frequency of finding the vicinity of the global solution

We applied the same procedure to the other case studies, with the corresponding histograms shown in Fig. 3. These histograms show that all these case studies exhibit multi-modality, but in different degree. We can see that oscillators tend to exhibit more local minima than the other types. However, case study TSMP, which does not exhibit oscillations, presents a particularly challenging histogram: none of the local searches was able to locate the vicinity of the global solution. In summary, some of these problems could in principle be solved by a multi-start local method, especially if using high quality gradients. But this approach would fail in other cases, and we have no a priori way of distinguishing between these two groups. Therefore, we conclude that an efficient global optimization approach should be used in all cases to avoid artifices (local solutions) and ensure the best possible fit.

### Convergence of the optimization algorithms

Once we have characterized the multi-modality of the case studies, we now illustrate the advantages of using the eSS2 global optimization method presented previously. First we consider the solutions of the non-regularized calibration problems (6), and then in the following subsection we will discuss the regularized estimations (7). The metric to be used will be based on the convergence curves, i.e. cost function values versus computation time. In order to evaluate the improvements in efficiency and robustness, we will compare the following methods for all the case studies, using a fair stopping criteria based on when a predefined computational time budget is reached:

- simple multi-start (SMS) of NL2SOL with finite difference Jacobian computation.
- advanced multi-start (AMS), similar to SMS, but the bounds of the feasible range of the parameters are transformed by the logarithmic function and then the Latin hypercube sampling method is utilized to sample initial points (see Additional file 1). This way the parameter space is better sampled, especially if the upper and lower bounds of some parameters have very different magnitudes (which is the case for all case studies). Further, NL2SOL is provided with high quality Jacobian of the residual vector.
- : the new enhanced scatter search described above, making use of NL2SOL and high quality Jacobian.
- : like eSS2a but initialized by the log Latin hypercube sampling as in AMS.

**Fig. 4:**
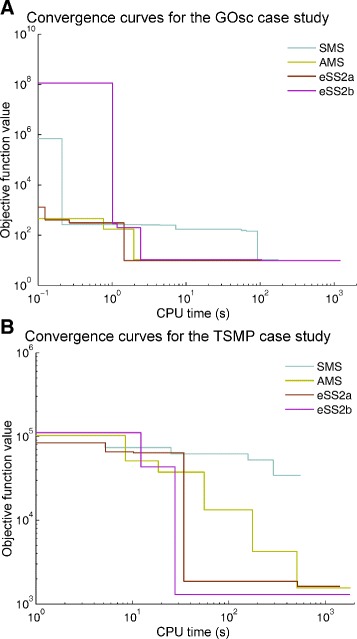
Comparison of convergence curves of selected optimization methods. The convergence curve shows the value of the objective function versus the computation time during the minimization (model calibration). Results are given for simple multi- start (SMS), advanced multi-start (AMS) and enhanced scatter search methods (eSS2a and eSS2b; see description in main text). Results are shown for two case studies: (**a**) GOsc and (**b**) TSMP

The above methods are compared based on their convergence curves (see for example Fig. 4) and the distribution of the final cost function values reached (reported in Additional file 5). The empirical convergence curve depicts the current best objective function value as the optimization algorithm proceeds. An optimization method performs better than another method if a lower objective function value is reached in the same amount of computation time. Since both the multi-start and the eSS2 approaches use random numbers, the convergence curves will be different for each run. Thus we need to compare the convergence curves for several runs of each method.

Figure 4 shows the convergence curves for the Goodwins’ oscillator case study (GOsc) and for the 3-step metabolic pathway problem (TSMP). For each method the optimization was carried out 20 times using different seeds for the random number generator, but here only the best convergence curve is shown, i.e. the run in which the best solution was reached in the shortest time by each method. Detailed results of the 20 runs can be found in Additional file 5 for all case studies. Clearly, the simple multi-start (SMS) approach performed poorly in both cases: in GOsc, SMS needed 50 times more computation time than eSS2 to achieve the vicinity of global minimum, while in TSMP it could not find it in the given computation time budget. The advanced multi-start (AMS) presented a performance similar to eSS2a and eSS2b for the GOsc case study, but in TSMP it was clearly outperformed by eSS2b.

Considering the results for all the case studies (see detailed convergence curves in Additional file 5), we can conclude that the more refined version of multi-start can solve problems of small size (number of parameters) and with relatively tight bounds and good initial guesses for the parameters, but it is not reliable in more difficult situations. In contrast, the eSS2b method performed consistently well, solving all the problems in reasonable computation time using its default options (i.e. without the need of tweaking the method’s search options with preliminary runs). In the remaining text we will refer to eSS2b as eSS2.

**The effect of regularization on the convergence**

We now consider how the penalty regularization (7), which changes the topology of the objective function, affects the convergence of the optimizer. We used eSS2 to solve the regularized problem for each case study, finding a narrower spread of the convergence curves. We also found improvements in the average time to reach the global solution. This benefit was especially clear in the TSMP case study, where the robustness was greatly improved (all the 20 runs of the optimization with regularization reached the global optima in 200 seconds of computation time, while only 3 runs converged using the same algorithm with the non-regularized objective function). Detailed results for all case studies are reported in Additional file 5.

This additional beneficial effect of regularization on the convergence can be explained as follows: while the original cost function is multi-modal, the penalty term in Tikhonov regularization (8) is a quadratic (convex) function. Thus, in the limit *α*→*∞* the regularized objective function becomes a convex function.

Note that, the global minimum of the objective function is always larger for the regularized problem ($Q_{\mathrm {R}}(\hat \theta _{\alpha })$ in (7)) than the value for the non-regularized problem ($Q_{\text {LS}}(\hat \theta)$ in (6)). This is because the penalty term ($\alpha \Gamma (\hat \theta _{\alpha })$) contributes only to the objective function in (7). Further, the regularization avoids overfitting the data, thus the sum of squared residuals part of the objective function ($Q_{\text {LS}}(\hat \theta _{\alpha })$), is also larger than the minimum of the non-regularized solution ($Q_{\text {LS}}(\hat \theta)$).

### Tuning the regularization and prior knowledge

Kinetic parameters of bio-models are generally unknown and vary for different cells. Thus, even if we have some prior knowledge about the parameters, it should be tested against the data. As shown later in section “Ill-conditioning, cross-validation and overfitting”, the predictions of the calibrated models using good prior knowledge in the regularization agree with the cross-validation data and thus generalize better.

In order to adjust the right level of the regularization, the regularization parameter (*α*) has to be tuned. The tuning includes three steps (TS):

- **TS1:***a set of regularization parameter candidates are determined*: *α*_1_, *α*_2_, …*α*_*I*_. To cover large range with few elements, typically the candidates are determined as the elements of a geometric series, i.e. *α*_*n*_=*α*_0_·*q*^*n*^ for *n*=1…*I*, where *α*_0_>0 and 0<*q*<1.
- **TS2:***the regularized calibration problem* (7)-(8) *is solved* for each regularization parameter. This results in a set of calibrated models (candidate models), with estimated parameters denoted by $\hat \theta _{\alpha _{1}}$, $\hat \theta _{\alpha _{2}}$ …, $\hat \theta _{\alpha _{I}}$.
- **TS3:***the best candidate is selected* based on a tuning method:
  $\{\hat \theta _{\alpha _{1}},\,\hat \theta _{\alpha _{2}},\, \dots,\,\hat \theta _{\alpha _{I}}\} \to \hat \theta _{\alpha _{\textit {opt}}}$

In TS1, the range (10^−3^−10^3^) with *I*=11 candidates was found to be a good balance between accuracy and computational cost for all the case studies considered. In TS2, the calibration problems with different candidates can be solved parallel, since they are essentially independent optimization problems. However, when solved sequentially, the previously obtained solutions can be used to start the next optimization problem from a good initial point, and thus reduce its computational cost. We report further practical considerations in Additional file 1.

**Fig. 5:**
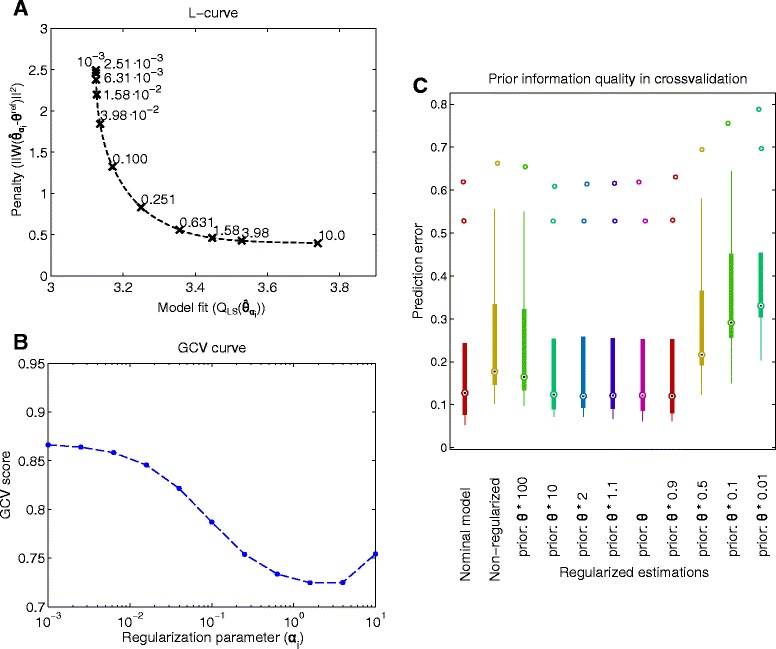
Tuning the regularization method for BBG case study. Figure **a** shows the trade-off between the two terms of the regularized objective function, i.e. model fit and the regularization penalty, for a set of regularization parameters (values shown close to symbols). A larger regularization parameter results in worse fit to the calibration data, small regularization parameter results in a larger penalty. Figure **b** compares the candidates based on the generalized cross-validation scores. A larger score indicates worse model prediction for cross-validation data. The curve has the minimum at 1.58. Figure **c** shows the normalized root mean square prediction error of calibrated model for 10 sets of cross-validation data and regularizations considering different quality of the prior information (initial guess of the parameters). For a wide range of priors (initial guesses based on the reference parameter vector) the regularized estimation gives a good cross-validation error. Small priors exhibit worse predictions

Figure 5a depicts trade-off between the model fit and regularization penalty for the candidates in the biomass batch growth (BBG) case study. Each cross in the figure corresponds to a calibrated model with the regularization parameter denoted by the labels next to the crosses. Larger regularization parameter results in an estimated parameter vector closer to the reference parameter vector and therefore smaller penalty, but worse fit to the calibration data.

The best way to select the optimal candidate in TS3 is cross-validation [110], but it requires an independent set of data at the time of calibration. However, in general it is unclear how the total amount of data should be divided [111] into a calibration and validation set for regularization. In case of scarce data, where the splitting is undesirable, tuning methods must be used.

We have tested 15 tuning methods on the case studies by comparing the regularization parameter selected by each tuning method with the optimal regularization parameter which minimizes the prediction error (i.e. the one with the best bias-variance trade-off). The optimal regularization parameter and the regularization parameters selected by the tuning methods are reported in Additional file 2 for each case study. We found the (robust) generalized cross validation method as the most reliable, since it identified the optimal regularization parameter reliably, outperforming the other methods.

The generalized cross-validation method does not use any further cross-validation data, but estimates the leave-one-out cross validation error of the candidate models based on the calibration data. The criteria is computed as

(19) $$ \text{GCV}(\alpha_{i}) = \frac{\text{RSS}(\alpha_{i})}{N_{\mathrm{D}} - N_{\theta}^{\text{eff}}(\alpha_{i})}, ~~ \text{for}\, i = 1,\,\dots I  $$

where RSS(*α*_*i*_) is the sum of squared normalized residuals for the candidate ($\text {RSS}(\alpha _{i}) = R(\hat \theta _{\alpha _{i}})^{T} R(\hat \theta _{\alpha _{i}})$), *N*_*D*_ is the number of calibration data and $N_{\theta }^{\text {eff}}(\alpha)$ is the effective number of fitted parameters in the model calibration (15). The RSS(*α*) grows with *α* since larger regularization results in a worse fit to the data (see Fig. 5a). The larger the *α* is, the more the fitted parameters are constrained by the reference parameter vector, thus the effective number of fitted parameters decreases with *α* (see Eq. (15)). The generalized cross validation error is small if the model fits the data well, while it also has a low number of effective parameters. Figure 5b shows the computed GCV value for the candidates in the BBG case study. It shows a minimum for the regularization parameter 1.58. Note that in cases where the amount calibration data is small, the GCV method tends to under-regularize the calibration [108], so the robust GCV (RGCV) method was found to be a better alternative.

The quality of the regularized calibration depends not only on the regularization parameter, but also on the prior knowledge of the modeller encoded by the reference parameter vector *θ*^ref^ and scaling matrix *W*. To test the robustness of the method with respect to these input information, we chose a range of reference parameter vectors and scaling matrices and solved the regularized optimization problem for each case study. In each case the generalized cross-validation score was used to select the regularization parameter. Then, the calibrated models were tested by computing predictions for cross-validation data sets. Figure 5c depicts the results for the BBG case study using box-plots. The first two columns show the distribution of the prediction error (normalized root mean square error) for the nominal model (known only in synthetic problems and used only for reference) and for the model calibrated without regularization. The next 9 columns in the plot show the prediction error with different quality of prior knowledge. We can see that the regularization method gives better predictions than the non-regularized for a wide range of prior quality.

### Prediction and parameter bias-variance trade-off

Here we consider the stability of the solution of the optimization problem with respect to small perturbation in the data. Note that this numerical analysis is partially based on the bias-variance decomposition of the estimated model predictions and estimated parameters, thus it requires the knowledge of the nominal (true) parameter vector. Obviously the true model is known only for synthetic problems, but it can be used as a way to analyse the reliability of computational methods.

The experimental data is always measured with some uncertainty, which also influences the model calibration. If we could repeat the experiments, for example 10 times, taking measurements in the same conditions, we could collect 10 different datasets with slightly varying measurements –due to the random measurement error. Then each of the 10 datasets could be used to calibrate a model with and without regularization, which would result 10 slightly different calibrated models for both the non-regularized and regularized calibration procedure. Analysing the consistency of these models can reveal the sensitivity of the calibration procedure to the measurement error.

**Fig. 6:**
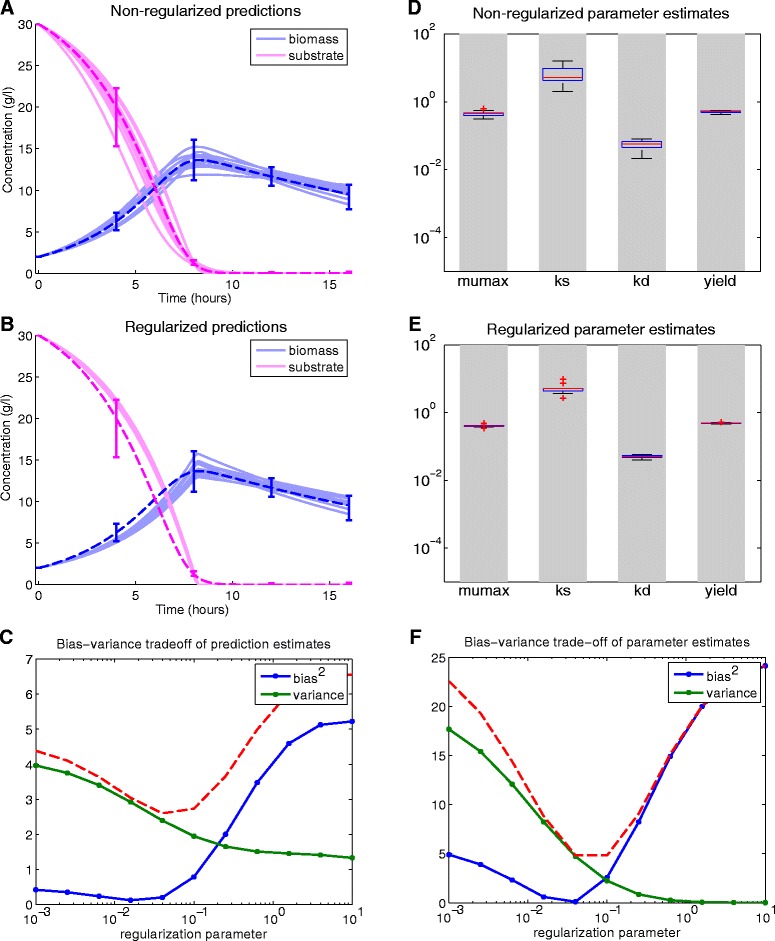
Bias-variance trade-off for the BBG case study. Figures **a** and **b** illustrate the nominal trajectory (dashed line) and the range of perturbed measurements together with predictions of calibrated models (continuous lines) without and with regularization, respectively. The distribution of the regularized predictions (in **b**) are narrower than in the non- regularized one (in **a**), but are slightly biased from the nominal trajectory. Figure **c** depicts the squared bias and the variance of these model predictions as a function of the regularization parameter. The mean square error (dashed line) has a minimum at 0.08. Figure **d**, **e** and **f** shows the results for the estimated parameters: with regularization the estimated parameters are less sensitive to perturbations in the data

The results of this procedure for the BBG numerical case study can be seen in Fig. 6a and 6b, where the nominal model predictions are shown by dashed line together with the range of the measured data depicted by error bars. In Fig. 6a the predictions of the models, calibrated in the traditional way –without the regularization– is also shown, in contrast, the models shown in Fig. 6b were calibrated using regularization. We can observe that the model predictions are less sensitive to the error in the data when regularization is applied, i.e. the variance of the model predictions are smaller. However, we also observe larger bias from the nominal trajectory for the regularized models, since no prior knowledge was used in this case (worst case scenario).

Figure 6c shows the prediction bias-variance trade-off for a range of the regularization parameter (see computational details in Additional file 1). The results are in agreement with the intuition that a lower regularization results in larger prediction variance and less bias. The mean squared error curve (the red dashed line), i.e. the sum of squared bias and variance, has the minimum for the regularization parameter $\alpha _{\text {opt}}^{\text {Pred}} \approx 0.04$, which is therefore the optimal regularization with respect to the prediction error.

Similar trends and results were obtained regarding the estimated parameters, shown in Fig. 6d and 6e. Here, the distribution of the parameter estimates in the 10 regularized and 10 non-regularized calibrations are depicted by box-plots and the grey boxes show the feasible range of the parameters. The regularized calibration results in much narrower distribution for the estimates (note the logarithmic scaling of the y-axis). The bias-variance trade-off in the estimated parameters is shown in Fig. 6f. The optimal regularization parameter for the *minimum mean squared parameter estimation error* ($\alpha _{\text {opt}}^{\mathrm {Param.}} \approx 0.04$) coincides with the previously obtained value for the *minimum mean square prediction error* in this case study. Although for all case studies we found that $\alpha _{\text {opt}}^{\text {Pred}} $ and $\alpha _{\text {opt}}^{\mathrm {Param.}}$ are close to each other, they do not necessarily coincide.

### Ill-conditioning, cross-validation and overfitting

It is a common problem that due to the large measurement error (large noise to signal ratio) and due to data scarcity, a model with different numerical parameter values might fit the data almost equally well, which indicates identifiability problems.

**Fig. 7:**
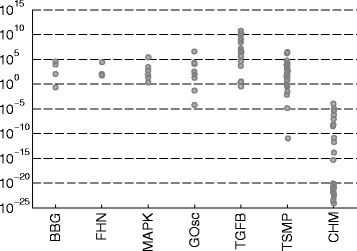
Eigenvalues of the approximated Hessian matrix for each case study. Eigenvalues are related to the identifiability of the model parameters: a large spread indicates lack of identifiability of some parameters from the given dataset

A posteriori to the calibration, local analyses of the topology of the objective function can provide valuable information about the uncertainty in the estimated parameters. Particularly, the ill-conditioning of the approximated Hessian of the objective function (Eq. (14)) evaluated at the global optima can indicate high uncertainty in the estimated parameters [60]. Figure 7 shows the eigenvalues of this matrix for each case study. We can see that larger models with more parameters tend to have larger a spread in the eigenvalues, and thus larger condition number, indicating the lack of identifiability of its parameters. However, this is only a local measure of the ill-conditioning of the problem near the optima.

A more sound way to measure the predictive value (generalizability) of the calibrated model is cross-validation, where a different set of data is used to asses the calibrated model. Over-fitted models will show a bad fit to cross-validation data since they fitted the noise, rather than the signal, and therefore are less generalizable. If the experimental conditions for collecting the cross-validation data are different from the calibration conditions –e.g. different stimuli levels, time-horizon etc.–, this effect will be more prominent.

**Fig. 8:**
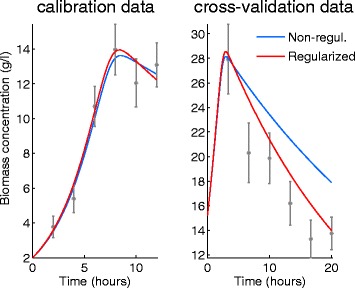
Calibration and cross-validation results for the BBG case study. Left figure shows calibration data fitted with non-regularized and regularized estimations (non-regularized, $Q_{\text {LS}}(\hat \theta) = 3.68$ and regularized $Q_{\text {LS}}(\hat \theta _{\alpha }) = 4.09$). Right figure shows cross-validation data with the predictions from the non-regularized and regularized estimations. The regularized model shows a slightly worse fit to the calibration data but much better agreement with the cross-validation data. I.e. regularization results in a more generalizable model

Figure 8 shows the calibration fit (on the left) and the cross-validation (on the right) for the BBG case study (substrate measurements are not shown). The predictions of two models, one that was calibrated in the traditional way and one that was calibrated with regularization are also presented. Although there is almost no difference between the model predictions for the calibration data, the predictions for cross-validation data are rather different. The model that was calibrated without regularization predicts a slower decrease in the biomass concentration and shows large discrepancy from the cross validation data. If we compare the least-squares cost function for the two models, we find that the non-regularized model fits better the calibration data, but the regularized model generalizes better for the cross-validation data. In other words, the traditional model calibration results in overfitting, while the regularized calibration gives a more generalizable model at the expense of a slightly worse fit to the calibration data. Ideally, the cross-validation experimental scenario should be different from the calibration one in order to better assess generalizability of a model. Typically this can be achieved generating cross-validation data with experiments where the initial and boundary conditions (e.g. stimuli) of the experiments are as different as possible from those used to obtain the calibration data.

**Fig. 9:**
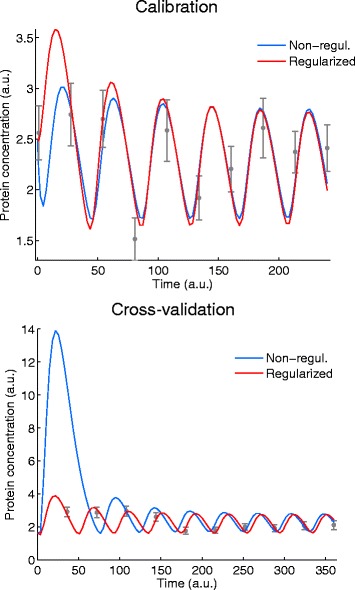
Calibration and cross-validation results for the GOsc case study. Left figure shows fits to calibration data, right figure shows agreement of predictions for cross-validation data. The non-regularized and the regularized fits show some differences in the first two oscillations, although at the measurement times the predictions are almost identical. In cross-validation, the non-regularized model shows a heavy overshoot at early times

In Fig. 9 we present similar results for the Goodwin’s oscillator case study (GOsc). Here, we already see larger differences between the model predictions in the calibration data, but note that the predictions are almost identical at the time of the measurements. Thus, for example, based on the calibration data it would be impossible to decide whether the protein concentration decreases or increases right after the beginning of the experiment. When the two models are cross-validated on an independent set of data (lower plot in Fig. 9) we see that the regularized model is in good agreement with the new data, while the non-regularized model heavily overshoots the data in the first period of the oscillation.

**Fig. 10:**
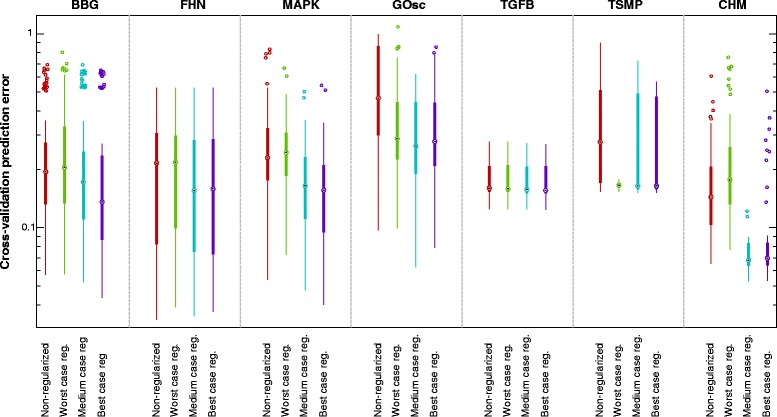
Prediction errors distribution for each case study. Prediction errors (box-plots of normalized root mean square error in log-scale) of the calibrated models with and without regularization are shown for each case study. These distributions were obtained by calibrating the models to multiple sets of calibration data (as explained in section “Ill-conditioning, cross-validation and overfitting”) and cross-validating them on multiple cross-validation data sets. Most cases show the trend that better prior knowledge results in smaller cross-validation errors, i.e. regularized models are more generalizable

Figure 10 summarizes our findings for all case studies regarding the generalizability of the calibrated models. Each case study was solved in the traditional, non-regularized way and with regularization assuming different level of prior knowledge (worst, medium and best case scenarios). Due to the low number of calibration data and large measurement noise, we found large variability of the predictions depending on the exact noise realization in the calibration data. Thus we repeated the calibrations with 10 calibration datasets to obtain robust results. Then, each calibrated model was cross-validated in 10 independent cross-validation data sets and the prediction error was computed. Figure 10 shows the distribution of these prediction errors for each case study by box-plots.

The distributions can be compared by the observed medians, which are denoted by the black dots in the box-plot. In order to check if the observed differences in the medians are significant we utilized the Wilcoxon non-parametric statistical test [112] (also known as the Mann-Whitney U test). The test results show that in the majority of the scenarios the differences in the medians are statistically significant at the 0.05 level. The exception is the FHN case study where the differences turned out to be not significant for the three scenarios. Further details of this statistical test are reported in Table S4.9.1 in Additional file 5.

By comparing the medians of the distributions we see that in almost all cases the non-regularized models overfit the calibration data, i.e. the non-regularized models fit well the calibration data, but do not predict cross-validation data as well as the regularized models. In each case, the medium and the best case regularization scenarios clearly outperformed the non-regularized estimation, leading to better generalizable calibrated models. However, in two cases we observe that the worst case regularization scenario performed worse than the non-regularized case. Also note, that in case of the TGF- *β* pathway problem (TGFB) all scenarios gave almost identical results, meaning that the original problem is a well-posed calibration problem. However, this is generally unknown before the calibration.

In this context, it is worth mentioning that the regularization of non-mechanistic (e.g. data-driven) models –like those used in machine learning and system identification, such as e.g. neural networks– usually exhibits more dramatic benefits. The reason is that these data-driven models are by definition extremely flexible and therefore very prone to overfitting. In the case of the mechanistic kinetic models used in systems biology, in many cases they will have a rather rigid structure despite being over-parametrized. Therefore, they might be less prone to overfitting. However, a clear exception are models exhibiting oscillatory behaviour, or models with many non-observable states.

### Regularization schemes based on available information

Based on the above results, we recommend the following regularization procedures for the three scenarios defined previously (in Section “Scenarios based on prior information”):

- **IBest case:** a good guess of the parameter values (*θ*^guess^) is available. In this case a first order weighted Tikhonov regularization is recommended, i.e. *θ*^ref^:=*θ*^guess^ and the weighting matrix should be initialized by the parameters too, i.e. *W*=diag(1./*θ*^ref^), where./ is the element-wise division. In this way, parameters with different magnitudes will contribute similarly to the penalty. In section “Connection with Bayesian parameter estimation” a similar concept about the weighting matrix was shown from the Bayesian perspective.
- **IIMedium case:** a situation where a less reliable initial guess –but within one order of magnitude of the true values– is available. As in the best case scenario, the parameter guess should be used as the reference vector in the regularization penalty: *θ*^ref^:=*θ*^guess^. However, we found, that including these values also in the weighting matrix amplified the error in the parameter estimate. Therefore, the non-weighted Tikhonov regularization is recommended.
- **IIIWorst case:** no prior knowledge and therefore only a random guess of parameters is available. Here a two-step regularization procedure is proposed. In the first step ridge regularization is applied which results in a ridge estimate, denoted by $\hat \theta _{\alpha }^{\mathrm {R1}}$. In the second step this parameter vector is used as the reference parameter vector for Tikhonov regularization, i.e. ($\theta ^{\text {ref}}:= \hat \theta _{\alpha }^{\mathrm {R1}}$). This procedure could be repeated *n*-times –using the obtained regularized solution as reference parameter vector in the next step–, resulting in the *n*-th order Tikhonov regularization [80], but we found no practical difference after the second step.

The regularized optimization is solved for a set of regularization parameters in each scenario and depending on the amount of data at hand the generalized cross validation method (GCV) – for larger dataset– or the robust generalized cross-validation method (RGCV) – for smaller dataset– is recommended to choose the optimal candidate. A summary of this regularization scheme is illustrated in Fig. S.2.1 in Additional file 1.

Based on the results presented previously, we suggest that tuning of the regularization can be avoided in certain situations, saving considerable computation time. For scaled models where the number of data points and parameters are similar and the data has 5–10 % measurement error, our study indicates that the optimal regularization parameter will lie in the range [0.1−10]. For the worst case scenario, rather common in systems biology, we found that the above procedure gave smaller mean square parameter estimation error than the traditional, non-regularized estimation. Further, the optimization algorithm exhibited better convergence properties with regularization, although no significant improvements in the model predictions was observed. In the case of medium and best scenarios *regularized estimation led to both better parameter estimates and smaller cross-validation prediction error in shorter computation times*.

## Conclusions

In this study we propose a new parameter estimation strategy for nonlinear dynamical models of biological systems. This strategy is especially designed to surmount the challenges arising from the non-convexity and ill-conditioning that most of these problems exhibit. The difficulties of parameter estimation problems in systems biology do not only depend on the number of parameters, but also on the structure (flexibility and nonlinearity) of the dynamic model, and the amount of information provided by the (usually scarce and noisy) available data.

Our strategy combines an efficient global optimization method with three different schemes of Tikhonov regularization, selected depending on the quality of the prior knowledge. We tested this strategy with a set of case studies of increasing complexity. The results clearly indicate that an efficient global optimization approach should always be used, even for small models, to avoid convergence to local minima. Similarly, our study illustrates how ill-conditioning and overfitting issues can damage the generalizability of the calibrated models. Overfitting was found to be especially important when models are flexible (e.g. oscillatory models), even if the number of parameters is small. Our results show how regularization can be used to avoid overfitting, leading to calibrated models with better generalizability. Finally, the use of regularization significantly improved the performance of the optimization method, resulting in faster and more stable convergence.

## Additional files

Additional file 1
**Detailed description of methods.** This file contains detailed descriptions of the methods and associated computational details. (PDF 251 kb)

Additional file 2
**Regularization tuning methods.** This file contains further details and results about the regularization tuning methods. (PDF 300 kb)

Additional file 3
**Numerical data.** This file is a spreadsheet containing the numerical data used for the models calibration and cross-validation. (ODS 891 kb)

Additional file 4
**Numerical case studies.** This file contains detailed descriptions of all the calibration case studies, including the dynamic mathematical models. (PDF 325 kb)

Additional file 5
**Detailed results.** This file contains detailed results of the presented method for all the case studies considered. (PDF 1133 kb)

## Abbreviations

NLS: Nonlinear least squares
ODE: Ordinary differential equation
LASSO: Least absolute shrinkage and selection operator
FIM: Fisher information matrix
eSS: Enhanced scatter search
DP: Discrepancy principle
DMP: Modified discrepancy principle
TMP: Transformed discrepancy principle
MER: Monotone error rule
QO: Quasi Optimality
BP: Balancing Principle
HBP: Hardened Balancing Principle
LCC: L-curve method based on maximal curvature detection
LOO: Leave One Out cross validation
GCV: Generalized cross validation
RGCV: Robust generalized cross validation
SRGCV: Strong robust generalized cross validation
BBG: Biomass batch growth model
FHN: FitzHugh-Nagumo model
GOsc: Goodwin’s oscillator model
TSMP: Three-step metabolic pathway model
SMS: Simple multi-start
AMS: Advanced multi-start
