# Supplementary material for: Robust and efficient parameter estimation in dynamic models of biological systems
Source: BMC Syst Biol. 2015 Oct 29;9:74. doi: 10.1186/s12918-015-0219-2 (PMC4625902; doi:10.1186/s12918-015-0219-2)
Supplement: Additional file 1 — Detailed description of methods. This file contains detailed descriptions of the methods and associated computational details. (PDF 251 kb) [file 12918_2015_219_MOESM1_ESM.pdf]

Additional File 1 for
Robust and Efficient Parameter Estimation in Dynamic Models of Biological Systems
Numerical methods - details

Attila Gábor and Julio R. Banga
IIM-CSIC. Eduardo Cabello 6, 36208, Vigo, Spain

September 8, 2015

S.1 High quality Jacobian computation

In the local phase of the hybrid optimization method, the NL2SOL algorithm is used, which requires the Jacobian of the residuals vector (R). As an alternative to the simple forward finite difference method:

$$J_i(\theta) \approx \frac{R(\theta + \delta\theta_i) - R(\theta)}{||\delta\theta_i||}, \text{ for } i = 1, 2 \ldots N_\theta, \tag{S.1.1}$$

where $\delta\theta_i$ is a perturbation in the i-th parameter value, the parametric sensitivity based calculation produces a Jacobian of higher quality. The sensitivity of the model output vector (y) to the parameters has to be computed:

$$J(\theta) = \frac{dR(\theta)}{d\theta} = \frac{1}{\sigma}\frac{dy}{d\theta} \ . \tag{S.1.2}$$

Note that the Jacobian $J(\theta)$ is a matrix of size $N_D \times N_\theta$, where N_D is the number of data points and N_θ is the number of parameters, such that $[J(\theta)]_{ij}$ is the weighted sensitivity of the model prediction for the i-th data point with respect to the j-th model parameter.

The parametric sensitivities of the model outputs can be computed from the sensitivities of the state-variables as

$$\frac{dy}{d\theta_i} = \sum_{j=1}^{N_x} \frac{\partial g(x,\theta)}{\partial x_i} x_{j\theta_i} + \frac{\partial g(x,\theta)}{\partial \theta_i}, \text{ for } i = 1, 2 \ldots N_\theta, \tag{S.1.3}$$

where $g(\cdot)$ is the observation function (see Eq. (2) in the main text) and $x_{j\theta_i} = \frac{dx_j}{d\theta_i}$ denotes the sensitivity of the j-th state variable with respect to the i-th parameter.

The sensitivities of the state-variables with respect to the model parameters can be obtained by solving the so-called *forward sensitivity equations* (FSEs),

which is a well-known method in the perturbation theory of differential equations. The FSEs read as

$$\begin{aligned} \frac{dx_{\theta_i}}{dt} &= \frac{\partial f(u,x,\theta)}{\partial x} x_{\theta_i} + \frac{\partial f(u,x,\theta)}{\partial \theta_i} \\ x_{\theta_i}(t_0) &= \begin{cases} 1, & \text{if } \theta_i \text{ is initial condition} \\ 0, & \text{otherwise} \end{cases} . \end{aligned} \tag{S.1.4}$$

Note that this means that $N_\theta \times N_x$ equations have to be solved, so it is a computationally expensive calculation, which increases with the number of state variables and parameters. When the Jacobian is required, these equations are usually solved together with the dynamic model equations, because they share the system's Jacobian ($\frac{\partial f(u,x,\theta)}{\partial x}$) and thus the two systems of equations have the same "stiffness". Note that modern solvers like CVODES [1] implement this type of computation.

The system's Jacobian and the inhomogeneous part ($\frac{\partial f(u,x,\theta)}{\partial \theta_i}$) of (S.1.4) can be derived analytically (symbolically) from the dynamic equations prior to the model calibration. This procedure increases the speed and robustness of the initial value problem (IVP) and sensitivity computations. It should also be noted that the Jacobian computation by forward sensitivity equations requires a similar computational effort than the finite difference (FD) method, but in the case of the sensitivity based computation the error in the Jacobian is controlled. However, in the case of FD method, the error is unknown.

We compared the FSE and the FD methods based on the case studies presented in the main text, confirming that the accuracy of the Jacobian has a significant effect on the convergence of NL2SOL. A high quality Jacobian computation resulted in faster convergence with better chances of obtaining the global optima. However, we also observed that NL2SOL implements an intelligent adaptive scheme to tune the perturbation parameter ($\delta\theta_i$) for the FD method (S.1.1); for small problems and with careful settings, it can be almost as efficient as solving the forward sensitivity equations.

S.2 Regularization schemes

Figure S.2.1 shows a summary of the proposed regularization schemes based on the available prior knowledge quality.

S.3 Prediction error measure

To measure the prediction error, we used the following normalized root mean square error formula

$$\text{NRMSE} = \sqrt{\frac{1}{N_D} \sum_{k=1}^{N_\text{e}} \sum_{j=1}^{N_{y,k}} \frac{\sum_{i=1}^{N_{t,k,j}} \left(y_{ijk} - \tilde{y}_{ijk}\right)^2}{(\max_i \tilde{y}_{ijk} - \min_i \tilde{y}_{ijk})^2}} \ . \tag{S.3.5}$$

Here N_D is the total number of data points, N_e is the number of experiments, $N_{y,k}$ is the number of observables in the k-th experiment, $N_{t,k,j}$ is the number of time points in the k-th experiments for the j-th observable, y_{ijk} is the

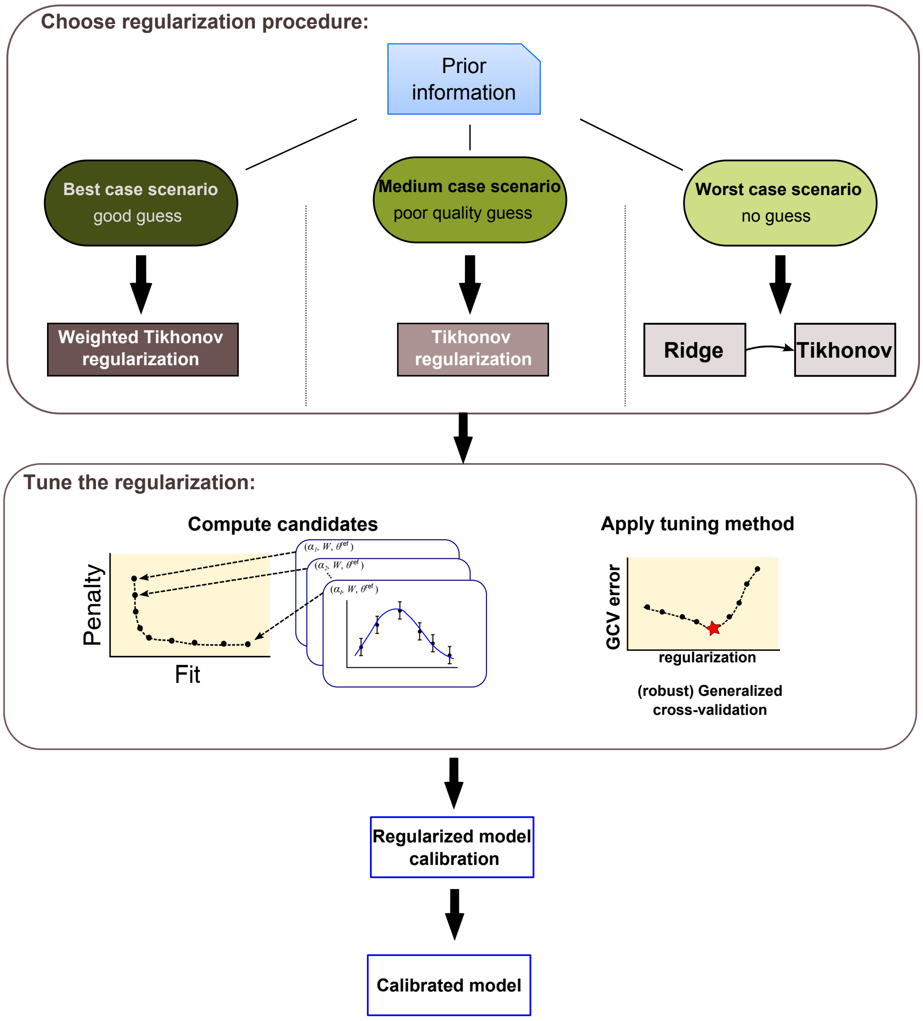

Figure S.2.1: **Regularization scheme**. Three scenarios are considered based on the quality of the available prior information: (i) best case scenario (a good guess of the parameter values is available in the literature) where a first order weighted Tikhonov regularization is recommended, (ii) medium case scenario (less reliable initial guess, but within one order of magnitude of the true values) where non-weighted Tikhonov regularization is recommended, and (iii) worst case scenario (no prior knowledge and therefore random guess of parameters) where a two-step regularization procedure is proposed. In the first step ridge regularization is applied which results the parameter vector with minimum norm, that fits the data reasonably well. In the second step this parameter vector is used as the reference parameter vector for Tikhonov regularization. In each scenario the regularized optimization is solved for a set of regularization parameter and the generalized cross validation method (GCV) is applied to choose the optimal candidate.

model prediction for the data $\tilde{y}_{ijk}$. This formula computes the root of the sum of squared error between model prediction and data for each observable, and normalizes it by the squared range of the data corresponding to that observable. In this way, the observables are properly scaled.

S.4 Initial guess calculation for global optimization

The search for the global optima of the objective function is restricted to a N_θ dimensional box. This box is specified by the lower and upper bounds of the parameters, i.e. $\theta_{min} \leq \theta \leq \theta_{max}$. In the main text, we mention 4 strategies to generate points (initial guesses) from this box:

1. Multivariate uniform distribution, which selects points from the box with uniform probability.
2. Multivariate log-uniform distribution. This can be applied only for positive bounds. First, the bounds are transformed to the logarithmic space ($[\theta_{min}\ \theta_{max}] \rightarrow [log_{10}(\theta_{min})\ log_{10}(\theta_{max})]$) and then points are selected with equal probability in the transformed box. Finally the points are transformed back to the original space.
3. Latin hypercube sampling (LHS) [2]
4. Logarithmic Latin hypercube sampling, which transforms the bounds as in 2. and then applies the LHS method.

The logarithmic scaling of the bounds is especially useful if the lower and upper bounds are different by more than an order of magnitude and we would like to collect samples from all the range of magnitudes. With the logarithmic scaling, each order of magnitude in the range of the parameters will have equal chance to contain the selected points.

S.5 Robust computation of the regularization candidates

Due to the non-convexity of the cost function, none of the stochastic global search algorithms can guarantee that the global minimum of regularized optimization problem is found. The global optimization problem is solved multiple times with regularization to generate the candidates. We can detect inconsistencies among these solutions to find cases that did not converge to the global optima.

We can use a simple and well-known observation from bi-criteria optimization to filter out incorrect solutions due to convergence to local optima. If the bi-criteria optimization (here the criteria are formulated by the least squares term Q_{LS} and the penalty Γ) is solved by weighting (here the weighting parameter is the regularization parameter), then the solutions are located on a convex curve (the points of which are then $(Q_{LS}(\hat{\theta}_{\alpha_i}), \Gamma(\hat{\theta}_{\alpha_i}))$ for $i = 1 \ldots I$). This convex curve is referred as the L-curve in regularization theory (and in the main text), but it is also known as the Pareto front in multi-objective optimization.

For example, in case of two estimated parameter vectors $\hat{\theta}_{\alpha_1}$ and $\hat{\theta}_{\alpha_2}$ corresponding to two regularization parameter values $\alpha_1 > \alpha_2$ is expected to have the following relations: $Q_{LS}(\hat{\theta}_{\alpha_1}) > Q_{LS}(\hat{\theta}_{\alpha_2})$ –i.e. larger regularization leads to worse model fit–, and $\Gamma_T(\hat{\theta}_{\alpha_1}) < \Gamma_T(\hat{\theta}_{\alpha_2})$, i.e. larger regularization gives smaller penalty function value. If the relation is not fulfilled, then one of the solution dominates the other and the dominated solution is not a global solution of the corresponding optimization problem, but is the artefact of local convergence.

A straightforward strategy would be to detect and remove the dominated points from the Pareto front obtained by independent estimations. But this would discard valuable computational results. Instead, we applied the following iterative search strategy. We assume that a set of regularization parameters $\alpha_1 > \alpha_2 > \ldots \alpha_I$ are already selected for which the optimization problem (Equation (7) in the main text) has to be solved, for example using the procedure described in the main text.

The penalty term is a quadratic, therefore convex function of the parameters, which takes its unique (global) minimum at the reference parameter vector θ^{ref}. On the other hand, the first part of the objective function $Q_{\mathrm{LS}}(\theta)$ can be highly multi-modal with many local minima. Therefore, it is recommended to start the search with the largest regularization parameter α_1.

A procedure to compute a smooth L-curve.

Step 1. **Global, sequential forward search.** Solve the optimization problem ((7) in the main text) one-by-one for $\alpha_1, \alpha_2, \ldots \alpha_I$ using the proposed global optimization meta-heuristic (eSS2) such that, in the i-th run the previously obtained parameters $\{\hat{\theta}_{\alpha_1}, \hat{\theta}_{\alpha_2}, \ldots \hat{\theta}_{\alpha_{i-2}}, \hat{\theta}_{\alpha_{i-1}}\}$ are included in the initial guess set for the global search. This can save a large amount of time in the global search of the next optimal point, preventing dominated solutions.

Remark 1. Note that, as we solve a sequence of non-linear optimization problems with a global search method, it is possible, that at some point a much better optimum is reached; for example $\hat{\theta}_{\alpha_k}$, which reveals that, the previously obtained points $\hat{\theta}_{\alpha_i}$ for $i = 1, 2, \ldots k-1$ are dominated, i.e. they have not converged to the global optima. But the reverse cannot happen, i.e. as mentioned above $\hat{\theta}_{\alpha_i}$ cannot be dominated by any of $\hat{\theta}_{\alpha_{i-1}} \ldots \hat{\theta}_{\alpha_1}$ since they are used as initial guesses in the search.

Step 2. **Backward search.** In the second step we refine the optima one-by-one, but *in the reverse order* $\alpha_{I-1}, \ldots \alpha_1$. Here, only two local searches are performed for each regularization parameter. The initial guess for these searches are the parameters obtained in Step 1: for the optimization with α_i the initial guesses are $\hat{\theta}_{i+1}$ and $\hat{\theta}_i$.

Remark 2. Note that, the reverse order optimization with the selected initial guesses eliminates the dominated points of the Pareto front that may appeared in Step 1.

S.6 Bias-variance computation

In Section 3.5 in the main text we used multiple sets of calibration data (replicates) to investigate the effect of the random noise in the data to the model prediction and parameter estimation. Calibrating the model on these multiple data sets results in a set (population) of estimated parameter vectors (each vector corresponds to a dataset). Using these estimated parameter vectors, we obtain a population of predictions. Based on these quantities, we can then compute the parameter estimation and prediction bias and variance using:

$$\text{variance}(\hat{\theta}_\alpha) = \text{trace}\left(\mathbb{E}\left[(\hat{\theta}_\alpha - \mathbb{E}[\hat{\theta}_\alpha])(\hat{\theta}_\alpha - \mathbb{E}[\hat{\theta}_\alpha])^T\right]\right) \tag{S.6.6}$$

$$\text{variance}(\hat{y}_\alpha) = \text{trace}\left(\mathbb{E}\left[(\hat{y}_\alpha - \mathbb{E}[\hat{y}_\alpha])(\hat{y}_\alpha - \mathbb{E}[\hat{y}_\alpha])^T\right]\right) \tag{S.6.7}$$

$$\text{bias}^2(\hat{\theta}_\alpha) = \text{trace}\left((\mathbb{E}[\hat{\theta}_\alpha] - \theta_t)(\mathbb{E}[\hat{\theta}_\alpha] - \theta_t)^T\right) \tag{S.6.8}$$

$$\text{bias}^2(\hat{y}_\alpha) = \text{trace}\left((\mathbb{E}[\hat{y}_\alpha] - y_t)(\mathbb{E}[\hat{y}_\alpha] - y_t)^T\right), \tag{S.6.9}$$

where $\hat{\theta}_\alpha$ is the estimated parameters with regularization parameter α, $\hat{y}_\alpha$ is the model prediction based on this parameter estimate, i.e. $\hat{y}_\alpha = y(\hat{\theta}_\alpha)$ and y_t, θ_t are the true (nominal) prediction and parameter vectors, respectively. This true values are known only for synthetic problems and used only for the bias-variance analysis.

S.7 Settings of the optimization algorithms

Table S.7.1 shows the default settings for both the global optimization algorithm (eSS) and the local NLS algorithm (NL2SOL). Note that these values might be different from the default values of the algorithms, but the same values have been used for all the case studies (i.e. they were found to be robust settings). Interested readers can find further tuning details in eSS User's Manual and the NL2SOL User's Guide [3].

Optimization runs terminate when at least one stopping criteria is reached. The most frequently activated stopping criteria for the global optimization was either the allowed computation time or the allowed number of objective function evaluation. The allowed computation time and number of function evaluations are reported for each case study in Additional File 2.

Table S.7.1: **Default optimization settings for the case studies.** eSS settings: `ndiverse` is the number of diverse solutions generated, `n1` is the number of global iteration before the local algorithm is called for the first time, `n2` is the number of global iteration between consecutive calls of the local algorithm, `local_balance` influences the selection of starting point among the members of the population for initiating the local optimization, `log_var` generates the initial and new members of the population in the logarithmic scaled bounds of the parameters. NL2SOL settings: `maxfuneval` is the maximum number of function evaluation before the search terminates, `maxiter` is the maximum iteration number before termination, `tolrfun` is the relative tolerance (the algorithm terminates if the approximated global optima is within this tolerance value), `tolobjr` is the computational accuracy of the objective function and the Jacobian (which is tuned to the tolerance level of the ODE solver tolerance level).

**eSS settings**	**value**	**NL2SOL settings**	**value**
`ndiverse`	$10 \cdot N_\theta$	maxfuneval	300
`n1`	1	maxiter	200
`n2`	10	tolrfun	10^{-6}
`local_balance`	0.5	tolobjr	10^{-5}
`log_var`	$\text{ones}(1, N_\theta)$		

References

[1] Hindmarsh, A.C., Brown, P.N., Grant, K.E., Lee, S.L., Serban, R., Shumaker, D.E., Woodward, C.S.: SUNDIALS: Suite of Nonlinear and Differential/Algebraic Equation Solvers. ACM Transactions on Mathematical Software **31**(3), 363–396 (2005)

[2] McKay, M.D., Beckman, R.J., Conover, W.J.: A comparison of three methods for selecting values of input variables in the analysis of output from a computer code. Technometrics **42**(1), 55–61 (2000)

[3] Gay, D.M.: Usage Summary for Selected Optimization Routines. AT&T Bell Laboratories Murray Hill, NJ 07974, Computing Science Technical Report No. 153 (1990)
