# Supplementary material for: Robust and efficient parameter estimation in dynamic models of biological systems
Source: BMC Syst Biol. 2015 Oct 29;9:74. doi: 10.1186/s12918-015-0219-2 (PMC4625902; doi:10.1186/s12918-015-0219-2)
Supplement: Additional file 2 — Regularization tuning methods. This file contains further details and results about the regularization tuning methods. (PDF 300 kb) [file 12918_2015_219_MOESM2_ESM.pdf]

Additional File 2 for
Robust and Efficient Parameter Estimation in Dynamic Models of Biological Systems
Methods for tuning regularization

Attila Gábor and Julio R. Banga
IIM-CSIC. Eduardo Cabello 6, 36208, Vigo, Spain

October 19, 2015

S2.1 The plethora of regularization tuning methods

Choosing a method to tune regularization is non-trivial. Bauer and Lukas[1] compared 17 regularization parameter choice methods for a large set of linear least squares problems. Apart from the numerical case studies, they also provided a unified framework to discuss the numerical aspects of each method, and the performance of the regularization methods from the estimated parameters point of view. We can see that most of the methods work very well in terms of achieving a small mean square error in the estimated parameters. However, small parameter estimation error do not imply small prediction errors.

Most of the regularization tuning methods can be used together with nonlinear least squares (NLS) methods, such as e.g. the Landweber algorithm, the iteratively regularized Gauss Newton method or conjugate gradient method [2]. These optimization methods iteratively update the parameter estimates by a suitable step for which the residual vector or the objective function (depending on the method) is linearized in each step and the regularization is applied to the steps. Our approach is different, since we apply the regularization directly to the parameter vector instead of the updating steps, which allow us to use the regularization in both the global and the local optimization phases.

In the following section we shortly summarize the regularization tuning methods considered and their computational details, which we utilized for the Tikhonov regularization of the hybrid optimization algorithm presented in the main text. In the following sections we also report the results of the regularization tuning methods for each case study. For each problem, the regularization parameter selected by the regularization tuning method is compared against the bias-variance trade-off curves (both for the bias-variance in the estimated parameters and in the estimated predictions). Note that the performance of a tuning method is evaluated based on the distance of the selected regularization parameter from the location of the minimum of the bias-variance trade-off curves.

S2.2 Tuning methods

In this section we utilize the following notations. We consider a set of regularization parameters as $\alpha_1 > \alpha_2 > \dots > \alpha_i > \dots \alpha_I > 0$. In practice this set of regularization parameters constitutes a geometric series, i.e. $\alpha_{i+1}/\alpha_i = q < 1$ for $\forall i$. The estimated parameter vector $(\hat{\theta}_{\alpha_i}(y^\delta))$ obtained by the calibration of the kinetic model to the experimental data (y^δ) using the regularization parameter α_i is denoted shortly by θ_i^δ, where δ indicates the measurement noise level in the data. The total number of measurement data is denoted by N.

The residual vector evaluated at the estimated parameter is $R_i = R(\theta_i^\delta) = \frac{y(\theta_i^\delta)-y^\delta}{\sigma}$ and its Jacobian matrix also evaluated at the estimated parameter is denoted by $J_i = \frac{\partial R_i}{\partial \theta}$. Further, $||x||$ denotes the Euclidean norm of the vector x.

S2.2.1 Discrepancy principle

The discrepancy principle [3] is one of the most used and analysed tuning method. The discrepancy principle selects the largest regularization parameter for which the discrepancy between the model prediction and the data is similar to the measurement error. Thus it avoids over-fitting of the model. It is known that the discrepancy principle requires an accurate knowledge of the measurement error.

The optimal regularization parameter selected by the discrepancy principle is the first regularization parameter (α_i) for which

$$||R_i|| \le \tau\delta\sqrt{N}, \tag{S2.2.1}$$

where $\tau > 1$ is a tuning parameter and δ is the standard deviation of the elements of the residuals (the measurement error). It is non-trivial to select the τ tuning parameter, since if the real measurement error is larger than $\tau\delta$, the method is unstable, and therefore $\tau > 1$ should be used [4]. On the other hand, $\tau > 1$ can over-regularize the solution [5]. We used $\tau = 1.5$ as in [1]. The principle has the appealing simplicity of computation, which do not require any linearization.

In our formalism the residual vector is weighted by the inverse of the standard deviation of the data (see Equation (3) in the main text). Thus, our a priori knowledge about the measurement error is already in the formulated objective function and so we set $\delta = 1$.

Results. We found that the discrepancy principle selected too large regularization parameters in most of the case studies (for both the parameter estimates and model predictions).

S2.2.2 Transformed discrepancy principle

In the case of the transformed discrepancy principle, instead of comparing the observed error in the fit to some a priori level (as in (S2.2.1)), the residual error is transformed to the parameter space and measured against an approximated bound to the parameter estimation error [1]. This procedure is more stable than the discrepancy principle and less sensitive to correct knowledge of the

measurement error [6]. The transformed discrepancy principle in our linearised framework reads as

$$||(J_i^T J_i + \alpha_i W^T W)^{-1} J_i^T R_i|| \leq b\delta \frac{\sqrt{N}}{\sqrt{\alpha_i}} \qquad \text{(S2.2.2)}$$

where b is a constant tuning parameter (0.4872 as in [1]).

Results. We found that the transformed discrepancy principle also selected too large regularization parameters in most of the case studies for both the parameter estimates and model predictions.

S2.2.3 Modified discrepancy principle

The mean squared error in the parameter estimates can be decomposed into the sum of (i) propagated error and (ii) regularization error. The inverse operation amplifies the measurement error in the data, which can cause large error in the estimated parameters. This type of error is called the *propagated error* and it decreases with larger regularization parameter. The *regularization error* is caused by the fact, that the true parameters are unknown, therefore the regularization term biases the estimates towards the reference parameters vector. This bias increases with increasing regularization parameter.

Gfrerer [7] and later Engl and Gfrerer [8] have developed a method which minimizes the mean squared error of the parameter estimates by taking the derivatives of the regularization error and the propagated error by the regularization parameter. The method is also known as the minimum bound method. When the differentiation by the regularization parameter is approximated by finite differences ($d\theta_i/d\alpha_i \approx \dfrac{\theta_i - \theta_{i+1}}{-\alpha_i \log(q)}$, where $q = \alpha_{i+1}/\alpha_i$ as in [1]), the method chooses the largest regularization parameter for which

$$\sqrt{\frac{\alpha_i}{-\log(q)}} ||R_i^T (J_i J_i^T + \alpha_i I_N)^{-1} (\theta_i - \theta_{i+1})|| \leq \tau\delta, \qquad \text{(S2.2.3)}$$

where I_N is the N-dimensional identity matrix and τ is a tuning parameter, which was set to 1.5.

Results. When the selected candidate was compared to the bias-variance trade-off curves, we found that the method performed well for the 3SMP, CHM, ScCHM, slightly over-regularized for the problems MAPK, TGFB, GOsc and BBG, but severely over-regularized the problem comparing to the bias variance trade-off for the problems AP and FHN.

S2.2.4 Monotone error rule

The monotone error rule [9] uses the fact that for large regularization the mean parameter estimation error is dominated by the regularization error, which gradually decreases as the regularization parameter decreases. After adapting the formula from [1] for the linearized framework, the method choose the largest regularization parameter for which

$$\frac{||R_i^T (J_i J_i^T + \alpha_i I_N)^{-1} (\theta_i - \theta_{i+1})||}{||(J_i J_i^T + \alpha_i I_N)^{-1} (\theta_i - \theta_{i+1})||} \leq \tau \sqrt{N} \delta, \qquad \text{(S2.2.4)}$$

where I_N is the N-dimensional identity matrix and τ is a tuning parameter, which was set to 1.5.

Results. We observed that this tuning method resulted in small mean squared parameter estimation error for linear problems, but large prediction errors. In all the case studies the largest regularization parameter was selected, which is a severe over-regularization.

S2.2.5 Balancing principle and hardened balancing principle

The balancing principle [10] equalize the upper bound of the propagated error with the regularization error. The propagated error bound $\delta^2\rho_i^2$ for the mean squared parameter estimation error in case of Tikhonov regularization and uncorrelated noise is

$$\delta^2\rho_i^2 = \sum_{l=1}^{N_\theta} \left(\frac{\sigma_{l,i}}{\sigma_{l,i}^2 + \alpha_i} \right)^2 , \tag{S2.2.5}$$

where $\sigma_{1,i}$, $\sigma_{2,i}$ $\ldots \sigma_{N_\theta,i}$ are the singular values of the Jacobian matrix J_i. The regularization error is estimated by the differences of the subsequent parameter candidate vectors as $||\theta_i - \theta_{i+1}||$.

Then a balancing functional is defined for each regularization parameter as

$$b(n) = \max_{n<i\leq I_{max}} \frac{||\theta_i - \theta_{i+1}||}{4\delta\rho_i} \tag{S2.2.6}$$

and its smooth, monotonically decreasing variant $B(n) = \max_{n<i\leq I_{max}} b(n)$.

The balancing rule choose the first regularization parameter for which $B(n) < \tau$, where τ is a tuning constant ($\tau = 1$).

In case of hardened balancing [11] the regularization parameter is selected for which $B(n)\sqrt{\rho_n}$ takes its minimum. Thus the hardened balancing principle does not need a tuning parameter and the estimate of the measurement error δ is also not required.

Results. In linear ill-posed problems the parameter estimates usually grow very fast as the regularization parameter decreases, which results in (exponentially) growing values for $||\theta_i - \theta_{i+1}||$. However, this was not observed in our (non-linear) case studies. Rather, we observed some saturation in the parameter values as the regularization decreases. This is probably the reason why both the balancing principle and the hardened balancing principle performed poorly.

S2.2.6 Quasi optimality

The quasi optimality principle [12] use the fact that for large regularization parameter the differences between the subsequent parameter estimates ($||\theta_i - \theta_{i+1}||$) are large due to the large regularization error. On the other hand, for very small regularization parameters, large values for ($||\theta_i - \theta_{i+1}||$) is expected, because of the ill-conditioning and large variance of the estimates. Thus the

quasi optimality criteria choose the regularization parameter for which the differences are minimized as

$$\alpha_{opt} = \arg \min_{\alpha_i > \alpha_{I_{max}}} ||\theta_i - \theta_{i+1}|| \tag{S2.2.7}$$

here I_{max} is a maximum index (i.e. $\alpha_{I_{max}}$ is a minimal regularization parameter) that is a required user input. Note that for very small regularization parameter we would not observe any differences between the parameter estimates and thus the difference would be numerically zero.

Results. It was difficult to automatically find a proper minimum index for the regularization parameter, which caused under-regularized solutions for some of the case studies. Although the method worked reasonably well for the MAPK case study, in the other cases resulted in either under regularized or over-regularized parameter estimates.

S2.2.7 L-curve methods

Hansen [13, 14] developed the L-curve method for tuning the regularization of ill-posed problems. When the norm of the parameter estimates ($||\theta_i||$) is plotted against the norm of the residuals ($||R_i||$) for the set of regularization candidates $i = 1, 2 \ldots I$, one can visualize the set of points in the Pareto optimal front. In the linear case the points make up an L-shaped curve. The horizontal part is formed by the solutions corresponding to large regularization parameters, where the regularization bias is dominating. On the vertical part of the L-curve a small reduction in the least-squares error usually causes a large increase in the parameters and the propagated error is dominating the mean squared parameter estimation error. Intuitively, the optimal regularization parameter that balances the two types of error is located near the corner of the L-shaped curve. The corner point can be identified by detecting the largest curvature of the points $||\theta_i||, ||R_i||$ as in [13, 12] (will be denoted by LCC), or where the slope of the tangent of the point equals to minus one [15] (referenced as LCR).

The curvature of the points can be calculated using finite differences of the points, for which we used the lineCurvature2D tool for MATLAB. The Reginska version is equivalent to find the optimal regularization parameter by

$$\alpha_{opt} = \arg \min_{\alpha_i} ||\theta_i|| \cdot ||R_i|| \tag{S2.2.8}$$

Results. We have obtained rather different results for the curvature based (LCC) and the tangent slope based (LCR) L-curve methods. Typically, the LCR chose larger regularization parameters than the LCC method. Both performed well on case studies GOsc, FHN and CHM. LCR over-regularized the solutions while the LCC performed well for TGFB, MAPK, scCHM, 3SMP and AP. Both variants performed rather poorly for the BBG case study.

S2.2.8 Extrapolated error method

Brezinski [16] developed an error estimation method applying as tuning method for Tikhonov regularization. The method choose the regularization parameter

as

$$\alpha_{opt} = \arg\min_{\alpha_i} \frac{||R_i||^2}{||J^T R_i||}. \tag{S2.2.9}$$

Results. For most of the case studies the EEM gave over-regularized solutions.

S2.2.9 Residual method

Originally developed for truncated singular values decomposition based regularization [17], the residual method (RES) operates in the prediction space. First a quantity $B_i = J_i(I - (J_i J_i^T + \alpha_i I_N)^{-1} J_i^T)$ is defined and then the method chooses the regularization parameter for which the weighted residuals is minimized as

$$\alpha_{opt} = \arg\min_{\alpha_i} \frac{||R_i||}{\text{trace}(B_i^T B_i)^{1/4}} \tag{S2.2.10}$$

Results. In almost all the case studies the RES method selected the largest regularization parameter among the candidates, thus over-regularizing the solutions.

S2.2.10 Generalized cross validation methods

In the so-called leave-one-out (LOO) cross validation, a model is fitted to all but one data point in a step and the fitted model is used to predict the hold out data point. Then, the process is repeated until each data point is left out once. The model can be evaluated based on the discrepancy between the predictions and data (LOO prediction error). The generalized cross validation (GCV) technique uses the fact that there is an explicit formula for the computation of the LOO prediction error, i.e. the fitting of the linear model do not have to be repeated as many times as the number of data points.

Golub et al [18] and Wahba[19] developed the method of generalized cross validation, and a number of variants appeared later. Here we considered a variant in the linearized framework [20] as follows. The basic principle is to weight the observed residual error by the effective number of degrees of freedom. The GCV method chooses the regularization parameter as

$$\alpha_{opt} = \arg\min_{\alpha_i} \frac{||R_i||^2}{(\frac{1}{N}\text{trace}(I - J(J_i J_i^T + \alpha_i I_N)^{-1} J_i^T))^2}. \tag{S2.2.11}$$

In [18] a slightly different version appeared, which results in differences when the linearization is applied

$$\alpha_{opt} = \arg\min_{\alpha_i} \frac{||(I - J(J_i J_i^T + \alpha_i I_N)^{-1} J_i^T) R_i||^2}{(\frac{1}{N}\text{trace}(I_N - J(J_i J_i^T + \alpha_i I_N)^{-1} J_i^T))^2}. \tag{S2.2.12}$$

Two known drawbacks of the GCV criteria is its behaviour in the case of correlated noise and the flatness of the criteria near the optima, which can lead to under-regularized solutions. To overcome this issue, two variants of the GCV method, the robust generalized cross validation method (RGCV) [21] and the

strong robust generalized cross-validation (SRGCV) criteria have been developed by Lukas[22]. In our framework, we compute the optimal regularization parameter based on the RGCV criteria as

$$F_i = \mathrm{trace}(I - J_i(J_iJ_i^T + \alpha_i I_N)^{-1}J_i^T) \tag{S2.2.13}$$

$$\alpha_{opt} = \arg\min_{\alpha_i} \frac{||R_i||^2}{(\frac{1}{N}F_i)^2}(\gamma + (1-\gamma)\frac{1}{N}F^2), \tag{S2.2.14}$$

where γ is a tuning parameter (note that $\gamma = 1$ leads to the GCV formula), which was set to 0.1 as in [1]. The SRGCV criteria is computed as

$$G_i = (J_iJ_i^T + \alpha_i I_N)^{-1}J_i^T \tag{S2.2.15}$$

$$\alpha_{opt} = \arg\min_{\alpha_i} \frac{||R_i||^2}{(\frac{1}{N}J_iG_i)^2}(\gamma + (1-\gamma)\frac{1}{N}\mathrm{trace}(G_i^TG_i)), \tag{S2.2.16}$$

where the tuning parameter γ was set to 0.95.

Results. We observed that all the considered four variants of the generalized cross-validation based method resulted in similarly good results for all our case studies. Typically, the RGCV and the GCVG selected slightly larger regularization parameter than the SRGCV and the GCV rules. However, these regularization parameters were still close to the optima of the bias-variance trade-off curves.

S2.3 Biomass batch growth (BBG)

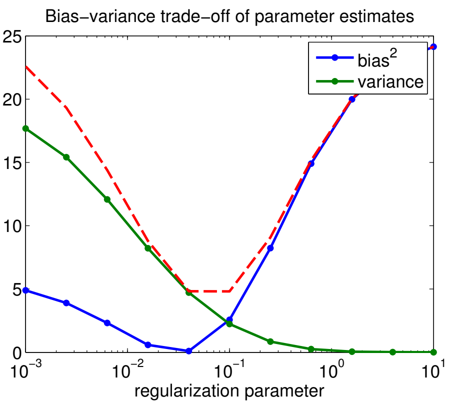

(a) Bias-variance trade-off for estimated parameters

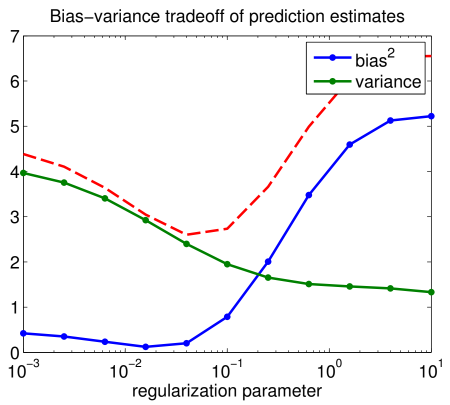

(b) Bias-variance trade-off for model prediction

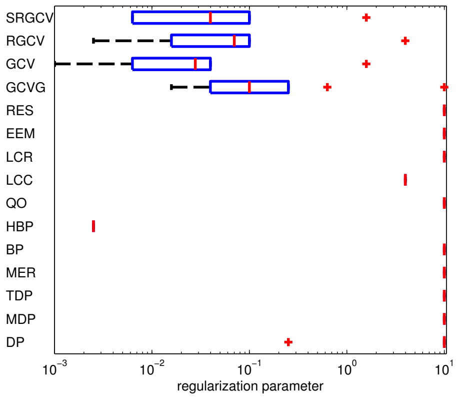

(c) Optimal regularization parameter based on different tuning methods

Figure S2.3.1: Biomass growth model. Bias-variance trade-off and regularization tuning methods.

S2.4 Goodwin Oscillator (GOsc)

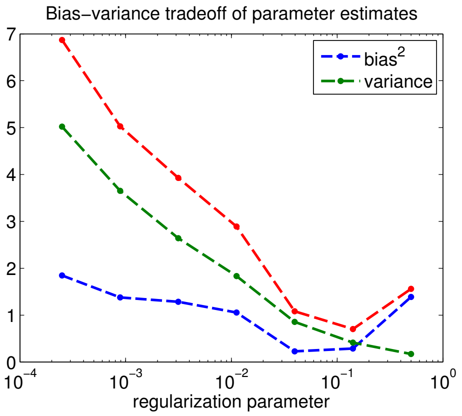

(a) Bias-variance trade-off for estimated parameters

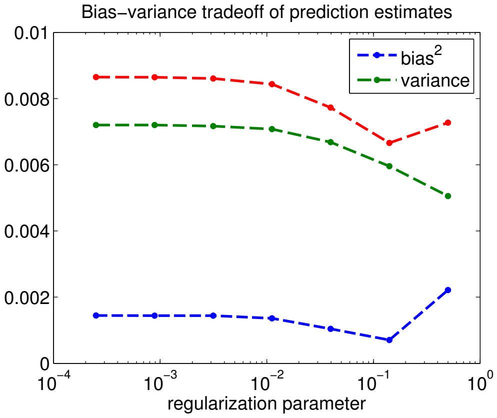

(b) Bias-variance trade-off for model prediction

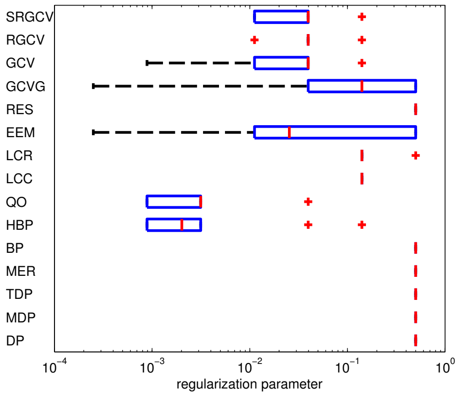

(c) Optimal regularization parameter based on different tuning methods

Figure S2.4.2: Goodwin's oscillator model. Bias-variance trade-off and regularization tuning methods.

S2.5 FitzHugh-Nagumo model (FHN)

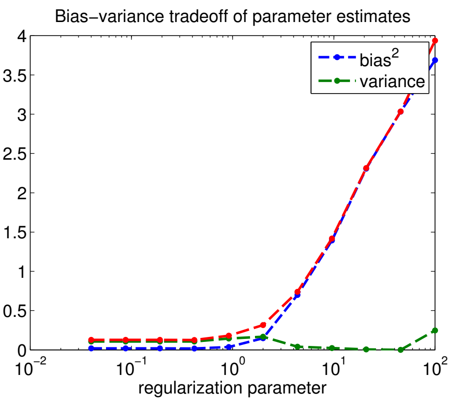

(a) Bias-variance trade-off for estimated parameters

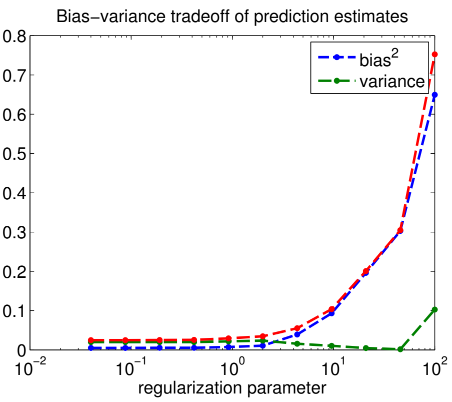

(b) Bias-variance trade-off for model prediction

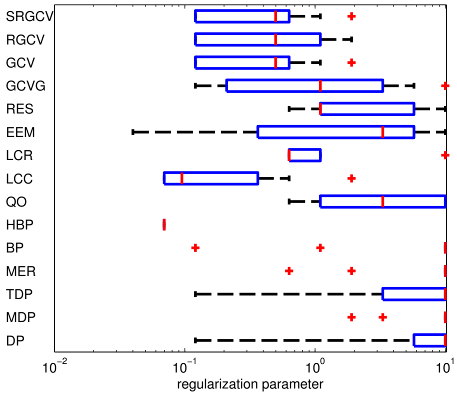

(c) Optimal regularization parameter based on different tuning methods.

Figure S2.5.3: FitzHugh-Nagumo model. Bias-variance trade-off and regularization tuning methods.

S2.6 TGF– β signalling pathway model (TGFB)

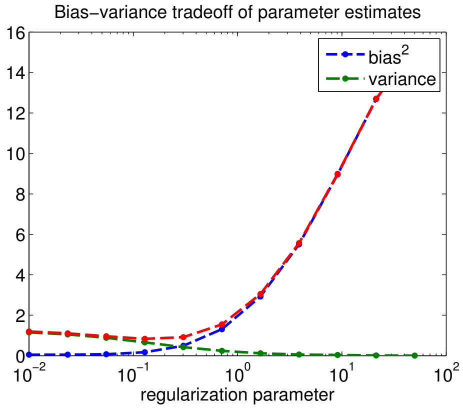

(a) Bias-variance trade-off for estimated parameters

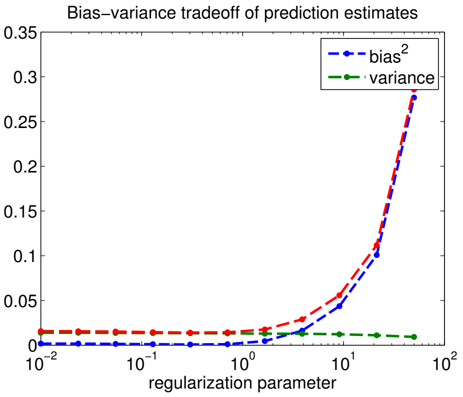

(b) Bias-variance trade-off for model prediction

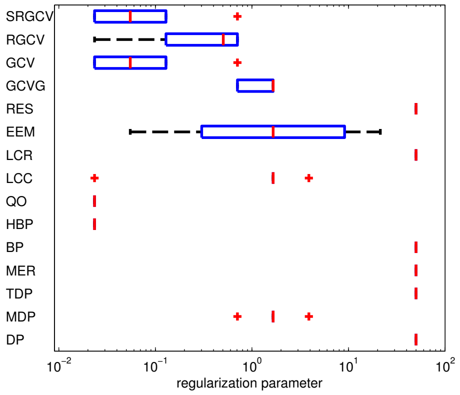

(c) Optimal regularization parameter based on different tuning methods.

Figure S2.6.4: TGF-β Signalling Pathway Model. Bias-variance trade-off and regularization tuning methods.

S2.7 Kholodenko MAPK signalling pathway (MAPK)

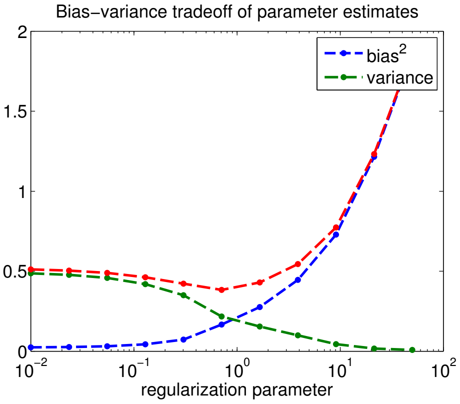

(a) Bias-variance trade-off for estimated parameters

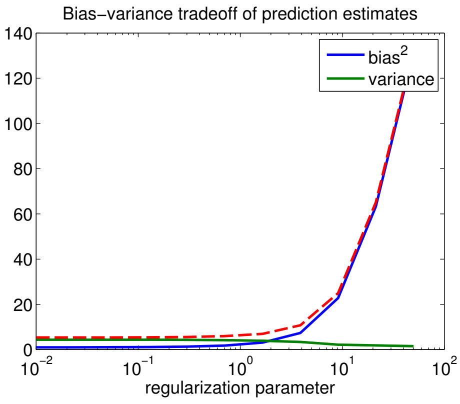

(b) Bias-variance trade-off for model prediction

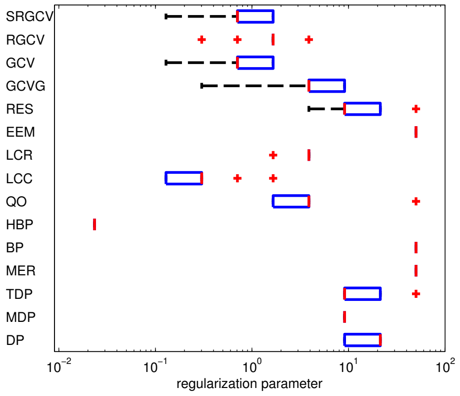

(c) Optimal regularization parameter based on different tuning methods.

Figure S2.7.5: MAPK pathway model. Bias-variance trade-off and regularization tuning methods.

S2.8 Chemotaxis signalling pathway model (CHM)

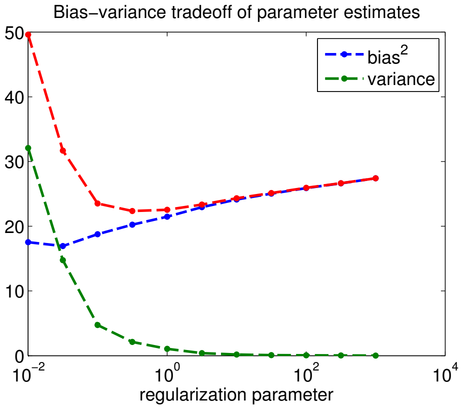

(a) Bias-variance trade-off for estimated parameters

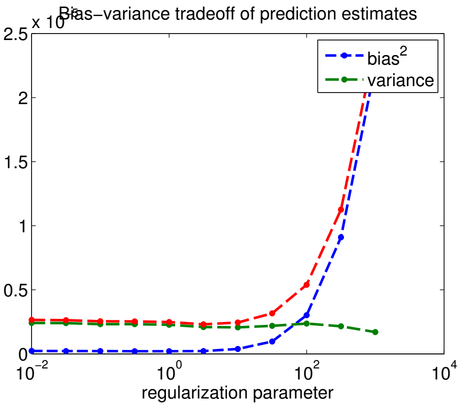

(b) Bias-variance trade-off for model prediction

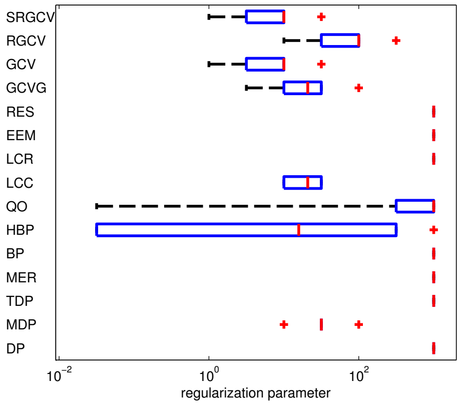

(c) Optimal regularization parameter based on different tuning methods.

Figure S2.8.6: Chemotaxis Signalling Pathway model. Bias-variance trade-off and regularization tuning methods.

S2.9 3-Step Metabolic pathway model (TSMP)

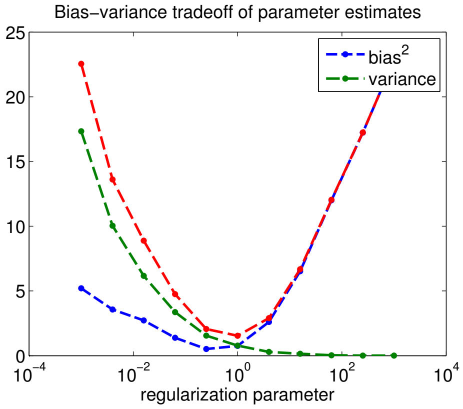

(a) Bias-variance trade-off for estimated parameters

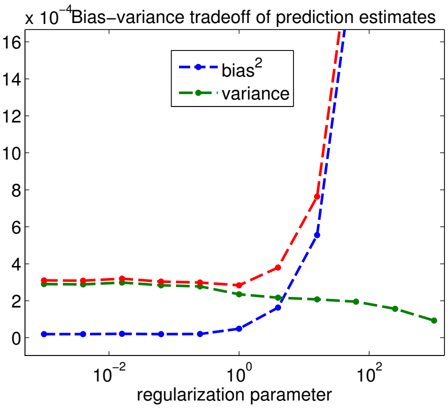

(b) Bias-variance trade-off for model prediction

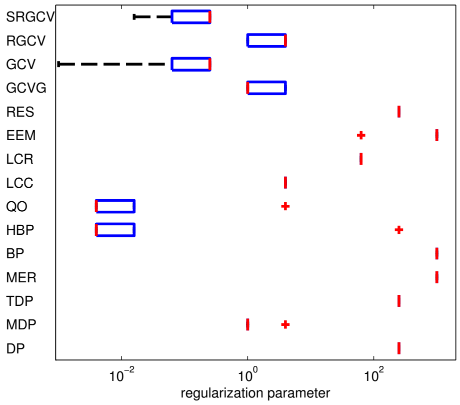

(c) Optimal regularization parameter based on different tuning methods.

Figure S2.9.7: 3-Steps Metabolic Pathway Model. Bias-variance trade-off and regularization tuning methods.

References

[1] Bauer, F., Lukas, M.A.: Comparingparameter choice methods for regularization of ill-posed problems. Mathematics and Computers in Simulation **81**(9), 1795–1841 (2011).

[2] Kaltenbacher, B., Neubauer, A., Scherzer, O.: Iterative Regularization Methods for Nonlinear Ill-Posed Problems. Radon Series on Computational and Applied Mathematics. Walter de Gruyter, Berlin, New York (2008).

[3] Morozov, V.A.: Methods for Solving Incorrectly Posed Problems. Springer, New York (1984)

[4] Lukas, M.A.: Comparisons of parameter choice methods for regularization with discrete noisy data. Inverse Problems **14**(1), 161 (1998)

[5] Vogel, C.R.: Computational Methods for Inverse Problems vol. 23. Siam, Philadelphia, PA (2002)

[6] Hämarik, U., Raus, T.: On the choice of the regularization parameter in ill-posed problems with approximately given noise level of data. Journal of Inverse and Ill-posed Problems **14**(3), 251–266 (2006)

[7] Gfrerer, H.: An a posteriori parameter choice for ordinary and iterated Tikhonov regularization of ill-posed problems leading to optimal convergence rates. Mathematics of Computation **49**(180), 507 (1987)

[8] Engl, H.W., Gfrerer, H.: A posteriori parameter choice for general regularization methods for solving linear ill-posed problems. Applied Numerical Mathematics **4**(5), 395–417 (1988)

[9] Hämarik, U., Tautenhahn, U.: On the monotone error rule for parameter choice in iterative and continuous regularization methods. BIT **41**(5), 1029–1038 (2001)

[10] Lepskii, O.: On a Problem of Adaptive Estimation in Gaussian White Noise. Theory of Probability & Its Applications **35**(3), 454–466 (1991).

[11] Bauer, F.: Some considerations concerning regularization and parameter choice algorithms. Inverse Problems **23**(2), 837–858 (2007)

[12] Engl, H.W., Hanke, M., Neubauer, A.: Regularization of Inverse Problems, pp. 1–329. Kluwer Academic Publisher, Dordrecht (1996)

[13] Hansen, P.C.: Analysis of Discrete Ill-Posed Problems by Means of the L-Curve. SIAM Review **34**(4), 561–580 (1992)

[14] Hansen, P.C., O'Leary, D.P.: The use of the L-Curve in the regularization of discrete ill-posed problems. SIAM Journal on Scientific Computing **14**(6), 1487–1503 (1993)

[15] Regińska, T.: A Regularization Parameter in Discrete Ill-Posed Problems. SIAM Journal on Scientific Computing **17**(3), 740–749 (1996)

[16] Brezinski, C., Rodriguez, G., Seatzu, S.: Error estimates for linear systems with applications to regularization. Numerical Algorithms **49**, 85–104 (2008)

[17] Bauer, F., Mathe, P.: Parameter choice methods using minimization schemes. Journal of Complexity **27**, 68–85 (2011).

[18] Golub, G.H., Heath, M.T., Wahba, G.: Generalized cross-validation as a methodforchoosingagood ridge parameter,. Technometrics **21**, 215–223 (1979)

[19] Wahba, G.: Practical Approximate Solutions to Linear Operator Equations When the Data are Noisy. SIAM Journal on Numerical Analysis **14**(4), 651–667 (1977)

[20] O'Sullivan, F., Wahba, G.: A cross validated bayesian retrieval algorithm for nonlinear rempte sensing experiments. Journal of Computational Physics **59**(3), 441–455 (1985)

[21] Lukas, M.A.: Robust GCV choice of the regularization parameter for correlated data. The Journal of integral equations and applications **22**(3), 519–547 (2010)

[22] Lukas, M.A.: Strong robust generalized cross-validation ot choosing the regualrization parameter. Inverse Problems **24**(3) (2008)
