# Supplementary material for: Robust and efficient parameter estimation in dynamic models of biological systems
Source: BMC Syst Biol. 2015 Oct 29;9:74. doi: 10.1186/s12918-015-0219-2 (PMC4625902; doi:10.1186/s12918-015-0219-2)
Supplement: Additional file 4 — Numerical case studies. This file contains detailed descriptions of all the calibration case studies, including the dynamic mathematical models. (PDF 325 kb) [file 12918_2015_219_MOESM4_ESM.pdf]

Additional File 4 for
Robust and Efficient Parameter Estimation in Dynamic Models of Biological Systems
Description of the case studies

Attila Gábor and Julio R. Banga
IIM-CSIC. Eduardo Cabello 6, 36208, Vigo, Spain

October 19, 2015

S4.1 Summary

Here we report the details of the case studies used in the main text. Each section considers a case study, presenting first the **mathematical model** (as a kinetic model based on ordinary differential equations), together with the nominal values of the model parameters. Then we discuss how the pseudo-experimental data was obtained. Usually more than one set of experimental data was considered: one set for calibration, and another set for cross-validation. The **experimental conditions** subsections give details on sampling times, stimuli levels and profiles, initial conditions, etc. The resulting data sets can be found in Additional File 2. Finally, the **calibration** subsections give details on the estimated parameters, numerical settings of the solvers and other practical considerations.

S4.2 Biomass batch growth (BBG)

S4.2.1 Mathematical model

This model describes microbial growth in a stirred fed-batch bioreactor as described by Rodriguez-Fernandez [1], but neglecting the inflow. It is a simple description of the conversion of substrate to biomass. The simplified scheme of the reactions can be seen in figure S4.2.1. The dynamic equations and observables are written as

$$\begin{aligned}
\frac{dC_b}{dt} &= \mu_{\max}\frac{C_s C_b}{K_s + C_s} - k_d C_b \\
\frac{dC_s}{dt} &= -\frac{\mu_{\max}}{\text{yield}}\frac{C_s C_b}{K_s + C_s} \\
g_1(t) &= C_b(t) \\
g_2(t) &= C_s(t)
\end{aligned} \tag{S4.2.1}$$

where C_b is the concentration of the microbes and C_s denotes the concentration of substrate. Both states can be observed. The model parameters and their nominal values can be found in Table S4.2.1

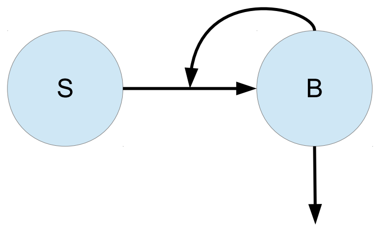

Figure S4.2.1: Biomass batch growth model. Substrate S is transferred to biomass B.

Table S4.2.1: Biomass batch growth model parameters, nominal values and optimization bounds.

Par. id	Nominal	UB	LB
$\mu_{\max}$	0.4	100	10^{-5}
K_s	5	100	10^{-5}
K_d	0.05	100	10^{-5}
yield	0.5	100	10^{-5}

S4.2.2 Experimental conditions

Here we discuss how the data was generated for model calibration and cross-validation.

S4.2.2.1 Model calibration

The model equations were solved using the nominal parameters and the initial conditions $C_b(0) = 2$ (g/l) and $C_s(0) = 30$ (g/l) for the time interval $t \in [0, 12]$ hours. The observation functions were evaluated at time points $t_i = 2, 4, 6, 8, 10, 12$ to obtain their nominal values. Then random numbers were added to the nominal values to simulate the measurement error. The distributions were taken as Gaussian with zero mean and the following standard deviation: $\sigma_{ij} = 0.1 g_j(t_i) + 0.1$, aproximately resulting in a constant noise to signal ratio (proportional error) with a threshold at 0.1, i.e. the signals smaller than 0.1 cannot be decomposed from the measurement noise. Small negative values were corrected by taking their absolute value. To obtain N calibration datasets with different noise realization, the procedure was repeated N times.

S4.2.2.2 Model cross-validation

To obtain data for model cross-validation the same procedure described above was used, but the initial conditions of the states were also randomly chosen from a meaningful range. The exact values can be found in Additional File 2. Ten datasets were generated for model cross-validation.

S4.2.3 Calibration

All the 4 model parameters were estimated in this case study. The bounds of the parameters for the optimization are given in Table S4.2.1.

S4.3 FitzHugh-Nagumo model (FHN)

S4.3.1 Mathematical model

The FitzHugh-Nagumo model, as presented in [2, 3], describes the voltage (V)–current (R) relationship across an exon membrane. The model equations with one observable are:

$$\begin{aligned} \frac{dV}{dt} &= \gamma(V - V^3/3 + R) \\ \frac{dR}{dt} &= -1/\gamma(V - \alpha + \beta R) \\ g_1(t) &= V(t) \end{aligned} \tag{S4.3.2}$$

The model parameters to be estimated, are listed in Table S4.3.2.

Table S4.3.2: FitzHugh-Nagumo model parameters to be estimated: nominal values and bounds.

Par. id	Nominal	UB	LB
α	0.2	10^5	10^{-5}
β	0.2	10^5	10^{-5}
γ	3	10^5	10^{-5}

S4.3.2 Experimental conditions

S4.3.2.1 Calibration

The model equations were solved using the nominal parameters, the initial conditions $V(0) = -1$ and $R(0) = 1$ for the time interval $t \in [0, 20]$ unit. The observation function was evaluated at 6 time points equidistantly as $t = \text{linspace}(1, 20, 6)$ to obtain its nominal values. Then the experimental data was generated similarly as in the first case study, with a standard deviation of 10% of the nominal signal level, and a detection threshold for the observable of 0.1. This procedure was used to generate 6 data points for each of the 10 model calibration problems.

S4.3.2.2 Cross-validation

We followed the same procedure as for the model calibration, but the initial conditions of the states were randomly changed inside a meaningful range.

S4.3.3 Calibration

In model calibration, all the 3 model parameters were estimated. The parameter bounds for the optimization algorithm can be found in Table S4.3.2.

S4.4 Kholodenko MAPK signalling pathway (MAPK)

S4.4.1 Mathematical model

This case study considers the MAPK signalling pathway model originally presented in [4]. This model is also available from the Biomodels database [5] (BIOMD0000000010 - Kholodenko2000 - Ultrasensitivity and negative feedback bring oscillations in MAPK cascade). The model equations are

$$R_{\mathrm{J0}} = J0_{\mathrm{V1}} \frac{x_1}{(1 + (x_8/J0_{\mathrm{Ki}})^{J0_{\mathrm{n}}})(J0_{\mathrm{K1}} + x_1)}$$

$$R_{\mathrm{J1}} = J1_{\mathrm{V2}} \frac{x_2}{J1_{\mathrm{KK2}} + x_2} \qquad \frac{dx_1}{dt} = -R_{\mathrm{J0}} + R_{\mathrm{J1}}$$

$$R_{\mathrm{J2}} = J2_{\mathrm{k3}} \frac{x_2 x_3}{J2_{\mathrm{KK3}} + x_3} \qquad \frac{dx_2}{dt} = R_{\mathrm{J0}} - R_{\mathrm{J1}}$$

$$R_{\mathrm{J3}} = J3_{\mathrm{k4}} \frac{x_2 x_4}{J3_{\mathrm{KK4}} + x_4} \qquad \frac{dx_3}{dt} = -R_{\mathrm{J2}} + R_{\mathrm{J5}}$$

$$R_{\mathrm{J4}} = J4_{\mathrm{V5}} \frac{x_5}{J4_{\mathrm{KK5}} + x_5} \qquad \frac{dx_4}{dt} = R_{\mathrm{J2}} - R_{\mathrm{J3}} + R_{\mathrm{J4}} - R_{\mathrm{J5}}$$

$$R_{\mathrm{J5}} = J5_{\mathrm{V6}} \frac{x_4}{J5_{\mathrm{KK6}} + x_4} \qquad \frac{dx_5}{dt} = R_{\mathrm{J3}} - R_{\mathrm{J4}}$$

$$R_{\mathrm{J6}} = J6_{\mathrm{k7}} \frac{x_5 x_6}{J6_{\mathrm{KK7}} + x_6} \qquad \frac{dx_6}{dt} = -R_{\mathrm{J6}} + R_{\mathrm{J9}}$$

$$R_{\mathrm{J7}} = J7_{\mathrm{k8}} \frac{x_5 x_7}{J7_{\mathrm{KK8}} + x_7} \qquad \frac{dx_7}{dt} = R_{\mathrm{J6}} - R_{\mathrm{J7}} + R_{\mathrm{J8}} - R_{\mathrm{J9}}$$

$$R_{\mathrm{J8}} = J8_{\mathrm{V9}} \frac{x_8}{J8_{\mathrm{KK9}} + x_8} \qquad \frac{dx_8}{dt} = R_{\mathrm{J7}} - R_{\mathrm{J8}},$$

$$R_{\mathrm{J9}} = J9_{\mathrm{V10}} \frac{x_7}{J9_{\mathrm{KK10}} + x_7}$$

where the state variable x_1, $x_2 \ldots x_8$ denote the concentration of species Mos, Mos-P, Mek1, MKK-P, Mek1-PP, Erk2, Erk2-P, Erk2-PP, respectively. The model parameters are collected in Table S4.4.3. It is assumed that only the state variables x_2 (Mos-P) and x_7 (Erk2-P) can be measured in the experiments.

S4.4.2 Experimental conditions

S4.4.2.1 Calibration

The model equations were solved using the nominal parameters, and the initial conditions $x(0) = [90, 10, 280, 10, 10, 280, 10, 10]^T$ for the time interval $t \in [0, 1000]$ (arbitrary units). The two observation functions were evaluated at 10 time points for $t_i = [50, 100, 150, 200, 300, 400, 500, 600, 800, 1000]$ to obtain the nominal values. Psuedo-experimental data were generated as in the first case study, with a standard deviation of 10% of the nominal signal level and a detection threshold of 0.5. This procedure generated 20 data points (2 observables and 10 time points per observable) for model calibration.

S4.4.2.2 Cross-validation

For the model cross-validation we generated the data similarly as for the model calibration, but the initial conditions of the states were randomly changed within a meaningful range. The exact values used can be found in Additional File 2.

Table S4.4.3: Kholodenko MAPK Signalling pathway model parameters. The parameters for which the lower bounds (LB) and upper bounds (UB) are given are the estimated parameters. The other parameters are fixed at their nominal values.

Param. id.	Nominal	LB	UB
J0_V1	2.5	50	0.01
J0_Ki	9		
J0_n	1		
J0_K1	10		
J1_V2	0.25	50	0.01
J1_KK2	8		
J2_k3	0.025		
J2_KK3	15		
J3_k4	0.025		
J3_KK4	15		
J4_V5	0.75	50	0.01
J4_KK5	15		
J5_V6	0.75	50	0.01
J5_KK6	15		
J6_k7	0.025		
J6_KK7	15		
J7_k8	0.025		
J7_KK8	15		
J8_V9	0.5	50	0.01
J8_KK9	15		
J9_V10	0.5	50	0.01
J9_KK10	15		

S4.4.3 Calibration

In the model calibration procedure 6 model parameters were estimated. The parameter bounds for the optimization algorithm can be found in Table S4.3.2. The estimated parameters are the ones for which the bounds are given in the table.

S4.5 Goodwin oscillator model (GOsc)

S4.5.1 Mathematical model

The Goodwin oscillator [6] is one of the simplest models of oscillatory genetic networks (see Figure S4.5.2). In its original form, three state variables x_1, x_2 and x_3 describe RNA, protein and an end product concentrations. The model equations with two observables can be stated as

$$\begin{aligned}
\frac{dx_1}{dt} &= k_1 K_i^n/(K_i^n + x_3^n) - k_2 x_1 \\
\frac{dx_2}{dt} &= k_3 x_1 - k_4 x_2 \\
\frac{dx_3}{dt} &= k_5 x_2 - k_6 x_3 \\
g_1(t) &= x_1(t) \\
g_2(t) &= x_3(t)
\end{aligned} \tag{S4.5.3}$$

where we assumed that the RNA level and the end product concentration can be measured. The model parameters are given in Table S4.5.4

S4.5.2 Experimental conditions

S4.5.2.1 Calibration

The model equations were solved using nominal parameters and initial conditions $x_1(0) = 0.1$, $x_2(0) = 0.2$, $x_3 = 2.5$ for the time interval $t \in [0,\ 240]$ units. The observation functions were evaluated at 10 time points equidistantly as $t = \text{linspace}(0, 240, 10)$ to obtain their nominal values. Pseudo-experimental data was generated as above with standard deviation 10% of the nominal signal level and detection thresholds of 0.003 and 0.1 for the two observables. This procedure was used to generate 20 data points for model calibration.

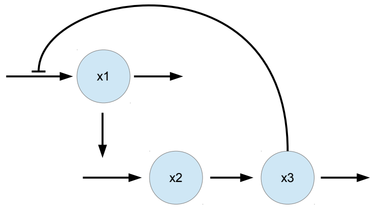

Figure S4.5.2: Schematic reaction scheme of Goodwin's oscillator.

Table S4.5.4: Goodwin oscillator: model parameters, nominal values and optimization bounds.

Par. id	Nominal	UB	LB
k_1	1	1000	0.001
k_2	0.1	1000	0.001
k_3	1	1000	0.001
k_4	0.1	1000	0.001
k_5	1	1000	0.001
k_6	0.1	1000	0.001
K_i	1	1000	0.001
n	10	12	1

S4.5.2.2 Cross-validation

For model cross-validation, we generated pseudo-data similarly as for model calibration, but the initial conditions of the states were randomly changed inside a meaningful range. The exact values used are given in Additional File 2.

S4.5.3 Calibration

All seven parameters were estimated in the model calibration procedure. The parameter bounds for the optimization algorithm can be found in Table S4.5.4. Although the model is small, its oscillatory nature results in objective functions with many local minima.

S4.6 TGF– β signalling pathway model (TGFB)

S4.6.1 Mathematical model

Geier and co-authors [7] presented a tutorial paper on parameter estimation in kinetic models where this TGF-β signalling pathway model [8] was used as a

case study. The dynamic model equations are

$$r_1 = k_1 C_{\text{TGFb\_TGFbR}}$$
$$r_2 = k_2 C_{\text{TGFbR}} C_{\text{TGFb}}$$
$$r_3 = k_3 C_{\text{TGFb\_TGFbR}} (1 - e^{-(\frac{t-k_{20}}{k_{21}})^{10}})$$
$$r_4 = k_4 C_{\text{TGFb\_TGFbR\_P}}$$
$$r_5 = k_5 C_{\text{TGFb\_TGFbR\_P}} C_{\text{I\_Smad}}$$
$$r_6 = k_6 C_{\text{I\_Smad\_TGFb\_TGFbR\_P}}$$
$$r_7 = k_7 C_{\text{Smad}} C_{\text{TGFb\_TGFbR\_P}}$$
$$r_8 = k_8 C_{\text{Smad}}$$
$$r_9 = k_9 C_{\text{Smad\_N}}$$
$$r_{10} = k_{10} 2 C_{\text{Smad\_P}} C_{\text{Smad\_P}}$$
$$r_{11} = k_{11} C_{\text{Smad\_P\_Smad\_P}}$$
$$r_{12} = k_{10} C_{\text{Smad\_P}} C_{\text{CoSmad}}$$
$$r_{13} = k_{11} C_{\text{Smad\_P\_CoSmad}}$$
$$r_{14} = k_8 C_{\text{CoSmad}}$$
$$r_{15} = k_9 C_{\text{CoSmad\_N}}$$
$$r_{16} = k_{12} k_8 C_{\text{Smad\_P\_Smad\_P}}$$
$$r_{17} = k_8 C_{\text{Smad\_P}}$$
$$r_{18} = k_9 C_{\text{Smad\_P\_N}}$$
$$r_{19} = k_{12} k_8 C_{\text{Smad\_P\_CoSmad}}$$
$$r_{20} = k_{13} C_{\text{Smad\_P\_N}}$$
$$r_{21} = k_{10} 2 C_{\text{Smad\_P\_N}} C_{\text{Smad\_P\_N}}$$
$$r_{22} = k_{11} C_{\text{Smad\_P\_Smad\_P\_N}}$$
$$r_{23} = k_{10} C_{\text{Smad\_P\_N}} C_{\text{CoSmad\_N}}$$
$$r_{24} = k_{11} C_{\text{Smad\_P\_CoSmad\_N}}$$
$$r_{25} = k_{14} \frac{C^2_{\text{Smad\_P\_CoSmad\_N}}}{C^2_{\text{Smad\_P\_CoSmad\_N}} + k^2_{15}}$$
$$r_{26} = k_{16} C_{\text{I\_Smad\_mRNA1}}$$
$$r_{27} = k_{17} C_{\text{I\_Smad\_mRNA2}}$$
$$r_{28} = k_{18} C_{\text{I\_Smad\_mRNA2}}$$
$$r_{29} = k_{19} C_{\text{I\_Smad}}$$

$$\frac{dC_{\text{TGFb}}}{dt} = r_1 - r_2$$
$$\frac{dC_{\text{dGFbR}}}{dt} = r_1 - r_2$$
$$\frac{dC_{\text{TGFb\_TGFbR}}}{dt} = -r_1 + r_2 - r_3 + r_4 + r_6$$
$$\frac{dC_{\text{TGFb\_TGFbR\_P}}}{dt} = r_3 - r_4 - r_5$$
$$\frac{dC_{\text{I\_Smad\_TGFb\_TGFbR\_P}}}{dt} = r_5 - r_6$$
$$\frac{dC_{\text{Smad}}}{dt} = -r_7 - r_8 + r_9$$
$$\frac{dC_{\text{Smad\_P}}}{dt} = r_7 - r_{10} + r_{11} - r_{12} + r_{13} - r_{17} + r_{18}$$
$$\frac{dC_{\text{CoSmad}}}{dt} = -r_{12} + r_{13} - r_{14} + r_{15}$$
$$\frac{dC_{\text{Smad\_P\_Smad\_P}}}{dt} = r_{10} - r_{11} - r_{16}$$
$$\frac{dC_{\text{Smad\_P\_CoSmad}}}{dt} = r_{12} - r_{13} - r_{19}$$
$$\frac{dC_{\text{Smad\_N}}}{dt} = r_8 - r_9 + r_{20}$$
$$\frac{dC_{\text{Smad\_P\_Smad\_P\_N}}}{dt} = r_{16} + r_{21} - r_{22}$$
$$\frac{dC_{\text{Smad\_P\_N}}}{dt} = r_{17} - r_{18} - r_{20} - r_{21} + r_{22} - r_{23} + r_{24}$$
$$\frac{dC_{\text{Smad\_P\_CoSmad\_N}}}{dt} = r_{19} + r_{23} - r_{24}$$
$$\frac{dC_{\text{CoSmad\_N}}}{dt} = r_{14} - r_{15} - r_{23} + r_{24}$$
$$\frac{dC_{\text{I\_Smad\_mRNA1}}}{dt} = r_{25} - r_{26}$$
$$\frac{dC_{\text{I\_Smad\_mRNA2}}}{dt} = r_{26} - r_{27}$$
$$\frac{dC_{\text{I\_Smad}}}{dt} = r_{28} - r_{29} - r_5 + r_6.$$

As in [7], we also assume that all the concentrations, except the Smad RNAs ($C_{\text{I\_Smad\_mRNA1}}$ and $C_{\text{I\_Smad\_mRNA2}}$), can be observed in the experiments. The model parameters can be found in Table S4.6.5.

S4.6.2 Experimental conditions

Following the procedure described in [7], the initial conditions of the dynamic state variables were determined by finding their steady states. For this calculation, we took $C_{\text{dGFbR}}(0) = 1$, $C_{\text{Smad}}(0) = 60$ and $C_{\text{CoSmad}}(0) = 60$, the initial concentrations of the other species as zero, and $k_3 = 0$ to temporarily remove the stimuli from the model. Then, simulations were performed for a suitable long time to obtain the steady state values of the variables. Finally, the value of C_{TGFb} was set to 1.0 and the nominal value (0.01) of k_3 was re-set.

Table S4.6.5: TGF–β signalling pathway parameters

Par. id	Nominal	UB	LB
k_1	0.00015	0.1	10^{-6}
k_2	0.023	1	0.0001
k_3	0.01	not estimated	
k_4	0.01	1	10^{-6}
k_5	0.01	1	0.0001
k_6	0.1	1	10^{-6}
k_7	0.000404	1	10^{-6}
k_8	0.0026	1	10^{-5}
k_9	0.0056	1	10^{-5}
k_{10}	0.002	1	10^{-6}
k_{11}	0.016	1	10^{-5}
k_{12}	5.7	100	0.1
k_{13}	0.00657	1	10^{-5}
k_{14}	0.0017	1	10^{-5}
k_{15}	1	100	0.001
k_{16}	0.0008	0.1	10^{-5}
k_{17}	0.001	0.1	10^{-5}
k_{18}	0.0021	0.1	10^{-5}
k_{19}	0.001	0.1	10^{-5}
k_{20}	9000	not estimated	
k_{21}	1800		

The numerical values of the steady state initial condition can be seen in Table S4.6.6.

The model equations were solved using the nominal parameters and the nominal initial conditions for the time interval $t \in [0, 18000]$ seconds. The observation functions were evaluated at 15 time points equidistantly as $t = \text{linspace}(0, 18000, 15)$ to obtain their nominal values. Pseudo-experimental data was generated using a standard deviation of 10% of the nominal signal level, while the detection thresholds for each observable was set to approximately 1% of their maximum level. This procedure generated 240 data points (16 observables, 15 time points per observable) for the model calibration.

S4.6.2.1 Cross-validation

We generated the data as in the previous subsection but the initial conditions of the states were randomly changed inside a meaningful range. The stimuli duration and initiation time parameters (k_{20} and k_{21}) were also randomly changed to generate 10 datasets for model cross-validation.

S4.6.3 Calibration

We used the same bounds on the parameters as reported in [7]. Note that parameters k_3, k_{20} and k_{21} are related to the Smad inhibition stimuli and they are not estimated. Parameter k_3 determines the strength of the inhibitor, while k_{20} and k_{21} respectively controls the appearance and duration of the inhibition.

Table S4.6.6: Nominal initial conditions for the TGF-β Pathway model

State name	Nominal initial condition
C_{TGFb}	1.0
C_{TGFbR}	1.0
$C_{\mathrm{TGFb\_TGFbR}}$	0.0
$C_{\mathrm{TGFb\_TGFbR_P}}$	0.0
$C_{\mathrm{I\_Smad\_TGFb\_TGFbR\_P}}$	0.0
C_{Smad}	40.98
$C_{\mathrm{Smad_P}}$	0.0
C_{CoSmad}	34.15
$C_{\mathrm{Smad\_P\_Smad\_P}}$	0.0
$C_{\mathrm{Smad\_P\_CoSmad}}$	0.0
$C_{\mathrm{Smad\_N}}$	19.02
$C_{\mathrm{Smad\_P\_Smad\_P\_N}}$	0.0
$C_{\mathrm{Smad\_P\_N}}$	0.0
$C_{\mathrm{Smad\_P\_CoSmad\_N}}$	0.0
$C_{\mathrm{CoSmad\_N}}$	15.85
$C_{\mathrm{I\_Smad\_mRNA1}}$	0.0
$C_{\mathrm{I\_Smad\_mRNA2}}$	0.0
$C_{\mathrm{I\_Smad}}$	0.0

S4.7 Three-steps Metabolic Pathway (TSMP)

S4.7.1 Mathematical model

This model describes a simple pathway with three enzymatic steps, as described in Moles et al[9]. The scheme of the pathway is shown in Figure S4.7.3.

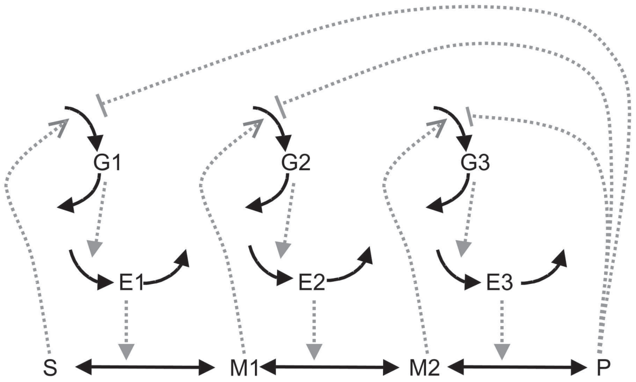

Figure S4.7.3: Three step pathway. S and P are the pathway substrate and product; M_1 and M_2 are intermediate metabolites of the pathway; E_1, E_2, and E_3 are the enzymes; G_1, G_2, and G_3 are the mRNA species for the enzymes, as described in [9]

The dynamics are given by the following system of differential equations:

$$\dot{G_1} = \frac{V_1}{1 + (\frac{P}{Ki_1})^{ni_1} + (\frac{Ka_1}{S})^{na_1}} - k_1 G_1$$
$$\dot{G_2} = \frac{V_2}{1 + (\frac{P}{Ki_2})^{ni_2} + (\frac{Ka_2}{M_1})^{na_2}} - k_2 G_2$$
$$\dot{G_3} = \frac{V_3}{1 + (\frac{P}{Ki_3})^{ni_3} + (\frac{Ka_3}{M_2})^{na_3}} - k_3 G_3$$
$$\dot{E_1} = \frac{V_4 G_1}{K_4 + G_1} - k_4 E_1$$
$$\dot{E_2} = \frac{V_5 G_2}{K_5 + G_2} - k_5 E_2$$
$$\dot{E_3} = \frac{V_6 G_3}{K_6 + G_3} - k_6 E_3$$
$$\dot{M_1} = \frac{kcat_1 E_1 (\frac{1}{Km_1})(S - M_1)}{1 + \frac{S}{Km_1} + \frac{M1}{Km_2}} - \frac{kcat_2 E_2 \frac{1}{Km_3}(M_1 - M_2)}{1 + \frac{M_1}{Km_3} + \frac{M_2}{Km_4}}$$
$$\dot{M_2} = \frac{kcat_2 E_2 \frac{1}{Km_3}(M_1 - M_2)}{1 + \frac{M_1}{Km_3} + \frac{M_2}{Km_4}} - \frac{kcat_3 E_3 \frac{1}{Km_5}(M_2 - P)}{1 + \frac{M_2}{Km_5} + \frac{P}{Km_6}}$$

S4.7.2 Experimental condition

S4.7.2.1 Calibration

Here we considered a variant with eight different experimental conditions, defined by different constant levels of substrate (S) and product (P), as given in the lower part of Table S4.7.7 (indicated by calib_exp1 – calib_exp8). The model equations with the given stimuli levels were solved using the nominal parame-

ters, and the initial conditions in Table S4.7.7 for the time interval $t \in [0, 120]$. The observation functions were evaluated for each experiment at 21 time points $t = \text{linspace}(0, 120, 21)$ to obtain the nominal values. Pseudo-experimental data was generated using Gaussian random errors with standard deviation of 10% of the nominal signal level. The detection thresholds for each observable was set to 2% of their average nominal values. This procedure resulted in 1344 data points (8 experiments, 8 observables and 21 time points for each) for model calibration.

S4.7.2.2 Cross-validation

For model cross-validation we generated the data similarly as for model calibration, but changing the P and S levels. Their numerical values are listed in Table S4.7.7 (indicated by valid_exp1 – valid_exp8)

S4.7.3 Calibration

The calibrated parameters are listed in the top part of Table S4.7.7. Lower and upper bounds for each parameter are reported in Additional File 2.

Table S4.7.7: Nominal values of parameters and (S,P) values for the 8 experiments considering the 3-Steps Metabolic Pathway

par. name	value	par. name	value	par. name	value
V1	1.0	V3	1.0	V6	0.1
Ki1	1.0	Ki3	1.0	K6	1.0
ni1	2.0	ni3	2.0	k_6	0.1
Ka1	1.0	Ka3	1.0	kcat1	1.0
na1	2.0	na3	2.0	Km1	1.0
k_1	1.0	k_3	1.0	Km2	1.0
V2	1.0	V4	0.1	kcat2	1.0
Ki2	1.0	K4	1.0	Km3	1.0
ni2	2.0	k_4	0.1	Km4	1.0
Ka2	1.0	V5	0.1	kcat3	1.0
na2	2.0	K5	1.0	Km5	1.0
k_2	1.0	k_5	0.1	Km6	1.0
calibration inp.:	[S]	[P]	cross-validation inp.:	[S]	[P]
calib_exp. #1	0.1	0.05	valid_exp. #1	0.1	0.13572
calib_exp. #2	0.1	1.0	valid_exp. #2	0.1	0.3684
calib_exp. #3	0.464	0.13572	valid_exp. #3	0.464	0.05
calib_exp. #4	0.464	1.0	valid_exp. #4	0.464	0.3684
calib_exp. #5	2.15	0.05	valid_exp. #5	2.15	0.13572
calib_exp. #6	2.15	0.3684	valid_exp. #6	2.15	1.0
calib_exp. #7	10.0	0.3684	valid_exp. #7	10.0	0.05
calib_exp. #8	10.0	1.0	valid_exp. #8	10.0	0.13572
States	initial cond.	States	initial cond.		
G1	0.6667	E2	0.3641		
G2	0.5725	E3	0.2946		
G3	0.4176	M1	1.419		
E1	0.4	M2	0.9346		

S4.8 Chemotaxis Pathway model (CHM)

S4.8.1 Mathematical model

This case study is based on the bacterial chemotaxis model by Bray et al [10]. The model describes the short term (without adaptation) bacterial response to aspartate (Asp) and nickel (Ni^{2+}) ion stimulus. The Asp binding to a transmembrane protein complex initiates an intracellular phopshorylation cascade, which changes the rotational behaviour of the flagellar motor and thus the swimming behaviour of the bacterium (see the reaction scheme in Figure S4.8.4). The mathematical model is available in the Biomodels Database (BIOMD0000000404 - Bray1993_chemotaxis).

The definitions of the reactions and the balance equations for the chemical species are as follows. The names of the chemical species encoded by the state variables can be found in Table S4.8.9. Apart from the swimming behaviour of the bacteria, we further assume that some phosphorylated species (listed in the same table) can be observed. The nominal values of the parameters are listed in Table S4.8.8.

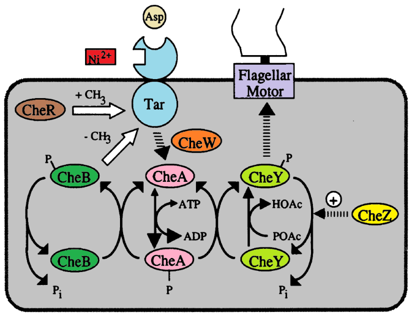

Figure S4.8.4: Chemotaxis pathway scheme adapted from [10]

$$
\begin{aligned}
rp\_r1 =& cell \cdot (rp\_r1\_kcat \cdot p2 \cdot x16) \\
rp\_r2 =& cell \cdot (x13 \cdot x16 \cdot rp\_r2\_kcat \cdot p2) \\
rp\_r3 =& cell \cdot (x15 \cdot x16 \cdot rp\_r3\_kcat \cdot p2) \\
rp\_r4 =& cell \cdot rp\_r4\_k1 \cdot x14 \cdot x22 \\
rp\_r5 =& cell \cdot rp\_r5\_k1 \cdot x17 \cdot x21 \\
rp\_r6 =& cell \cdot (rp\_r6\_kcat \cdot p2 \cdot x21) \\
rp\_r7 =& cell \cdot rp\_r7\_k1 \cdot x22 \\
rp\_r8 =& cell \cdot rp\_r8\_k1 \cdot x22 \cdot x20 \\
rp\_r9 =& cell \cdot rp\_r9\_k1 \cdot x17 \cdot x18 \\
rp\_r10 =& cell \cdot rp\_r10\_k1 \cdot x19 \\
rr\_r1 =& cell \cdot (rr\_r1\_k1 \cdot x2 \cdot asp - rr\_r1\_k2 \cdot x3) \\
rr\_r2 =& cell \cdot (rr\_r2\_k1 \cdot x2 \cdot ni - rr\_r2\_k2 \cdot x4) \\
rr\_r3 =& cell \cdot (rr\_r3\_k1 \cdot x2 \cdot x5 - rr\_r3\_k2 \cdot x6) \\
rr\_r4 =& cell \cdot (rr\_r4\_k1 \cdot x2 \cdot x16 - rr\_r4\_k2 \cdot x9) \\
rr\_r5 =& cell \cdot (rr\_r5\_k1 \cdot x5 \cdot x16 - rr\_r5\_k2 \cdot x12) \\
rr\_r6 =& cell \cdot (rr\_r6\_k1 \cdot x6 \cdot x16 - rr\_r6\_k2 \cdot x13) \\
rr\_r7 =& cell \cdot (rr\_r7\_k1 \cdot x9 \cdot x5 - rr\_r7\_k2 \cdot x13) \\
rr\_r8 =& cell \cdot (rr\_r8\_k1 \cdot x2 \cdot x12 - rr\_r8\_k2 \cdot x13) \\
rr\_r9 =& cell \cdot (rr\_r9\_k1 \cdot x3 \cdot x5 - rr\_r9\_k2 \cdot x7) \\
rr\_r10 =& cell \cdot (rr\_r10\_k1 \cdot x3 \cdot x16 - rr\_r10\_k2 \cdot x10) \\
rr\_r11 =& cell \cdot (rr\_r11\_k1 \cdot x7 \cdot x16 - rr\_r11\_k2 \cdot x14) \\
rr\_r12 =& cell \cdot (rr\_r12\_k1 \cdot x10 \cdot x5 - rr\_r12\_k2 \cdot x14) \\
rr\_r13 =& cell \cdot (rr\_r13\_k1 \cdot x3 \cdot x12 - rr\_r13\_k2 \cdot x14) \\
rr\_r14 =& cell \cdot (rr\_r14\_k1 \cdot x4 \cdot x5 - rr\_r14\_k2 \cdot x8) \\
rr\_r15 =& cell \cdot (rr\_r15\_k1 \cdot x4 \cdot x16 - rr\_r15\_k2 \cdot x11) \\
rr\_r16 =& cell \cdot (rr\_r16\_k1 \cdot x8 \cdot x16 - rr\_r16\_k2 \cdot x15) \\
rr\_r17 =& cell \cdot (rr\_r17\_k1 \cdot x11 \cdot x5 - rr\_r17\_k2 \cdot x15) \\
rr\_r18 =& cell \cdot (rr\_r18\_k1 \cdot x4 \cdot x12 - rr\_r18\_k2 \cdot x15) \\
rm\_r1 =& cell \cdot (ka \cdot (x23 \cdot x22 - \kappa/4 \cdot x24)/cell) \\
rm\_r2 =& cell \cdot (ka \cdot (x24 \cdot x22 - 2 \cdot \alpha \cdot \kappa/3 \cdot x25)/cell) \\
rm\_r3 =& cell \cdot (ka \cdot (x25 \cdot x22 - 3 \cdot \alpha \cdot \alpha \cdot \kappa/2 \cdot x26)/cell) \\
rm\_r4 =& cell \cdot (ka \cdot (x26 \cdot x22 - 4 \cdot \alpha \cdot \alpha \cdot \alpha \cdot \kappa \cdot x27)/cell) \\
rr\_1 =& cell \cdot (rr\_1\_k1 \cdot x9 \cdot asp - rr\_1\_k2 \cdot x10) \\
rr\_2 =& cell \cdot (rr\_2\_k1 \cdot x6 \cdot asp - rr\_2\_k2 \cdot x7) \\
rr\_3 =& cell \cdot (rr\_3\_k1 \cdot x13 \cdot asp - rr\_3\_k2 \cdot x14)
\end{aligned}
$$

$$\frac{dx2}{dt} = (1/cell) \cdot (-rr\_r1 - rr\_r2 - rr\_r3 - rr\_r4 - rr\_r8)$$
$$\frac{dx3}{dt} = (1/cell) \cdot (rr\_r1 - rr\_r9 - rr\_r10 - rr\_r13)$$
$$\frac{dx4}{dt} = (1/cell) \cdot (rr\_r2 - rr\_r14 - rr\_r15 - rr\_r18)$$
$$\frac{dx5}{dt} = (1/cell) \cdot (-rr\_r3 - rr\_r5 - rr\_r7 - rr\_r9 - rr\_r12 - rr\_r14 - rr\_r17)$$
$$\frac{dx6}{dt} = (1/cell) \cdot (rr\_r3 - rr\_r6 - rr\_2)$$
$$\frac{dx7}{dt} = (1/cell) \cdot (rr\_r9 - rr\_r11 + rr\_2)$$
$$\frac{dx8}{dt} = (1/cell) \cdot (rr\_r14 - rr\_r16)$$
$$\frac{dx9}{dt} = (1/cell) \cdot (rr\_r4 - rr\_r7 - rr\_1)$$
$$\frac{dx10}{dt} = (1/cell) \cdot (rr\_r10 - rr\_r12 + rr\_1)$$
$$\frac{dx11}{dt} = (1/cell) \cdot (rr\_r15 - rr\_r17)$$
$$\frac{dx12}{dt} = (1/cell) \cdot (rr\_r5 - rr\_r8 - rr\_r13 - rr\_r18)$$
$$\frac{dx13}{dt} = (1/cell) \cdot (rr\_r6 + rr\_r7 + rr\_r8 - rr\_3)$$
$$\frac{dx14}{dt} = (1/cell) \cdot (-rp\_r4 + rp\_r4 + rr\_r11 + rr\_r12 + rr\_r13 + rr\_3)$$
$$\frac{dx15}{dt} = (1/cell) \cdot (rr\_r16 + rr\_r17 + rr\_r18)$$
$$\frac{dx16}{dt} = (1/cell) \cdot (-rp\_r1 - rp\_r2 - rp\_r3 + rp\_r5 + rp\_r9 - rr\_r4 - rr\_r5 - rr\_r6 - rr\_r10 - rr\_r11 - rr\_r15 - rr\_r16)$$
$$\frac{dx17}{dt} = (1/cell) \cdot (rp\_r1 + rp\_r2 + rp\_r3 - rp\_r5 - rp\_r9)$$
$$\frac{dx18}{dt} = (1/cell) \cdot (-rp\_r9 + rp\_r10)$$
$$\frac{dx19}{dt} = (1/cell) \cdot (rp\_r9 - rp\_r10)$$
$$\frac{dx20}{dt} = (1/cell) \cdot (-rp\_r8 + rp\_r8)$$
$$\frac{dx21}{dt} = (1/cell) \cdot (rp\_r4 - rp\_r5 - rp\_r6 + rp\_r7 + rp\_r8)$$
$$\frac{dx22}{dt} = (1/cell) \cdot (-rp\_r4 + rp\_r5 + rp\_r6 - rp\_r7 - rp\_r8 - rm\_r1 - rm\_r2 - rm\_r3 - rm\_r4)$$
$$\frac{dx23}{dt} = (1/cell) \cdot (-rm\_r1)$$
$$\frac{dx24}{dt} = (1/cell) \cdot (rm\_r1 - rm\_r2)$$
$$\frac{dx25}{dt} = (1/cell) \cdot (rm\_r2 - rm\_r3)$$
$$\frac{dx26}{dt} = (1/cell) \cdot (rm\_r3 - rm\_r4)$$
$$\frac{dx27}{dt} = (1/cell) \cdot (rm\_r4)$$

S4.8.2 Experimental conditions

S4.8.2.1 Calibration

We considered two experimental conditions, each one corresponding to a different stepwise profile of the Asp level. The 3-step concentration profiles of

Table S4.8.8: Parameters of the chemotaxis pathway model

**Param.**	**Name**	**Nom. value**	**Param.**	**Name**	**Nom. value**
p1	rp_r1_kcat	0.001	p30	rr_r10_k2	1
p2	rp_r2_kcat	75000	p31	rr_r11_k1	400000
p3	rp_r3_kcat	200000	p32	rr_r11_k2	1
p4	rp_r4_k1	100000000	p33	rr_r12_k1	400000
p5	rp_r5_k1	200000	p34	rr_r12_k2	1
p6	rp_r6_kcat	0	p35	rr_r13_k1	400000
p7	rp_r7_k1	0.037	p36	rr_r13_k2	1
p8	rp_r8_k1	500000	p37	rr_r14_k1	0.1
p9	rp_r9_k1	1000000	p38	rr_r14_k2	1
p10	rp_r10_k1	1	p39	rr_r15_k1	0.01
p11	rr_r1_k1	1000000	p40	rr_r15_k2	1
p12	rr_r1_k2	1	p41	rr_r16_k1	0.4
p13	rr_r2_k1	1000	p42	rr_r16_k2	1
p14	rr_r2_k2	1	p43	rr_r17_k1	0.4
p15	rr_r3_k1	100000	p44	rr_r17_k2	1
p16	rr_r3_k2	1	p45	rr_r18_k1	0.4
p17	rr_r4_k1	10000	p46	rr_r18_k2	1
p18	rr_r4_k2	1	p47	rr_1_k1	1000000
p19	rr_r5_k1	100000	p48	rr_1_k2	1
p20	rr_r5_k2	1	p49	rr_2_k1	1000000
p21	rr_r6_k1	400000	p50	rr_2_k2	1
p22	rr_r6_k2	1	p51	rr_3_k1	1000000
p23	rr_r7_k1	400000	p52	rr_3_k2	1
p24	rr_r7_k2	1	p53	cell	1.41E-15
p25	rr_r8_k1	400000	p54	α	0.14
p26	rr_r8_k2	1	p55	κ	2.25E-07
p27	rr_r9_k1	100000	p56	ka	0.1
p28	rr_r9_k2	1	p57	ni	0
p29	rr_r10_k1	10000	p58	p2	0.997

Asp for these two experiments are given in the lower part of Table S4.8.9 (indicated by calib_exp1 and calib_exp2). The model equations with the given stimuli profiles were solved using the nominal parameters, and the initial conditions in Table S4.8.9 for the time interval $t \in [0, 180]$ seconds. The observation functions were evaluated at 10 time points in each experiment at time points $t = \mathrm{linspace}(0, 180, 10)$ to obtain the nominal values. Pseudo-experimental data was generated with standard deviation of 5% of the nominal signal level. Detection threshold for each observable was set to 2% of the maximum observation values. This procedure resulted in 160 data points (2 experiments, 8 observables and 10 time points for each) for model calibration.

S4.8.2.2 For cross-validation

For model cross-validation, we generated pseudo-data as above but with changed stimulus level. Their numerical values are listed in Table S4.8.9 (indicated by valid_exp1 and valid_exp2). Numerical values can be found in Additional File 2.

S4.8.3 Calibration

Before the model calibration a parameter sensitivity analysis was performed. This showed the lack of sensitivity of the outputs with respect to some parameters. These parameters belong to the Ni stimulus reactions and the corresponding part of the pathway. Since in the experiments considered no Ni stimulus is applied, these parameters are inactive and their values cannot be estimated.

The remaining 38 estimated parameters are p1, p2, p4, p5, p7-p12, p15-p36 and p47-p52 (see Table S4.8.8 for the naming convention). The lower and upper bounds of these parameters were set as $\mathrm{LB} = p_{\mathrm{nom}} \times 10^{-5}$ and $\mathrm{UB} = p_{\mathrm{nom}} \times 10^{5}$, where p_{nom} is the nominal value of the parameters.

Further, note that the parameters have very different ranges which would lead to numerical issues in the optimization. To avoid this, the parameters were scaled for the calibration, such that their range should overlap. The scaled values are reported in the Additional File 2.

Table S4.8.9: Initial values and input variables for the chemotaxis model

States	Name	Initial value	States	Name	Initial value
x2	T	3.12E-06	x15	Tni_WA	0
x3	Tasp	0	x16	A	3.00E-06
x4	Tni	0	x17	Ap	3.48E-08
x5	W	2.89E-06	x18	B	1.93E-06
x6	TW	5.91E-07	x19	Bp	6.87E-08
x7	Tasp_W	0	x20	Z	2.00E-05
x8	Tni_W	0	x21	Y	9.90E-06
x9	TA	4.44E-07	x22	Yp	7.00E-09
x10	Tasp_A	0	x23	M	6.24E-09
x11	Tni_A	0	x24	MYp	7.77E-10
x12	WA	6.78E-07	x25	MYpYp	2.99E-10
x13	TWA	8.47E-07	x26	MYpYpYp	3.78E-10
x14	Tasp_WA	0.00E+00	x27	MYpYpYpYp	2.31E-09
Input:	stepwise function of aspartate level				
	time interval(s):	[0–60]	[60–120]	[120–180]	
calib_exp1	[asp]	0	$3.33 \cdot 10^{-8}$	$6.60 \cdot 10^{-8}$	
calib_exp2	[asp]	$2 \cdot 10^{-7}$	$3.33 \cdot 10^{-8}$	10^{-7}	
valid_exp1	[asp]	0	10^{-7}	$2 \cdot 10^{-7}$	
valid_exp2	[asp]	$3 \cdot 10^{-7}$	$3.33 \cdot 10^{-8}$	$1.50 \cdot 10^{-7}$	
Observables:					
	[Ap], [Bp], [Yp], [MYp], [MYpYp], [MYpYpYp], [MYpYpYpYp]				
	$Bias = \frac{[M]+[MYp]}{[M]+[MYp]+[MYpYp]+[MYpYpYp]+[MYpYpYpYp]}$				

References

[1] Rodriguez-Fernandez, M., Kucherenko, S., Pantelides, C., Shah, N.: Optimal experimental design based on global sensitivity analysis. In: 17th European Symposium on Computer Aided Process Engineering, pp. 1–6 (2007).

[2] FitzHugh, R.: Impulses and physiological states in theoretical models of nerve membrane. Biophysical Journal **1**(6), 445–466 (1961)

[3] Nagumo, J., Arimoto, S., Yoshizawa, S.: An active pulse transmission line simulating nerve axon. Proceedings of the IRE **50**(10), 2061–2070 (1962)

[4] Kholodenko, B.N.: Cell-signalling dynamics in time and space. Molecular Cell Biology **7**(3), 165–176 (2006)

[5] Li, C., Donizelli, M., Rodriguez, N., Dharuri, H., Endler, L., Chelliah, V., Li, L., He, E., Henry, A., Stefan, M.I., Others: BioModels Database: An enhanced, curated and annotated resource for published quantitative kinetic models. BMC Systems Biology **4**(1), 92 (2010)

[6] Goodwin, B.C.: Oscillatory behavior in enzymatic control processes. Advances in Enzyme Regulation **3**, 425–438 (1965).

[7] Geier, F., Fengos, G., Felizzi, F., Iber, D.: Analyzing and Constraining Signaling Networks: Parameter Estimation for the User. In: Liu, X., Betterton, M.D. (eds.) Computational Modeling of Signaling Networks. Methods in Molecular Biology, vol. 880, pp. 23–40. Humana Press, Totowa, NJ (2012).

[8] Schmierer, B., Tournier, A.L., Bates, P.A., Hill, C.S.: Mathematical modeling identifies smad nucleocytoplasmic shuttling as a dynamic signal-interpreting system. Proceedings of the National Academy of Sciences **105**(18), 6608–6613 (2008)

[9] Moles, C.G., Mendes, P., Banga, J.R.: Parameter Estimation in Biochemical Pathways : A Comparison of Global Optimization Methods. Genome Research **13**, 2467–2474 (2003).

[10] Bray, D., Bourret, R.B., Simont, M.I.: Computer Simulation of the Phosphorylation Cascade Controlling Bacterial Chemotaxis. Molecular Biology of the Cell **4**(May), 469–482 (1993)
