# Supplementary material for: Robust and efficient parameter estimation in dynamic models of biological systems
Source: BMC Syst Biol. 2015 Oct 29;9:74. doi: 10.1186/s12918-015-0219-2 (PMC4625902; doi:10.1186/s12918-015-0219-2)
Supplement: Additional file 5 — Detailed results. This file contains detailed results of the presented method for all the case studies considered. (PDF 1133 kb) [file 12918_2015_219_MOESM5_ESM.pdf]

Additional File 5 for
Robust and Efficient Parameter Estimation in Dynamic Models of Biological Systems
Case studies - Detailed results

Attila Gábor and Julio R. Banga
IIM-CSIC. Eduardo Cabello 6, 36208, Vigo, Spain

October 19, 2015

S5.1 Summary

Here we present detailed results, in graphical format, for the case studies considered in the main text. For each case study the results are grouped into the following sets of figures:

1. **Bias-variance trade-off.** For each case study, we repeated model calibrations (with and without regularization) 10 times. The difference between these calibrations was only the noise realization in the data. In this way, the robustness of the solutions of the original and the regularized problems was tested with respect to small perturbation in the measurements. We present below the following plots:

   (a) **Bias-variance trade-off for the estimated parameters** versus the regularization parameter. These plots show for each regularization candidate the bias, the variance and the mean squared error between parameter estimates and the true parameters. In general a larger regularization parameter cause larger bias and smaller variance.

   (b) **Bias-variance trade-off for the calibrated predictions** as a function of the regularization parameter. These plots show for each regularization candidate the bias, the variance and the mean squared error between the nominal (error-free) trajectories and the estimated trajectories. In general a larger regularization parameter cause larger bias and smaller variance of the trajectories.

   (c) **Simulated model predictions together with the data**. First we report the estimated trajectories of the calibrated models *without using the regularization.* The differences among the trajectories are caused by the different calibration data. The range of the calibration data for each time point and observable is depicted as bars. Then the predictions of the calibrated models (with regularization) is shown. These trajectories generally have smaller deviation from each other (smaller variance) but slightly more bias from the nominal trajectory.

(d) **Effective number of calibration parameters**, intended to show the reduced complexity of the calibrated models due to regularization. The effective number of parameters also depends on the estimated parameters, therefore it is slightly different in each of the ten calibrations. The average and the standard deviation over the ten replicated calibration problems is depicted with error-bars.

2. **Tuning of the regularization**. We present the following figures:

   (a) **L-curve**, which depicts the results of the optimization algorithm for each regularization parameter (α_i). For each regularization parameter the optimal model fit ($\mathrm{Q}_{LS}(\hat{\theta}_{\alpha_i})$) and regularization penalty $\Gamma(\hat{\theta}_{\alpha_i})$ pairs are shown (points in the Pareto-front).

   (b) **Generalized cross validation (GCV) score** as a function of the regularization parameter. This curve estimates the cross-validation error of the calibrated model and its minimum is used to select the optimal regularization parameter.

3. **Multi-modality of the objective function and optimization convergence analysis.** First a histogram is presented which shows the multimodality of the objective function. Second, the convergence curves are reported for different optimization algorithms (and several runs per algorithm), which show how efficiently the calibration problem was solved. Finally, the histograms of the final values shows the robustness of the optimization algorithms for the case studies.

S5.2 Biomass batch growth (BBG)

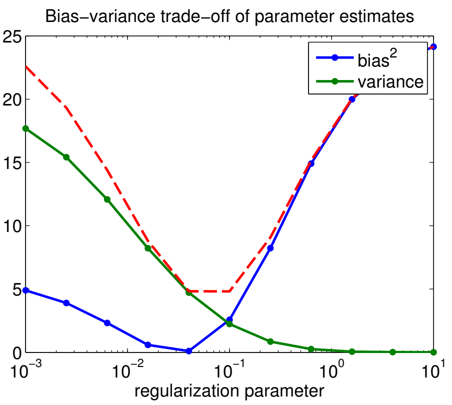

(a) Bias-variance trade-off for estimated parameters

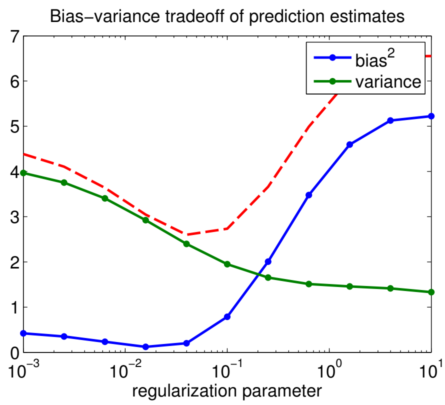

(b) Bias-variance trade-off for model prediction

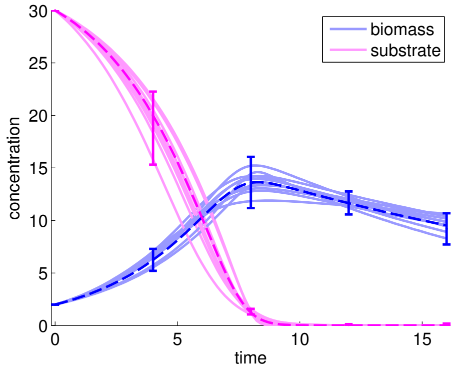

(c) Predictions of *non-regularized* model calibrations

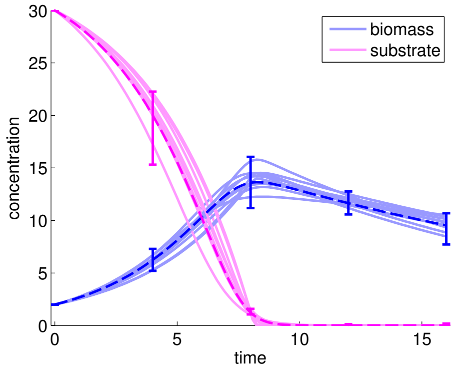

(d) Predictions of *regularized* model calibrations

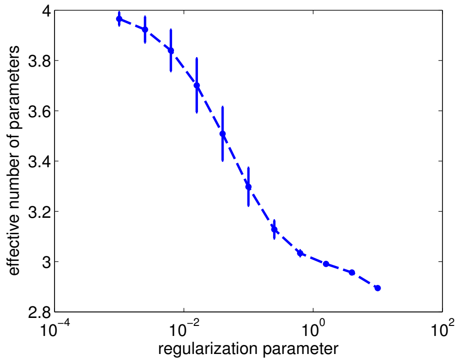

(e) Effective number of model parameters

Figure S5.2.1: Biomass growth model. (a,b) Bias-variance trade-off for model estimated parameters and model predictions. Further,(c,d) shows the simulated trajectories of the calibrated models with and without using the regularization and (e) the number of effective parameters versus the regularization parameter.

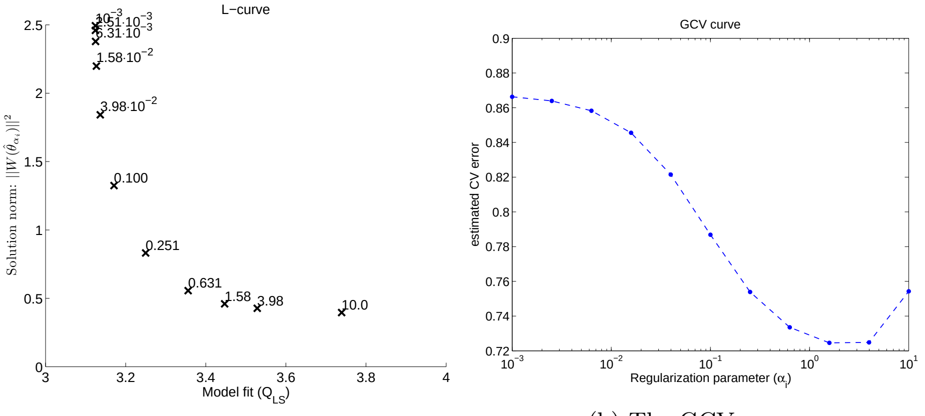

(a) The L-curve

(b) The GCV curve

Figure S5.2.2: Biomass growth model calibration. L-curve and generalized cross-validation curve showing the regularization candidates (values of regularization parameter). The selected regularization candidate corresponds to the minimum of the GCV curve.

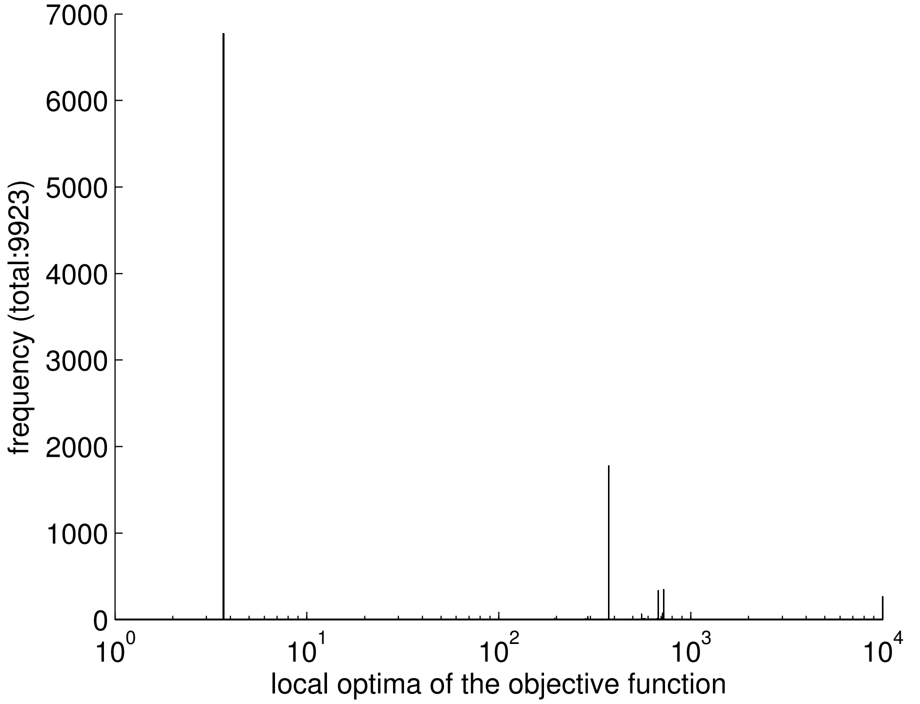

Figure S5.2.3: Case study BBG. Empirical distribution of the local optima, obtained by multi-start local optimization from random initial points (log LHS).

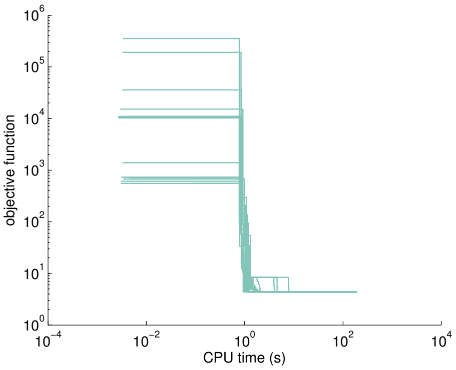

(a) Convergence of eSS2b with regularization

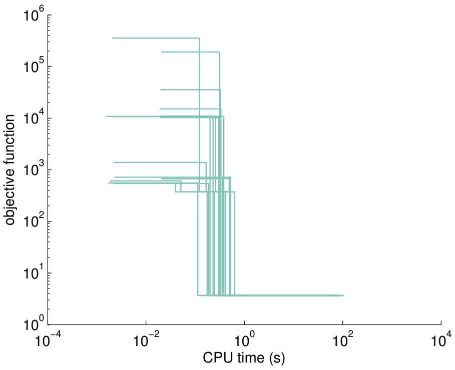

(b) Convergence of AMS

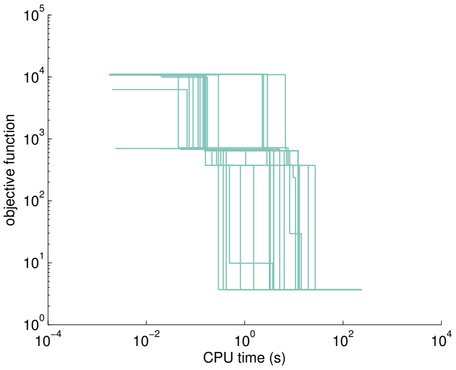

(c) Convergence of SMS

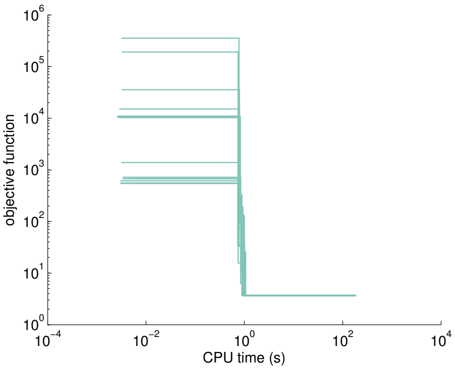

(d) Convergence of eSS2b

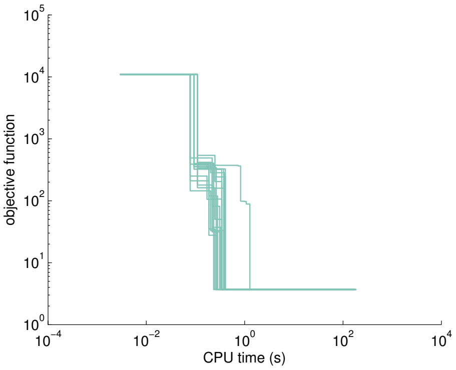

(e) Convergence of eSS2a

Figure S5.2.4: Convergence curves for the BBG case study. SMS: simple multi start of NL2SOL; AMS: multistart of NL2SOL with log Latin hypercube sampling, eSS2a: scatter search with NL2SOL and log initial guesses, eSS2b: scatter search with NL2SOL and log Latin hypercube initial guesses

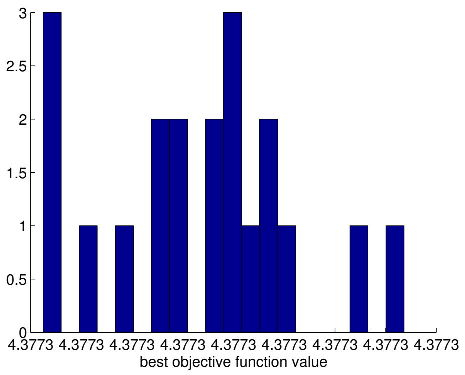

(a) Final values of eSS2b with regularization

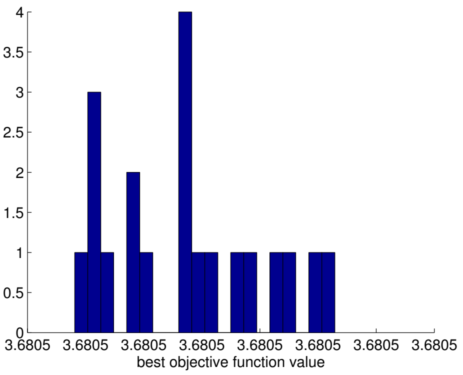

(b) Final values of AMS

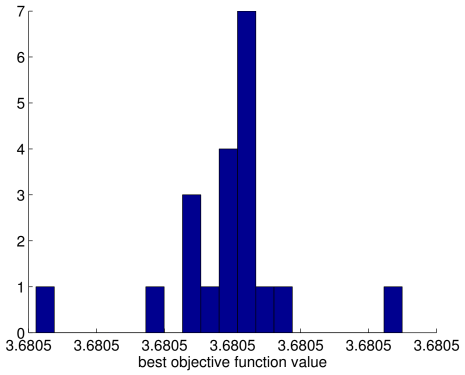

(c) Final values of SMS

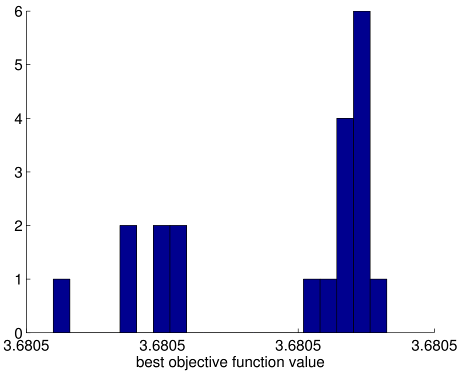

(d) Final values of eSS2b

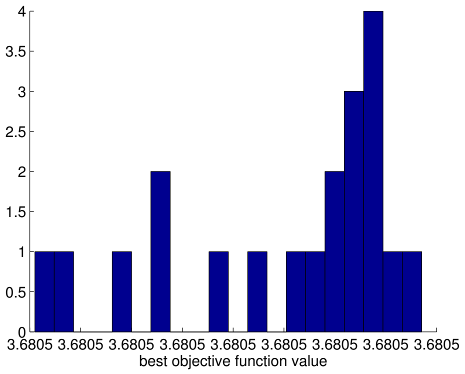

(e) Final values of eSS2a

Figure S5.2.5: BBG case study: distribution of the final objective function values with different optimization methods. SMS: simple multi start of NL2SOL; AMS: multistart of NL2SOL with logarithim Latin hypercube sampling, eSS2a: scatter search with NL2SOL and logarithmic initial guesses, eSS2b: scatter search with NL2SOL and logarithmic Latin hypercube initial guesses

S5.3 FitzHugh-Nagumo model (FHN)

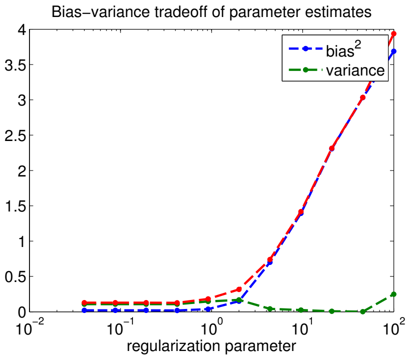

(a) Bias-variance trade-off for estimated parameters

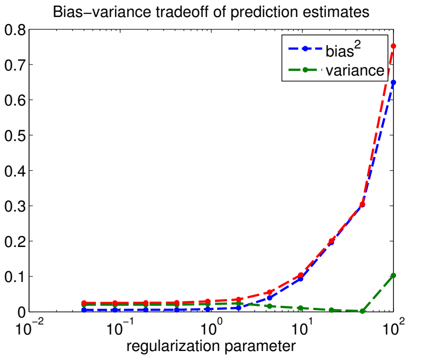

(b) Bias-variance trade-off for model prediction

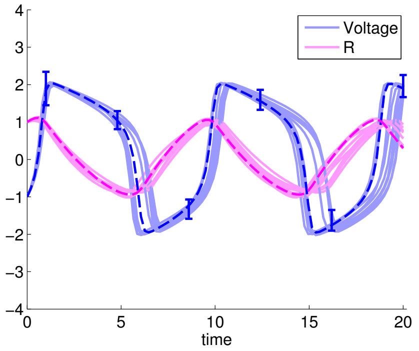

(c) Predictions of *non-regularized* model calibrations. Note, that only the Voltage is measured, but not the current R.

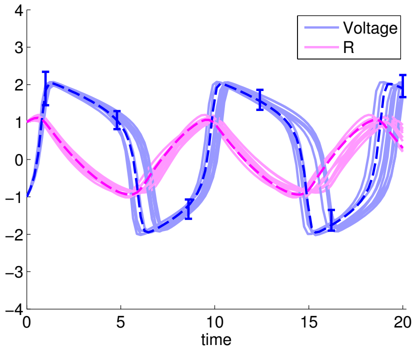

(d) Predictions of *regularized* model calibrations. Note, that only the Voltage is measured, but not the current R.

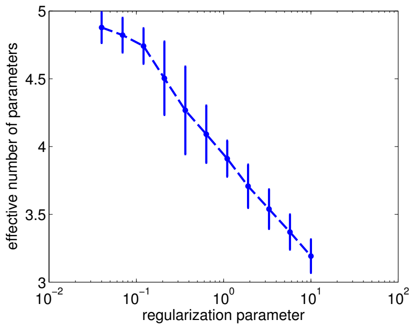

(e) Effective number of parameters

Figure S5.3.6: FitzHugh-Nagumo (FHN) case study: (a,b) bias-variance trade-off for model estimated parameters and model predictions. (c,d) simulated trajectories of the calibrated models with and without using the regularization. (e) effective number of parameters

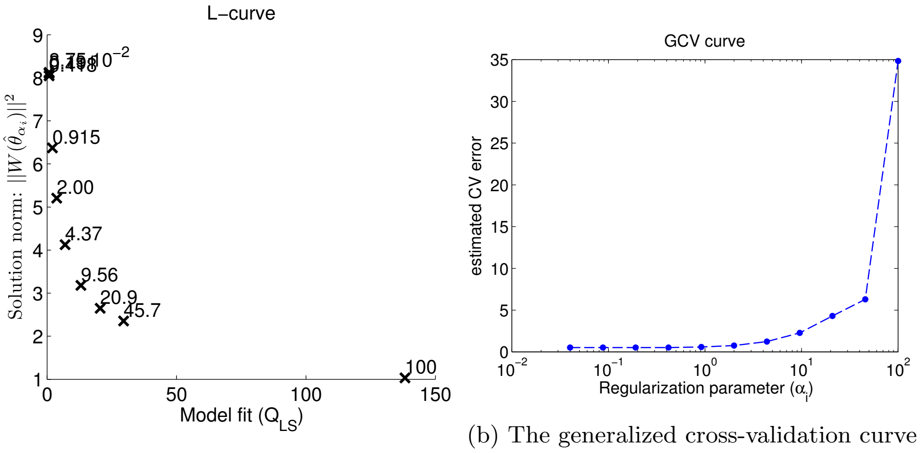

(a) The L-curve

(b) The generalized cross-validation curve

Figure S5.3.7: FitzHugh-Nagumo (FHN) case study: model calibration. L-curve and the generalized cross-validation curve. The selected regularization candidate corresponds to the minimum of the GCV curve.

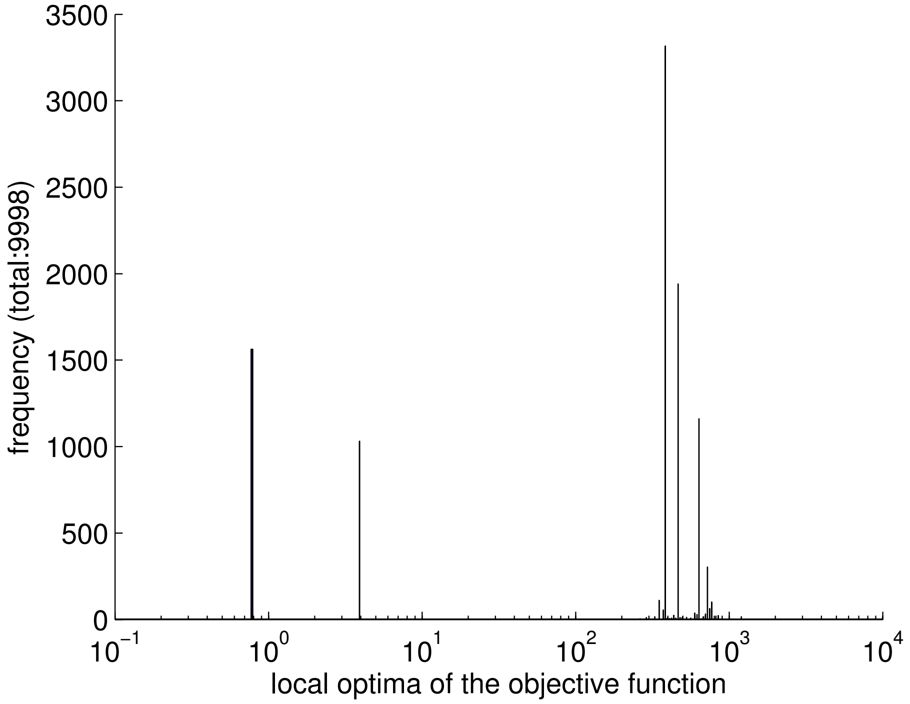

Figure S5.3.8: FitzHugh-Nagumo (FHN) case study: empirical distribution of the local optima, obtained by multi-start local optimization from random initial points (log LHS).

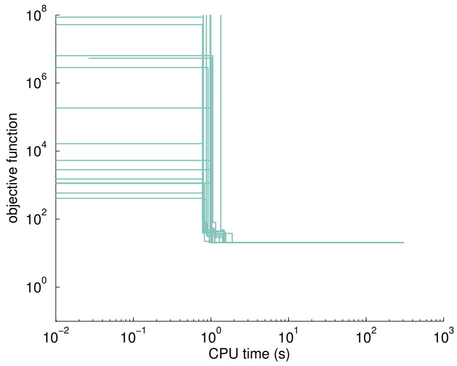

(a) Convergence of eSS2b with regularization

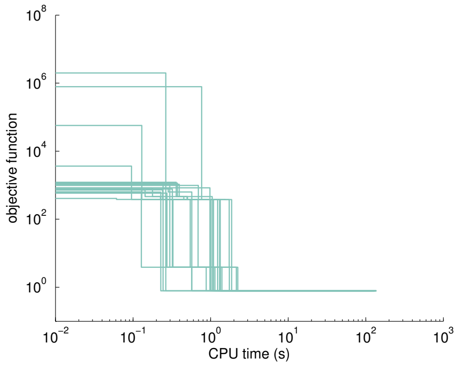

(b) Convergence of AMS

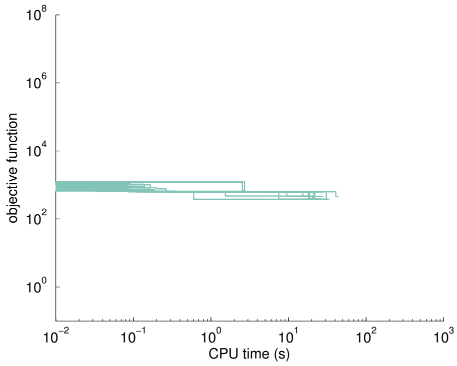

(c) Convergence with multistart n2fb, log LHS initial guesses

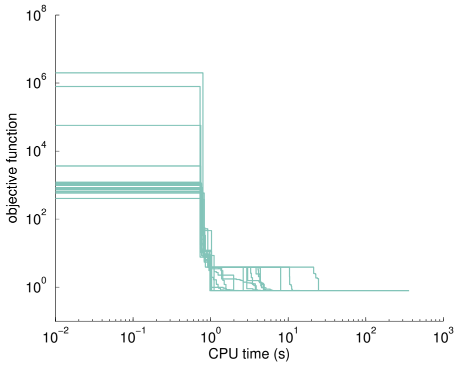

(d) Convergence of eSS2b

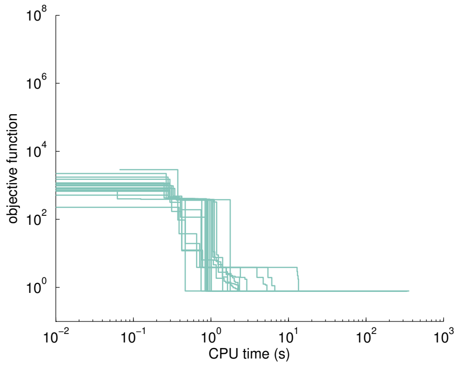

(e) Convergence of eSS2a

Figure S5.3.9: Convergence curves for the FHN case study

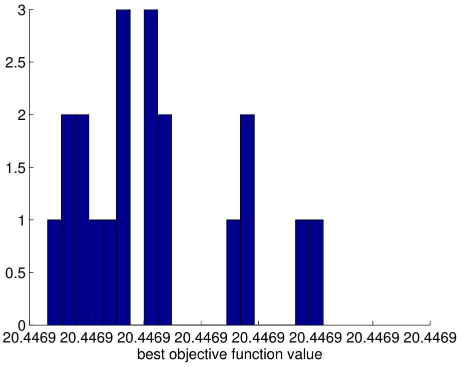

(a) Final values of eSS2b with regularization

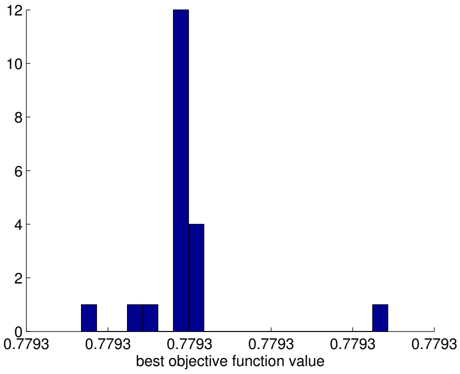

(b) Final values of AMS

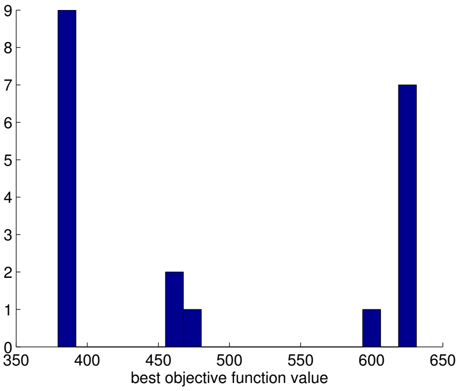

(c) Final values of SMS

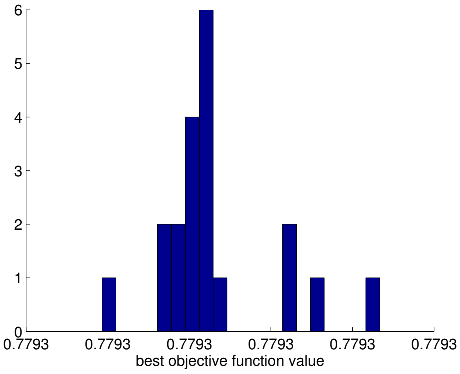

(d) Final values of eSS2b

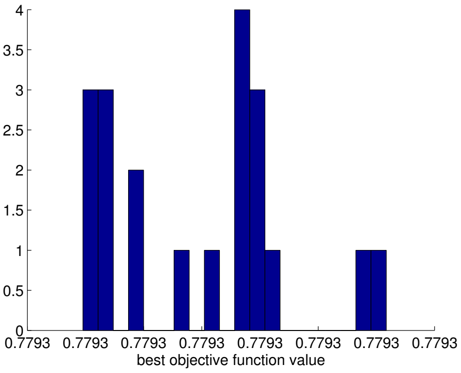

(e) Final values of eSS2a

Figure S5.3.10: FHN case study: distribution of the final objective function values. SMS: simple multi start of NL2SOL; AMS: multistart of NL2SOL with logarithim Latin hypercube sampling, eSS2a: scatter search with NL2SOL and logarithmic initial guesses, eSS2b: scatter search with NL2SOL and logarithmic Latin hypercube initial guesses

S5.4 Kholodenko MAPK signalling pathway (MAPK)

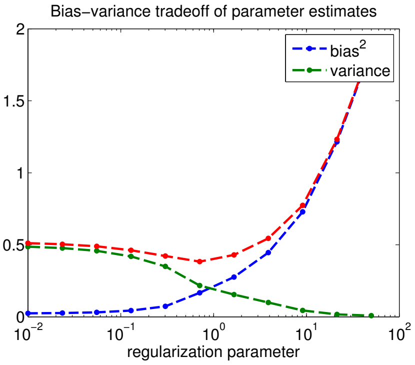

(a) Bias-variance trade-off for estimated parameters

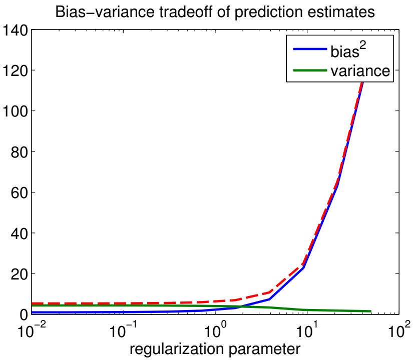

(b) Bias-variance trade-off for model prediction

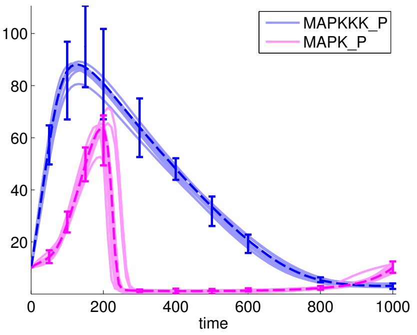

(c) Predictions of *non-regularized* model calibrations.

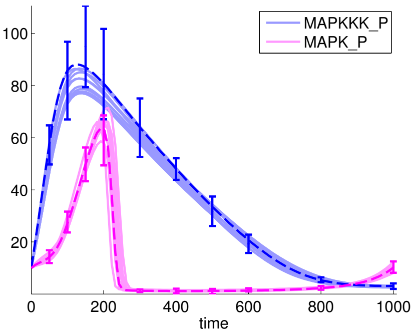

(d) Predictions of *regularized* model calibrations.

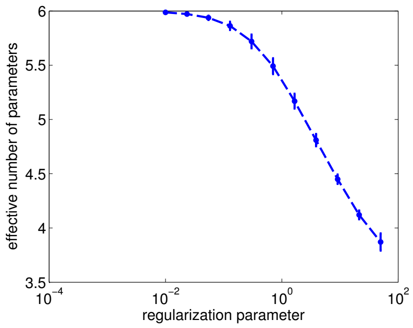

(e) Effective number of parameters

Figure S5.4.11: MAPK case study: (a,b) bias-variance trade-off for model estimated parameters and model predictions. (c,d) simulated trajectories of the calibrated models with and without regularization. (e) effective number of parameters

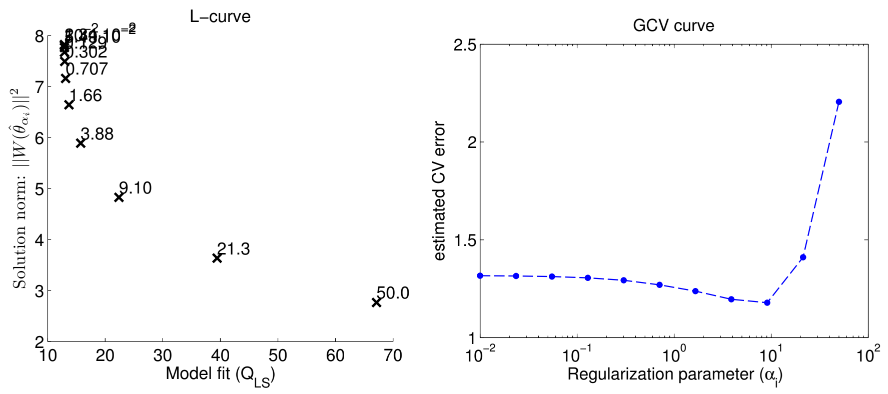

Figure S5.4.12: MAPK case study: L-curve and generalized cross-validation curve. The selected regularization candidate corresponds to the minimum of the GCV curve.

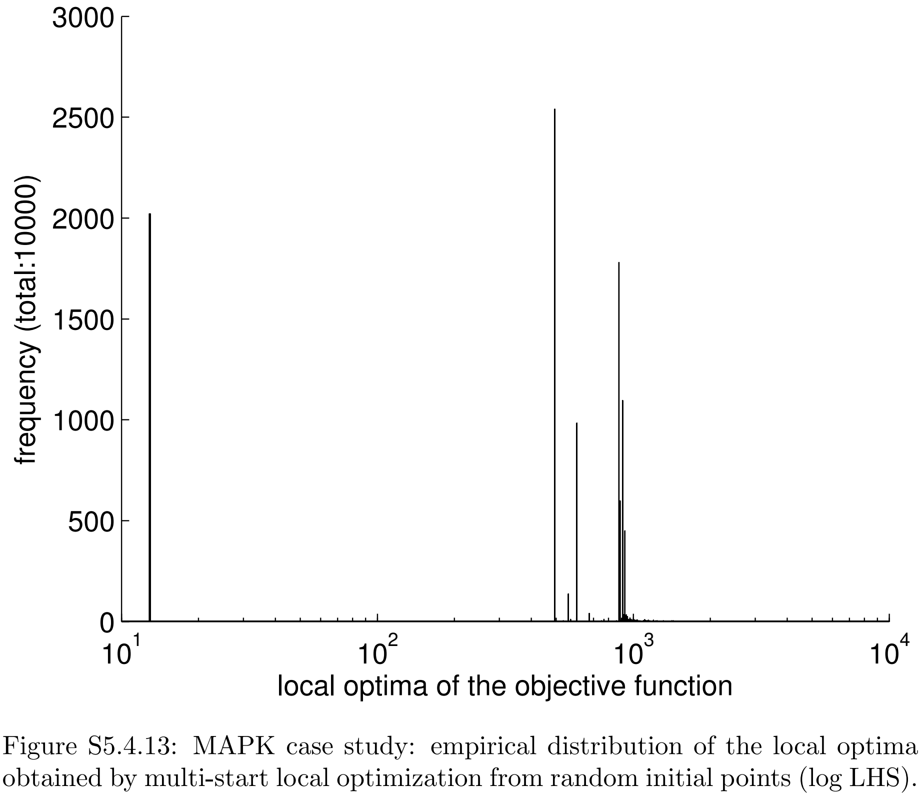

Figure S5.4.13: MAPK case study: empirical distribution of the local optima obtained by multi-start local optimization from random initial points (log LHS).

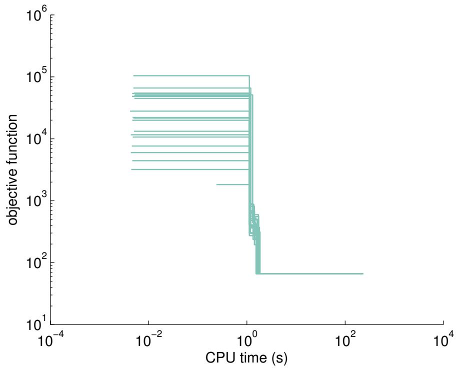

(a) Convergence of eSS2b with regularization

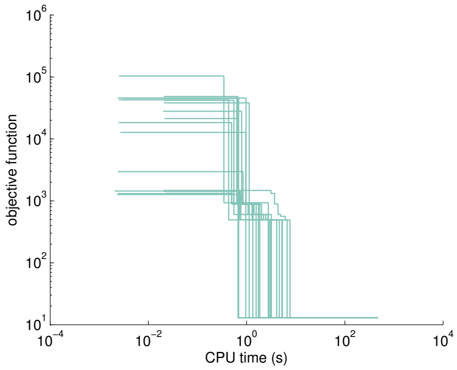

(b) Convergence of AMS

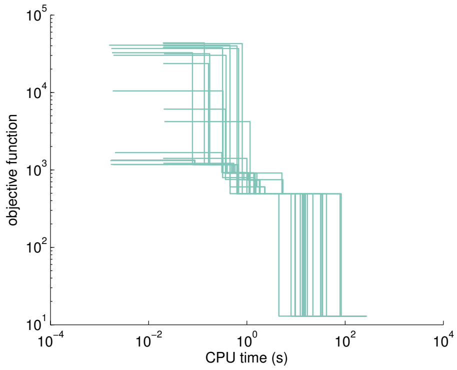

(c) Convergence of SMS

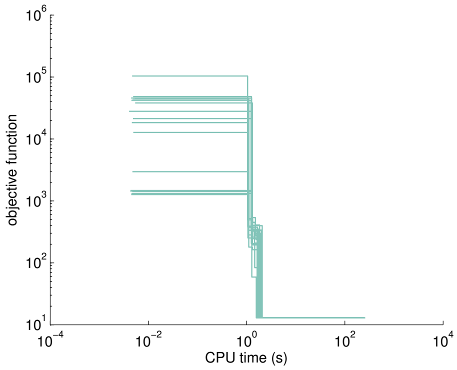

(d) Convergence of eSS2b

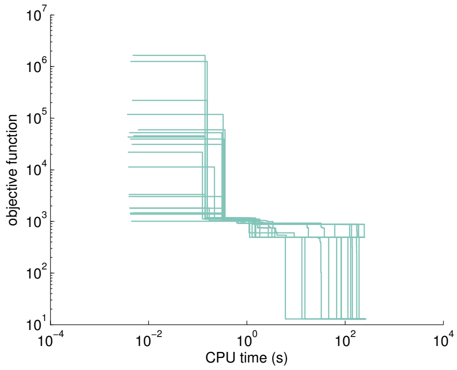

(e) Convergence of eSS2a

Figure S5.4.14: MAPK case study: convergence curves. SMS: simple multi start of NL2SOL; AMS: multistart of NL2SOL with logarithim Latin hypercube sampling, eSS2a: scatter search with NL2SOL and logarithmic initial guesses, eSS2b: scatter search with NL2SOL and logarithmic Latin hypercube initial guesses

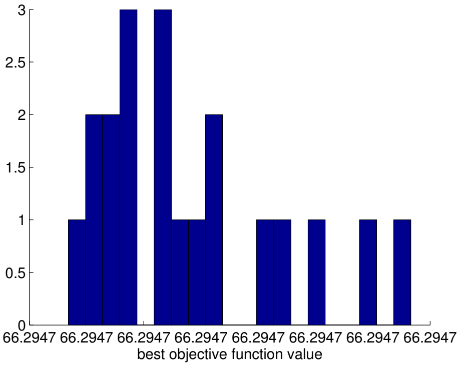

(a) Final values of eSS2b with regularization

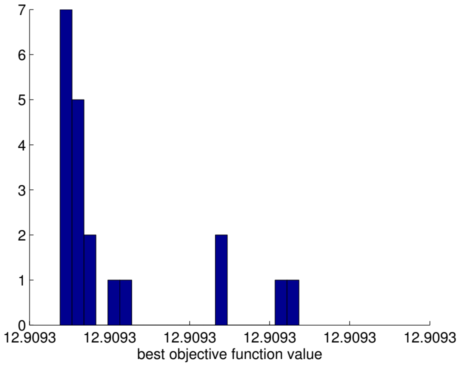

(b) Final values of AMS

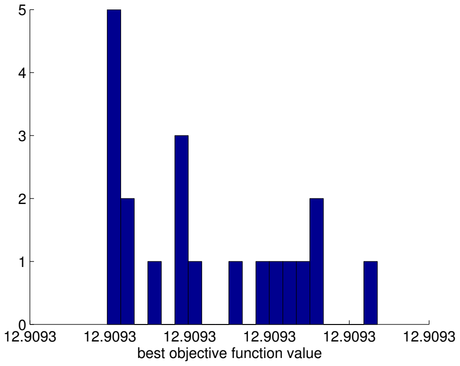

(c) Final values of SMS

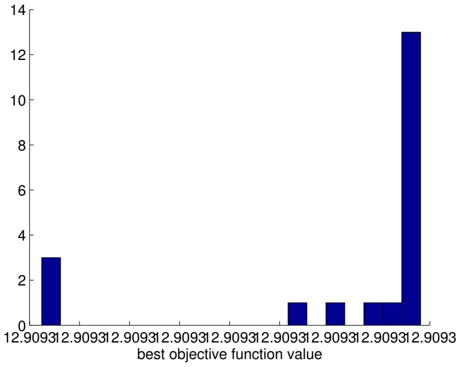

(d) Convergence of eSS2b

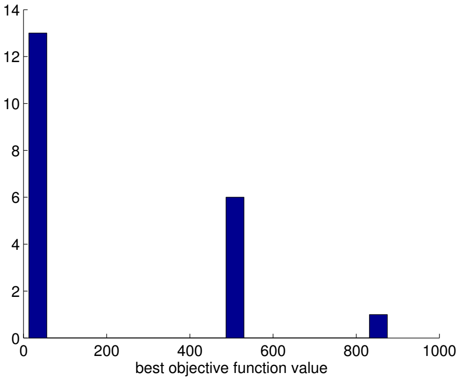

(e) Final values of eSS2a

Figure S5.4.15: MAPK case study: distribution of the final objective function values. SMS: simple multi start of NL2SOL; AMS: multistart of NL2SOL with logarithim Latin hypercube sampling, eSS2a: scatter search with NL2SOL and logarithmic initial guesses, eSS2b: scatter search with NL2SOL and logarithmic Latin hypercube initial guesses

S5.5 Goodwin Oscillator (GOsc)

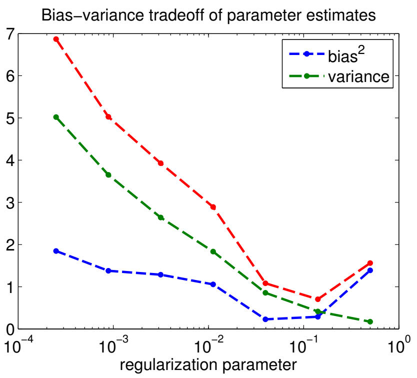

(a) Bias-variance trade-off for estimated parameters

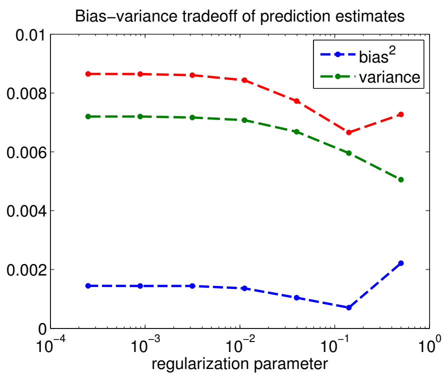

(b) Bias-variance trade-off for model prediction

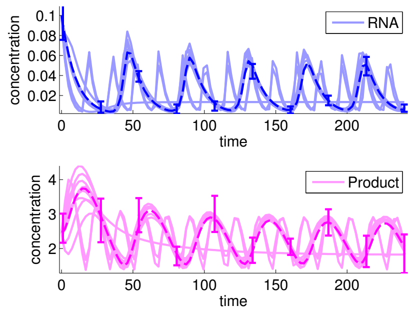

(c) Predictions of *non-regularized* model calibrations

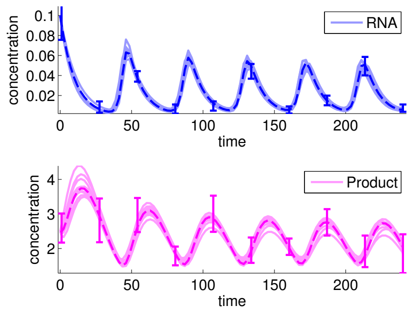

(d) Predictions of *regularized* model calibrations

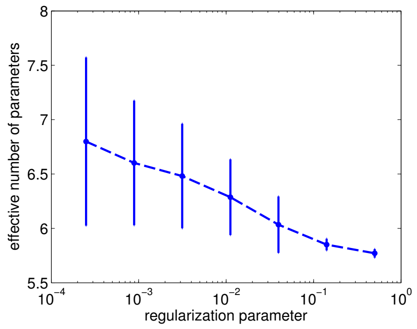

(e) Effective number of parameters

Figure S5.5.16: GOsc case study: (a,b) bias-variance trade-off for model estimated parameters and model predictions. (c,d) simulated trajectories of the calibrated models with and without regularization. (e) effective number of parameters

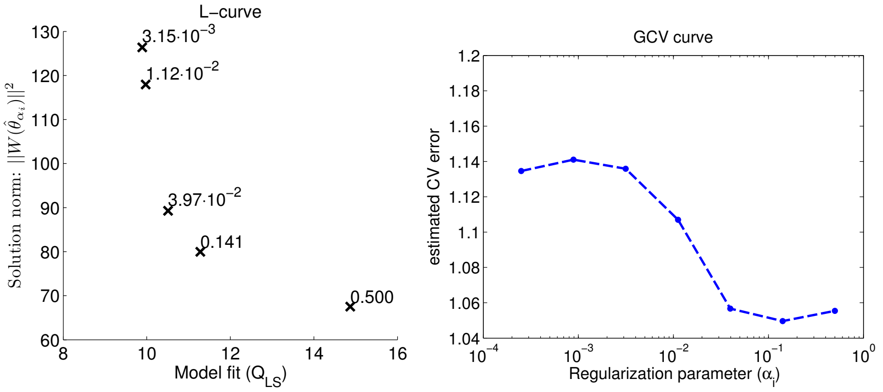

Figure S5.5.17: GOsc case study: model calibration. L-curve and generalized cross-validation curve. The selected regularization candidate corresponds to the minimum of the GCV curve.

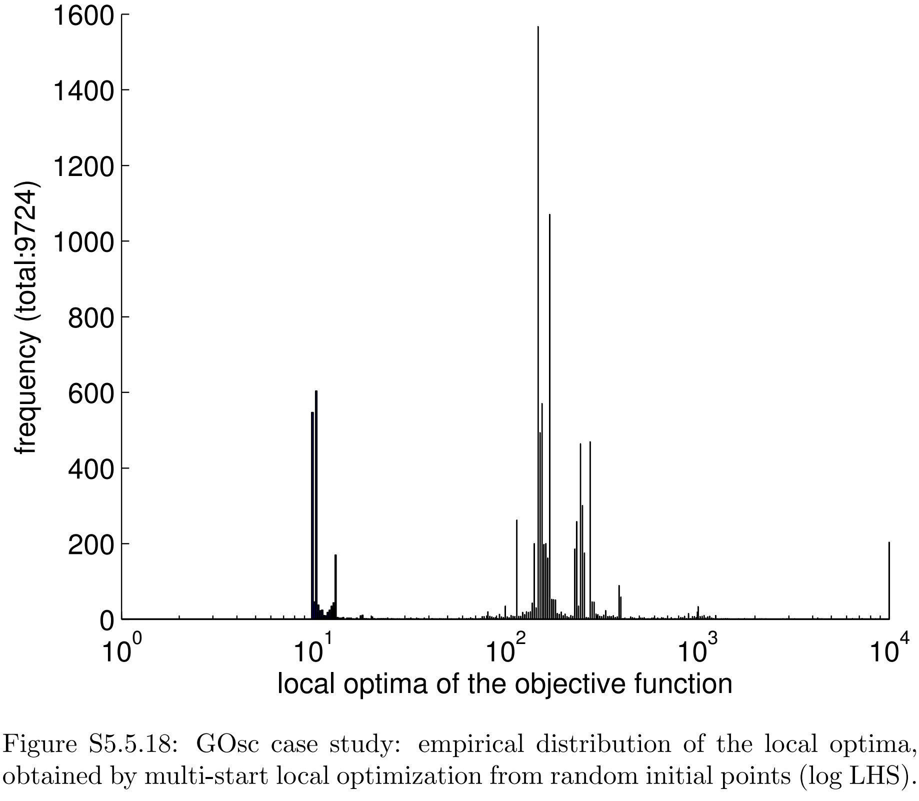

Figure S5.5.18: GOsc case study: empirical distribution of the local optima, obtained by multi-start local optimization from random initial points (log LHS).

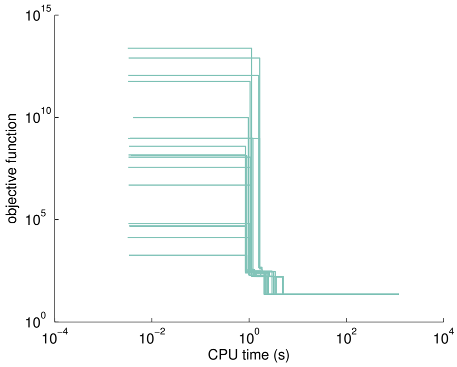

(a) Convergence of eSS2b with regularization

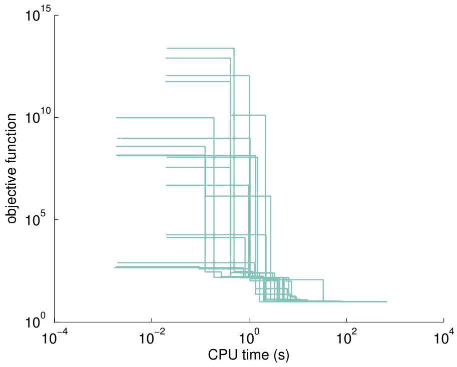

(b) Convergence of AMS

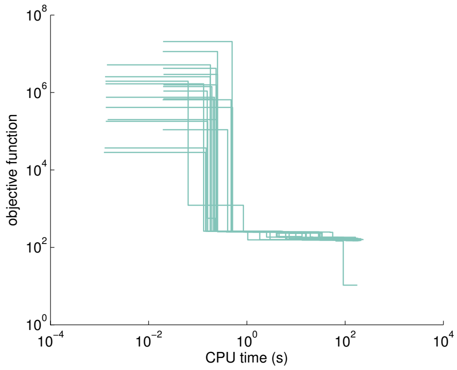

(c) Convergence of SMS

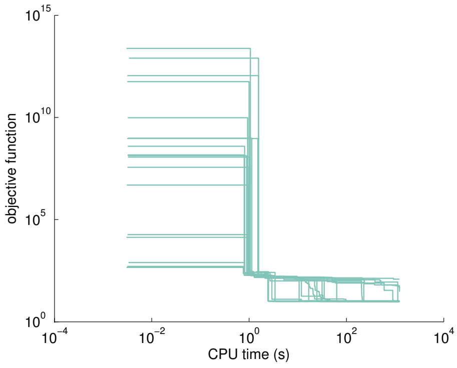

(d) Convergence of eSS2b

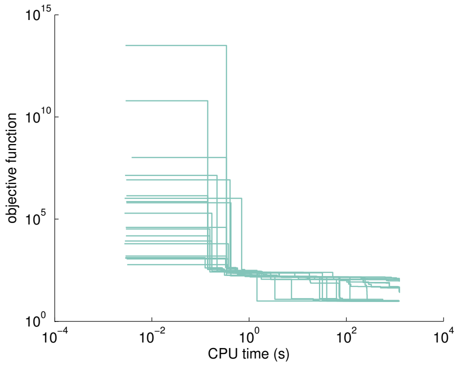

(e) Convergence of eSS2a

Figure S5.5.19: GOsc case study: convergence curves. SMS: simple multi start of NL2SOL; AMS: multistart of NL2SOL with logarithim Latin hypercube sampling, eSS2a: scatter search with NL2SOL and logarithmic initial guesses, eSS2b: scatter search with NL2SOL and logarithmic Latin hypercube initial guesses

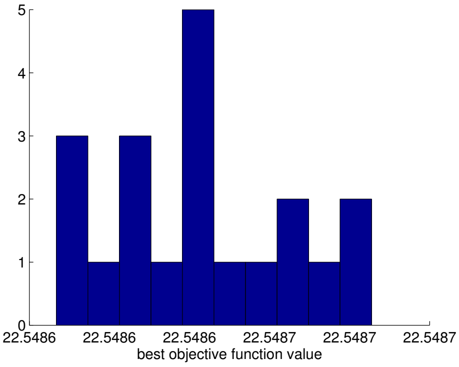

(a) Final values of eSS2b with regularization

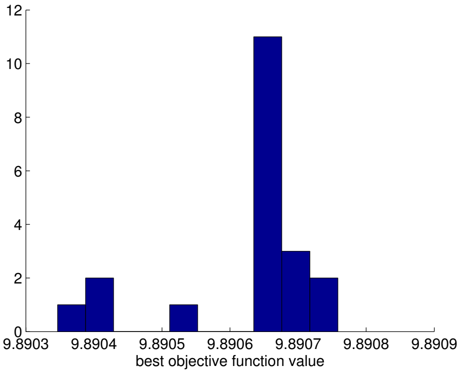

(b) Final values of AMS

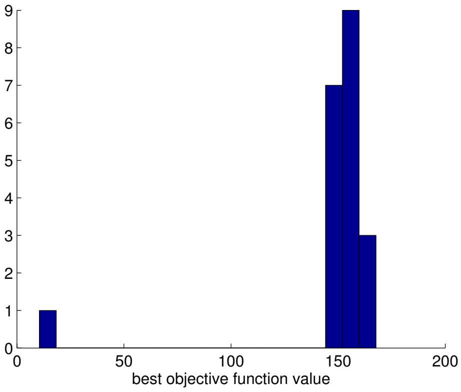

(c) Final values of SMS

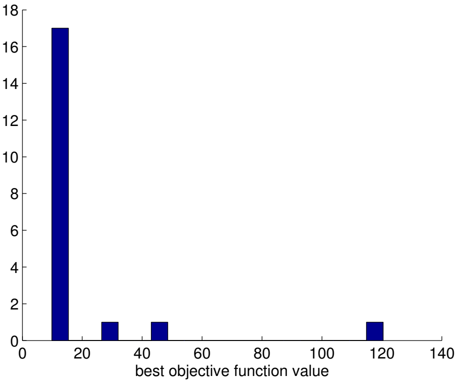

(d) Final values of eSS2b

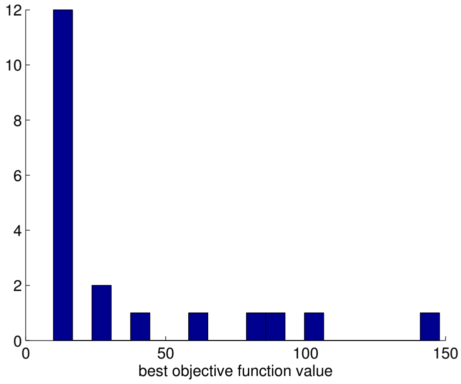

(e) Final values of eSS2a

Figure S5.5.20: GOsc case study: distribution of the final objective function values. SMS: simple multi start of NL2SOL; AMS: multistart of NL2SOL with logarithim Latin hypercube sampling, eSS2a: scatter search with NL2SOL and logarithmic initial guesses, eSS2b: scatter search with NL2SOL and logarithmic Latin hypercube initial guesses

S5.6 TGF– β signalling pathway model (TGFB)

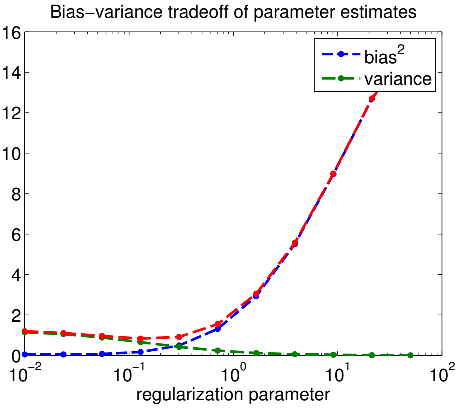

(a) Bias-variance trade-off for estimated parameters

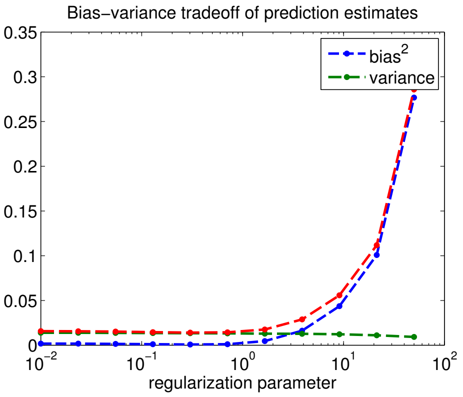

(b) Bias-variance trade-off for model prediction

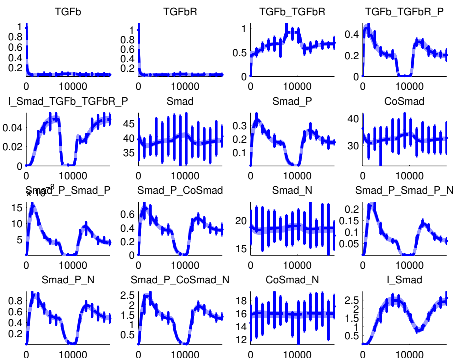

(c) Predictions of *non-regularized* model calibrations

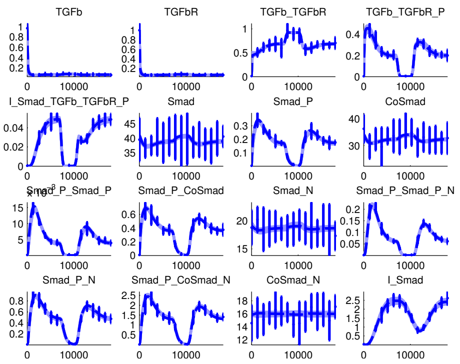

(d) Predictions of *regularized* model calibrations

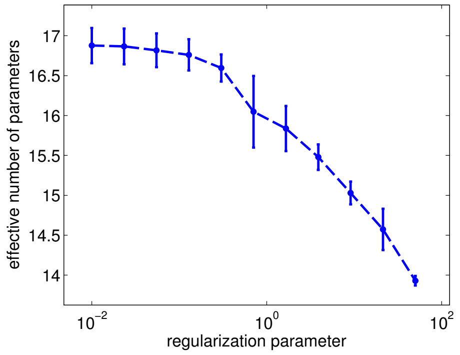

(e) Effective number of parameters

Figure S5.6.21: TGFB case study: (a,b) bias-variance trade-off for model estimated parameters and model predictions. (c,d) simulated trajectories of the calibrated models with and without regularization. (e) effective number of parameters

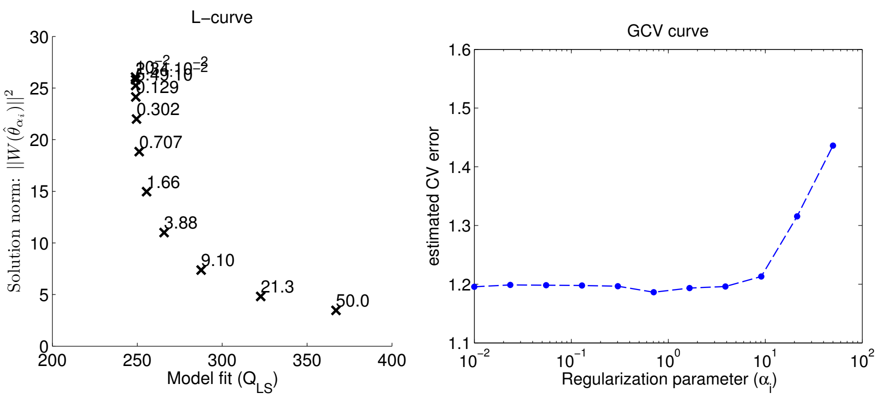

Figure S5.6.22: TGFB case study: model calibration. L-curve and generalized cross-validation curve. The selected regularization candidate corresponds to the minimum of the GCV curve.

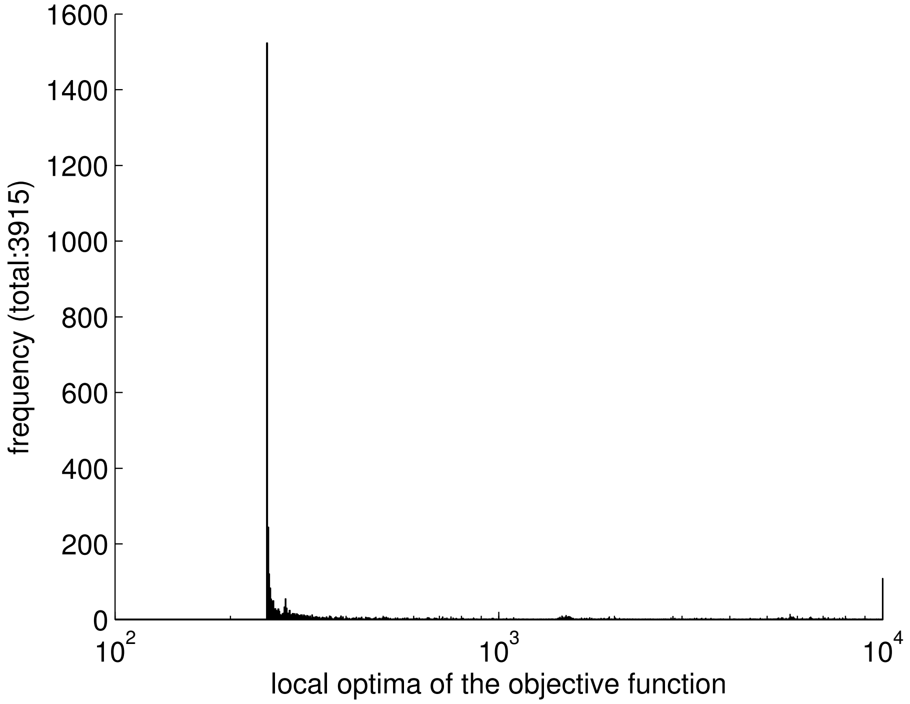

Figure S5.6.23: TGFB case study: empirical distribution of the local optima, obtained by multi-start local optimization from random initial points (log LHS).

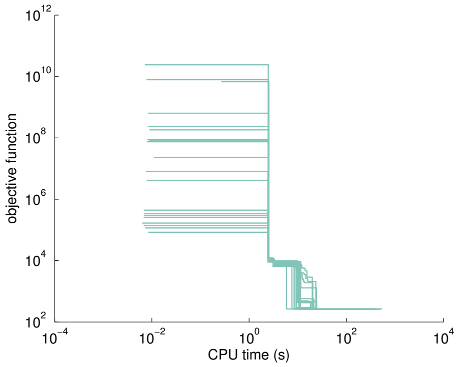

(a) Convergence of eSS2b with regularization

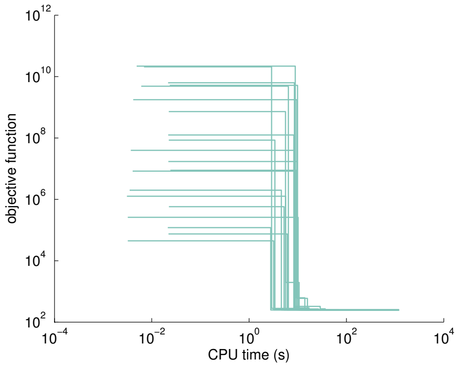

(b) Convergence of AMS

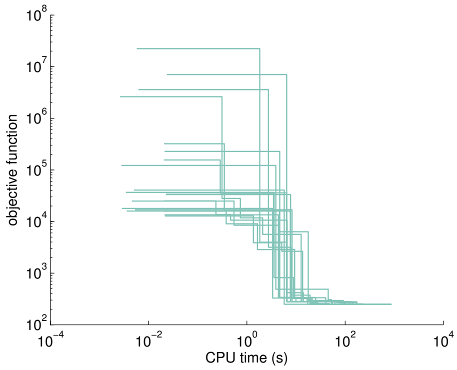

(c) Convergence of SMS

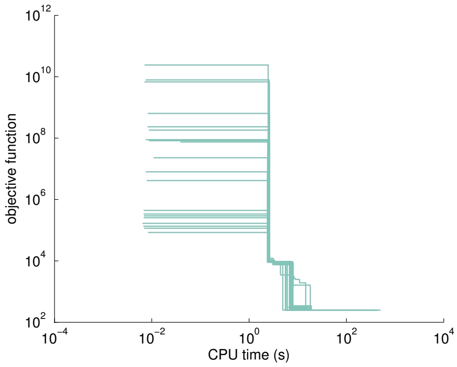

(d) Convergence of eSS2b

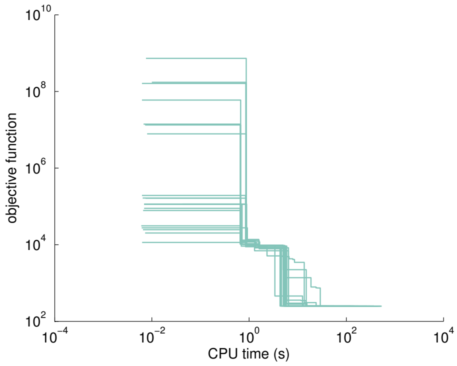

(e) Convergence of eSS2a

Figure S5.6.24: TGFB case study: convergence curves. SMS: simple multi start of NL2SOL; AMS: multistart of NL2SOL with logarithim Latin hypercube sampling, eSS2a: scatter search with NL2SOL and logarithmic initial guesses, eSS2b: scatter search with NL2SOL and logarithmic Latin hypercube initial guesses

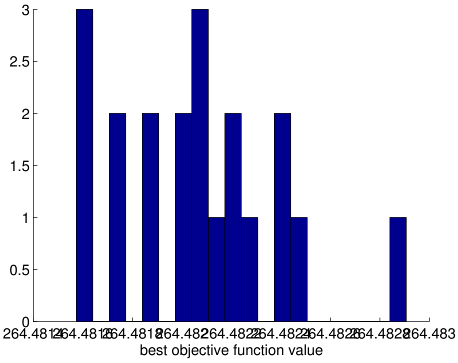

(a) Final values of eSS2b with regularization

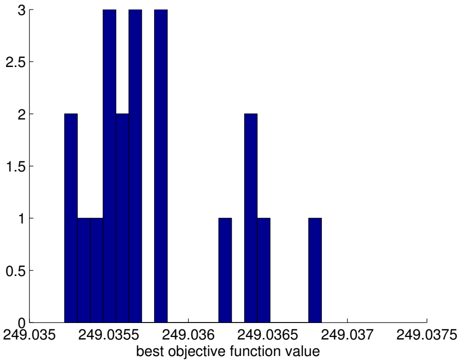

(b) Final values of AMS

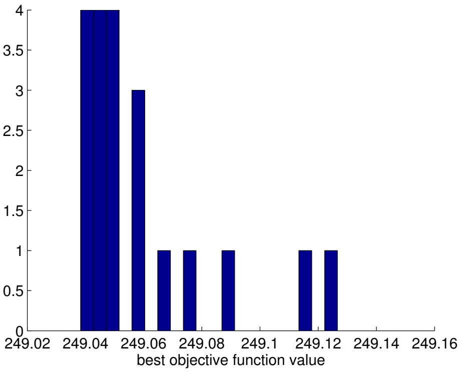

(c) Final values of SMS

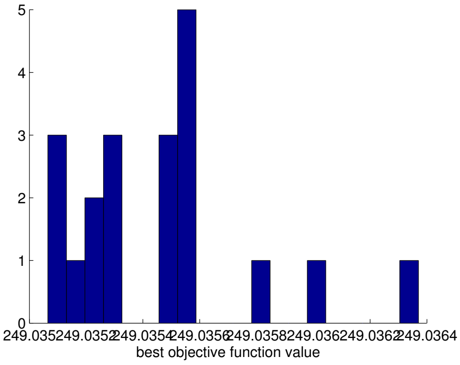

(d) Convergence of eSS2b

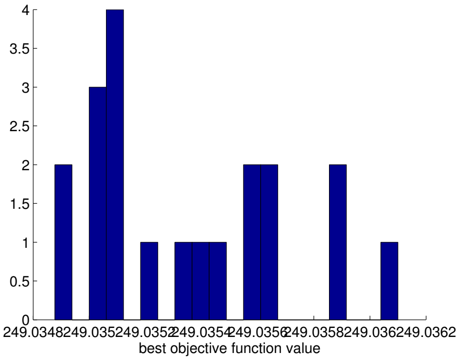

(e) Convergence of eSS2a

Figure S5.6.25: TGFB case study: distribution of the final objective function values. SMS: simple multi start of NL2SOL; AMS: multistart of NL2SOL with logarithim Latin hypercube sampling, eSS2a: scatter search with NL2SOL and logarithmic initial guesses, eSS2b: scatter search with NL2SOL and logarithmic Latin hypercube initial guesses

S5.7 3-Step Metabolic pathway model (TSMP)

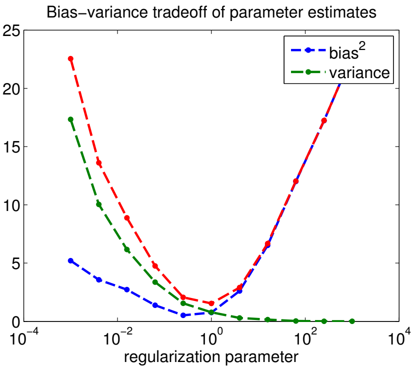

(a) Bias-variance trade-off for estimated parameters

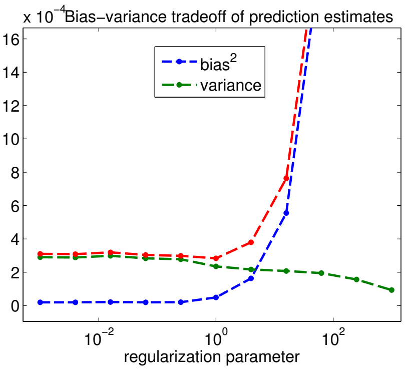

(b) Bias-variance trade-off for model prediction

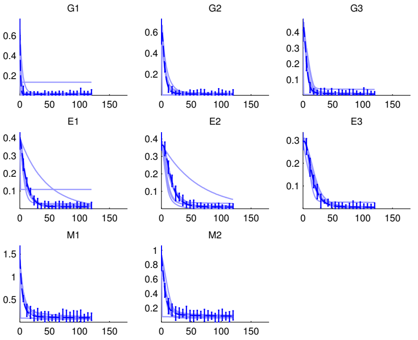

(c) Predictions of *non-regularized* model calibrations, experiment 1

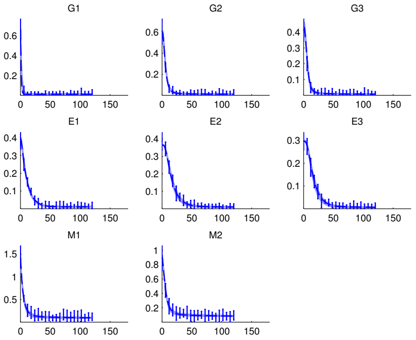

(d) Predictions of *regularized* model calibrations, experiment 1

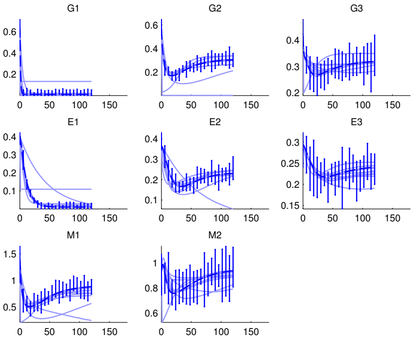

(e) Predictions of *non-regularized* model calibrations, experiment 2

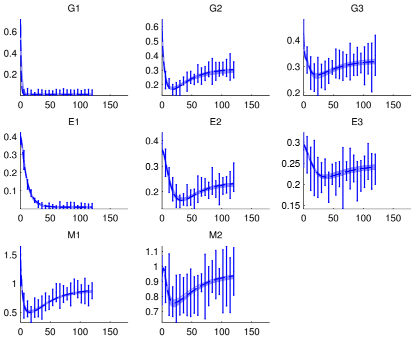

(f) Predictions of *regularized* model calibrations, experiment 2

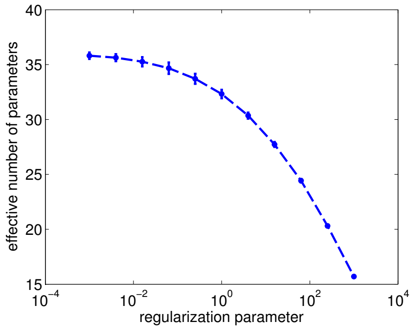

(g) Effective number of parameters

Figure S5.7.26: TSMP case study: (a,b) bias-variance trade-off for model estimated parameters and model predictions. (c,d) simulated trajectories of the calibrated models with and without regularization. (e) effective number of parameters

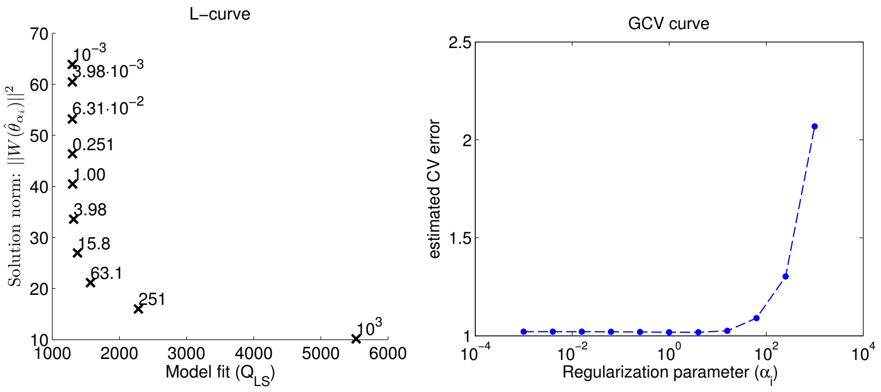

Figure S5.7.27: TSMP case study: model calibration. L-curve and generalized cross-validation curve. The selected regularization candidate corresponds to the minimum of the GCV curve.

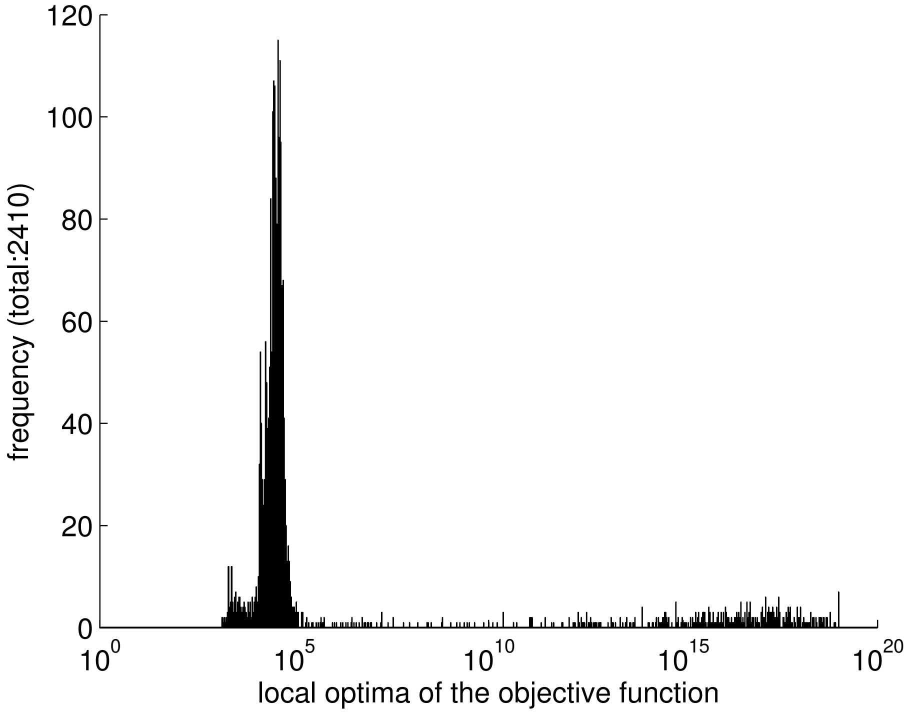

Figure S5.7.28: TSMP case study: empirical distribution of the local optima, obtained by multi-start local optimization from random initial points (log LHS).

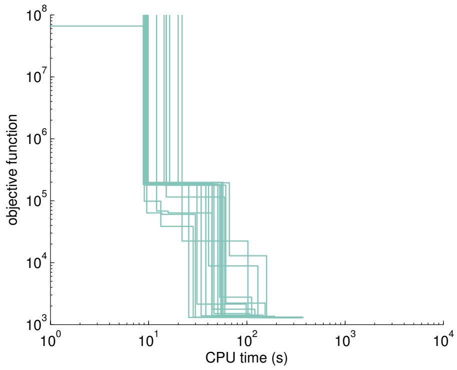

(a) Convergence of eSS2b with regularization

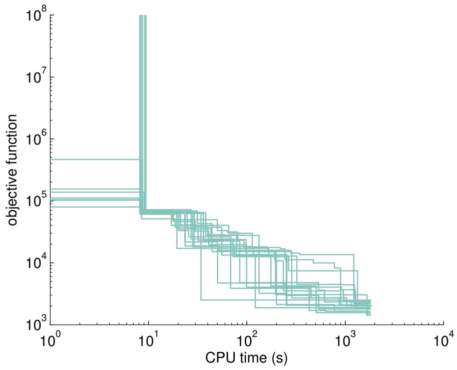

(b) Convergence of AMS

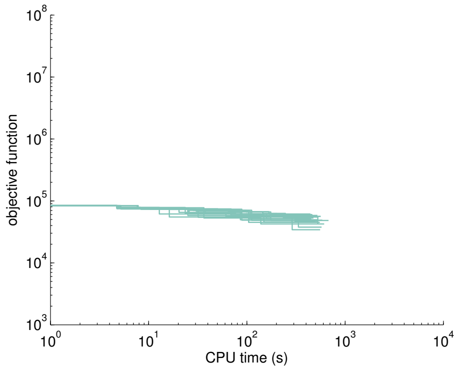

(c) Convergence of SMS

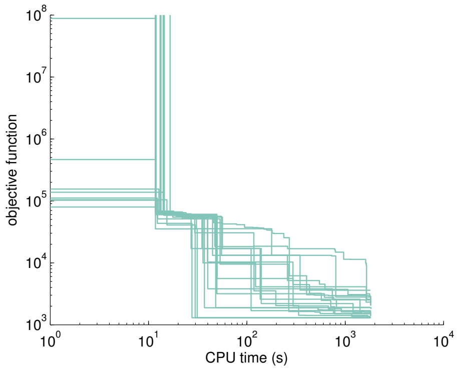

(d) Convergence of eSS2b

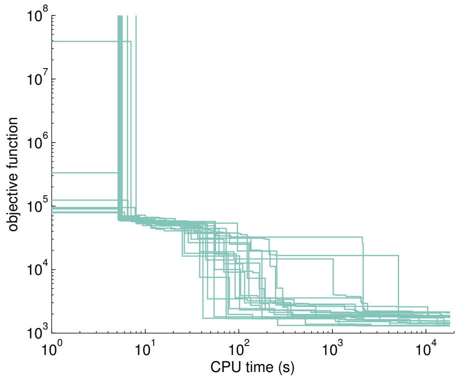

(e) Convergence of eSS2a

Figure S5.7.29: TSMP case study: convergence curves. SMS: simple multi start of NL2SOL; AMS: multistart of NL2SOL with logarithim Latin hypercube sampling, eSS2a: scatter search with NL2SOL and logarithmic initial guesses, eSS2b: scatter search with NL2SOL and logarithmic Latin hypercube initial guesses

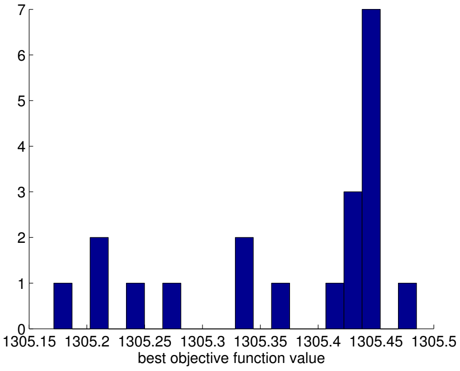

(a) Final values of eSS2b with regularization

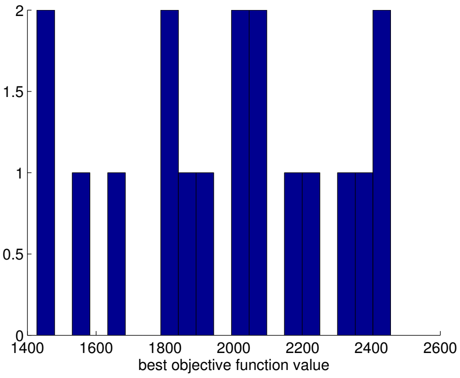

(b) Final values of AMS

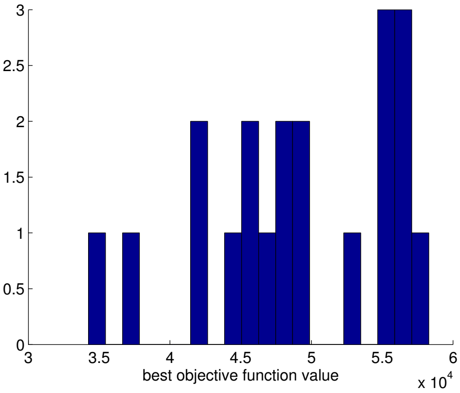

(c) Final values of SMS

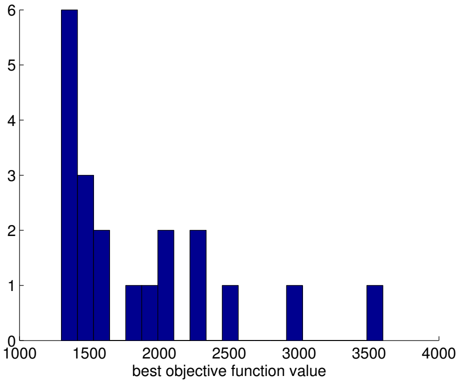

(d) Final values of eSS2b

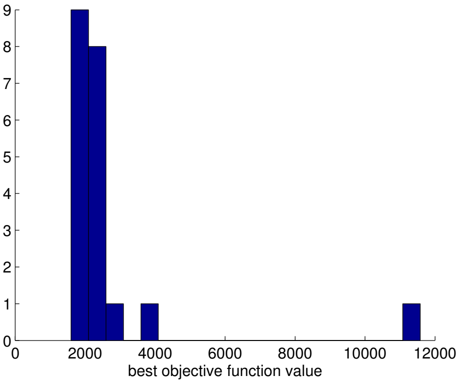

(e) Final values of eSS2a

Figure S5.7.30: TSMP case study: distribution of the final objective function values. SMS: simple multi start of NL2SOL; AMS: multistart of NL2SOL with logarithim Latin hypercube sampling, eSS2a: scatter search with NL2SOL and logarithmic initial guesses, eSS2b: scatter search with NL2SOL and logarithmic Latin hypercube initial guesses

S5.8 Chemotaxis signalling pathway model (CHM)

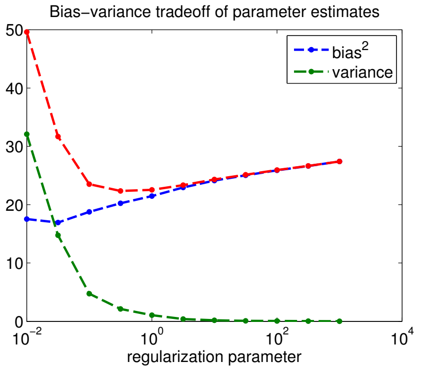

(a) Bias-variance trade-off for estimated parameters

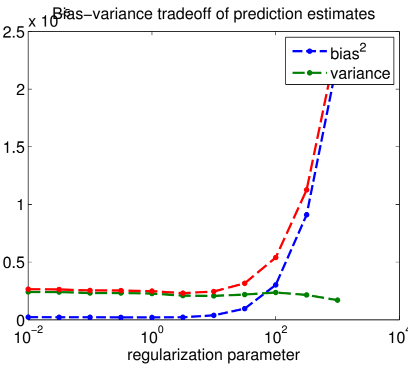

(b) Bias-variance trade-off for model prediction

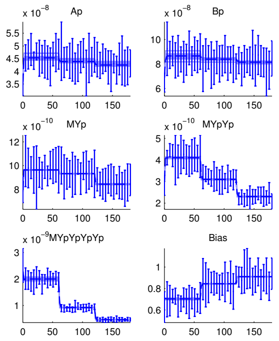

(c) Predictions of *non-regularized* model calibrations, experiment 1

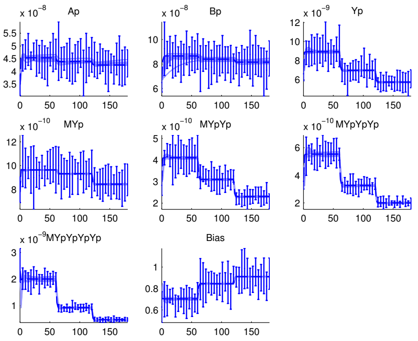

(d) Predictions of *regularized* model calibrations, experiment 1

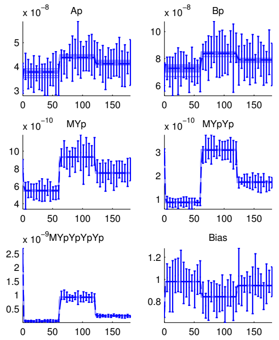

(e) Predictions of *non-regularized* model calibrations, experiment 2

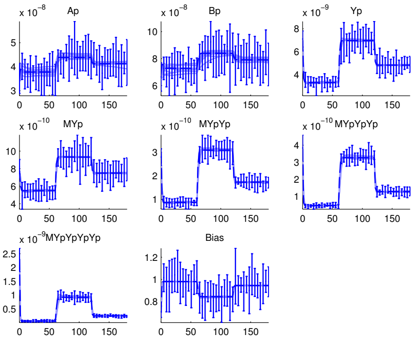

(f) Predictions of *regularized* model calibrations, experiment 2

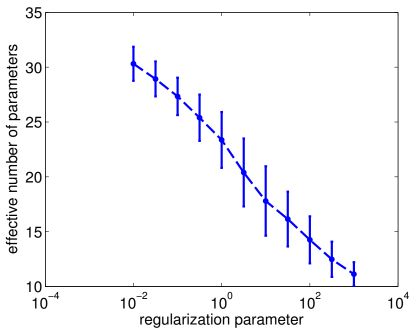

(g) Effective number of parameters

Figure S5.8.31: CHM case study: (a,b) bias-variance trade-off for model estimated parameters and model predictions. (c-f) simulated trajectories of the calibrated models with and without regularization. (g) effective number of parameters

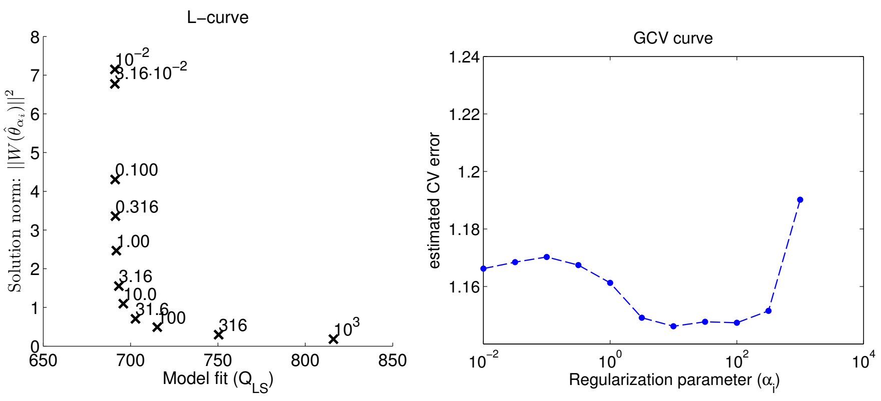

Figure S5.8.32: CHM case study: L-curve and generalized cross-validation curve. The selected regularization candidate corresponds to the minimum of the GCV curve.

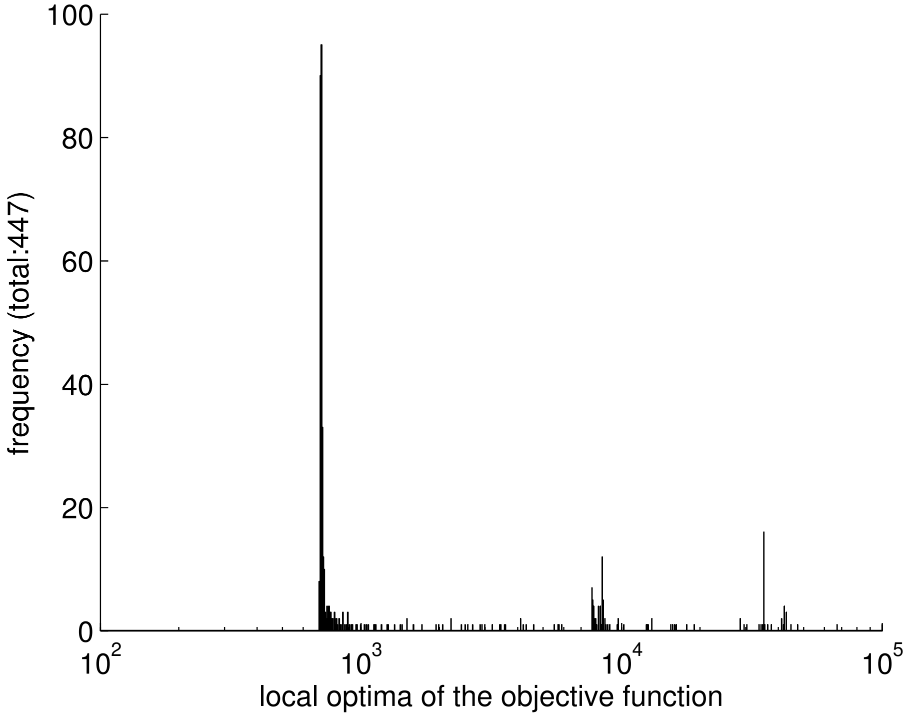

Figure S5.8.33: CHM case study: empirical distribution of the local optima obtained by multi-start local optimization from random initial points (log LHS).

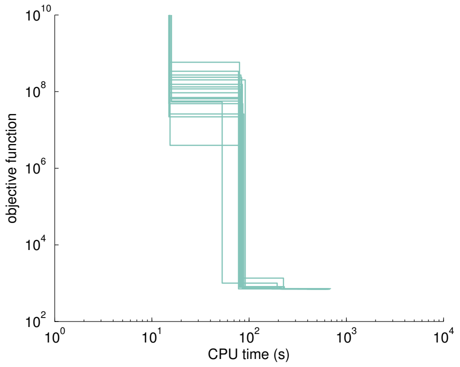

(a) Convergence of eSS2b with regularization

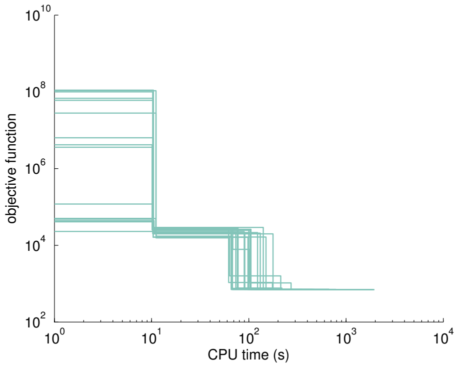

(b) Convergence of AMS

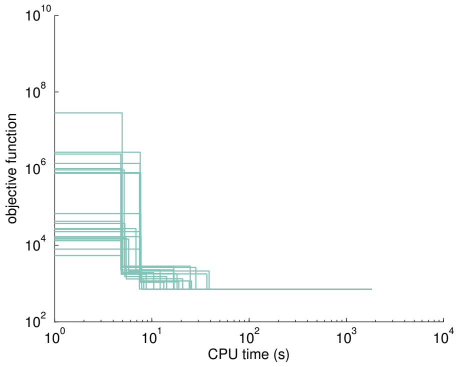

(c) Convergence of SMS

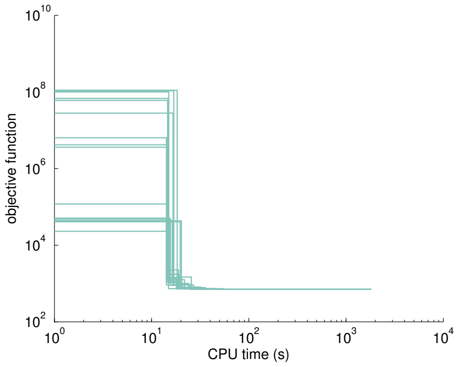

(d) Convergence of eSS2b

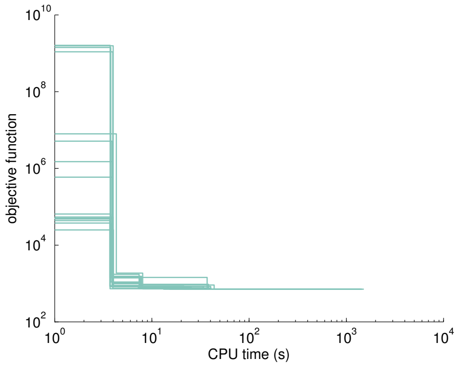

(e) Convergence of eSS2a

Figure S5.8.34: CHM case study: convergence curves. SMS: simple multi start of NL2SOL; AMS: multistart of NL2SOL with logarithim Latin hypercube sampling, eSS2a: scatter search with NL2SOL and logarithmic initial guesses, eSS2b: scatter search with NL2SOL and logarithmic Latin hypercube initial guesses

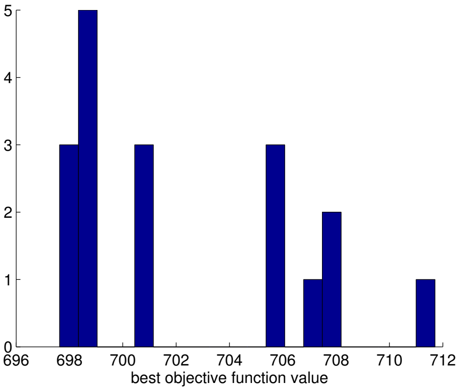

(a) Final values of eSS2b with regularization

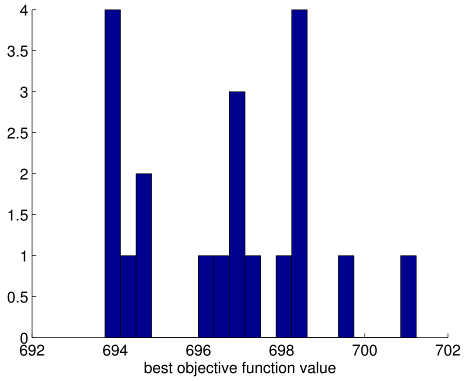

(b) Final values of AMS

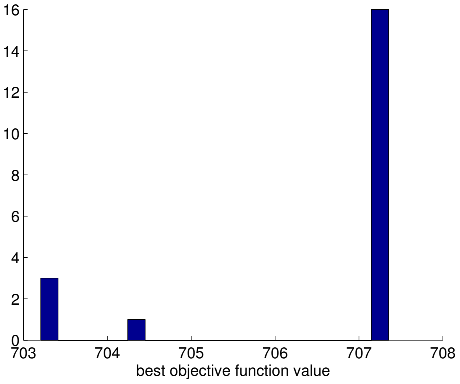

(c) Final values of SMS

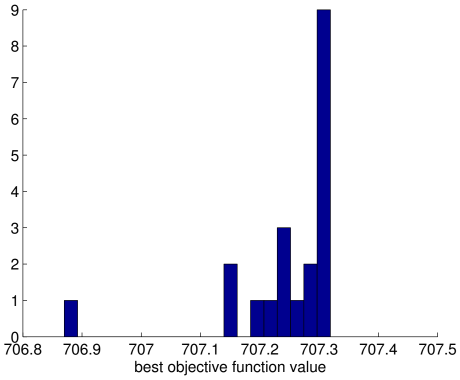

(d) Final values of eSS2b

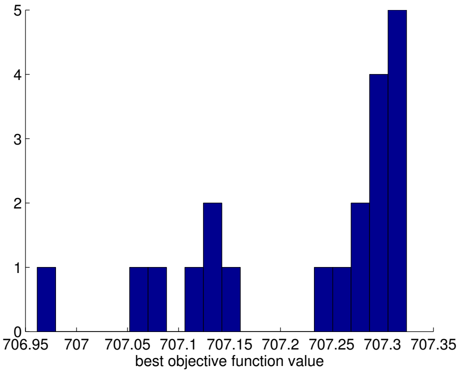

(e) Final values of eSS2a

Figure S5.8.35: CHM case study: distribution of the final objective function values. SMS: simple multi start of NL2SOL; AMS: multistart of NL2SOL with logarithim Latin hypercube sampling, eSS2a: scatter search with NL2SOL and logarithmic initial guesses, eSS2b: scatter search with NL2SOL and logarithmic Latin hypercube initial guesses

S5.9 Distributions of prediction errors - statistical test

In Section 3.3 of the main text the distributions of prediction errors of non-regularized models were compared to the regularized models. Each regularization scenario (worst, medium and best case) was compared to the non-regularized case (Figure 10 in main text). In order to check that the observed differences in the medians are significant, we use here the Wilcoxon rank sum test (also known as Mann-Whitney U test). This non-parametric statistical test can be used in situations where the normal distribution of the observed data points cannot be assumed. With this test we check the null-hypothesis (H_0) that regularization does not effect the prediction error, i.e. the prediction errors of the models calibrated without regularization are distributed in the same way as those calibrated with regularization. The alternative hypothesis is that the prediction errors are not distributed in the same way. In this case based on the rank sum values we can tell which median is significantly smaller than the other (higher rank sum means higher median) and therefore we can decide if regularization significantly improved the predictions. The results are summarized in Table S5.9.1, where we present the p-values corresponding the null-hypothesis and the result of the hypothesis tests for each regularization scenario and case study. When the null-hypothesis is rejected at the 0.05 significance level (indicated by a 1 in the H_0 column) we can conclude that the effect of regularization is significant on the model prediction performance.

The test results are mostly in agreement with the results obtained by visually comparing the medians in the box-plots in Figure 10 of the main text. However, they also allow us to see that, in the FHN case study, the observed differences in medians are statistically not significant. Further, in the case of the TGFB case study, the test shows that the best case regularization scenario significantly improves the performance even though the difference in the medians is small.

Table S5.9.1: Comparison of non-regularized and regularized model prediction errors by the Wilcoxon rank sum test. According to the null-hypothesis (H_0) the distribution of the prediction error with regularization is similar to the distribution of the prediction error without the regularization. The alternative hypothesis is that the regularization significantly effects the prediction error. The table shows the P-value corresponding to the hypothesis test, the result of the test on 0.05 level of significance (H_0 column) and the rank sum values for the non-regularized and regularized prediction errors respectively (larger value means worst prediction error in general). In case the H_0 is rejected (indicated by 1) and the rank sum corresponding to the regularization case (the second number in the pairs) is smaller, the regularization significantly improved the prediction.

	Regularization scenario								
Case study	Worst case			Medium case			Best case		
	P-value	H_0	Rank sum	P-value	H_0	Rank sum	P-value	H_0	Rank sum
BBG	0.6	0	(9836,10264)	0.18	0	(10601, 9499)	0.0035	1	(11245, 8855)
FHN	0.95	0	(10026,10074)	0.12	0	(10680, 9420)	0.15	0	(10640, 9460)
MAPK	0.72	0	(9903,10197)	$5.5 \cdot 10^{-8}$	1	(12274, 7826)	$2.7 \cdot 10^{-10}$	1	(12634, 7466)
GOsc	$1.6 \cdot 10^{-6}$	1	(12014, 8086)	$1.6 \cdot 10^{-8}$	1	(12365, 7735)	$2.4 \cdot 10^{-7}$	1	(12165, 7935)
TGFB	0.66	0	(10232, 9868)	0.15	0	(10644, 9456)	0.043	1	(10879, 9221)
TSMP	$1.3 \cdot 10^{-16}$	1	(13437, 6663)	$6.1 \cdot 10^{-7}$	1	(12092, 8008)	$4.00 \cdot 10^{-7}$	1	(12126, 7974)
CHM	0.0021	1	(8791,11309)	$1.1 \cdot 10^{-27}$	1	(14512, 5588)	$3.8 \cdot 10^{-21}$	1	(13913, 6187)
